# Causal interactions from proteomic profiles: Molecular data meet pathway knowledge

**DOI:** 10.1016/j.patter.2021.100257

**Published:** 2021-05-12

**Authors:** Özgün Babur, Augustin Luna, Anil Korkut, Funda Durupinar, Metin Can Siper, Ugur Dogrusoz, Alvaro Sebastian Vaca Jacome, Ryan Peckner, Karen E. Christianson, Jacob D. Jaffe, Paul T. Spellman, Joseph E. Aslan, Chris Sander, Emek Demir

**Affiliations:** 1 Computer Science Department, University of Massachusetts Boston, 100 William T. Morrissey Boulevard, Boston, MA 02125, USA; 2 cBio Center for Computational and Systems Biology, Dana-Farber Cancer Institute and Department of Cell Biology, Harvard Medical School, Boston, MA 02215, USA; 3 Department of Bioinformatics and Computational Biology, The University of Texas MD Anderson Cancer Center, Houston, TX 77030, USA; 4 Computational Biology Program, Oregon Health and Science University, 3181 SW Sam Jackson Park Road, Portland, OR 97239, USA; 5 Computer Engineering Department, Bilkent University, Ankara 06800, Turkey; 6 The Broad Institute of MIT and Harvard, Cambridge, MA 02142, USA; 7 Cogen Therapeutics, Cambridge, MA 02139, USA; 8 Department of Molecular and Medical Genetics, Oregon Health and Science University, 3181 SW Sam Jackson Park Road, Portland, OR 97239, USA; 9 Knight Cardiovascular Institute, Oregon Health and Science University, 3181 SW Sam Jackson Park Road, Portland, OR 97239, USA; 10 Pacific Northwest National Laboratories, 902 Battelle Boulevard, Richland, WA 99354, USA

**Keywords:** proteomics, causal pathway analysis, cancer

## Abstract

We present a computational method to infer causal mechanisms in cell biology by analyzing changes in high-throughput proteomic profiles on the background of prior knowledge captured in biochemical reaction knowledge bases. The method mimics a biologist's traditional approach of explaining changes in data using prior knowledge but does this at the scale of hundreds of thousands of reactions. This is a specific example of how to automate scientific reasoning processes and illustrates the power of mapping from experimental data to prior knowledge via logic programming. The identified mechanisms can explain how experimental and physiological perturbations, propagating in a network of reactions, affect cellular responses and their phenotypic consequences. Causal pathway analysis is a powerful and flexible discovery tool for a wide range of cellular profiling data types and biological questions. The automated causation inference tool, as well as the source code, are freely available at http://causalpath.org.

## Highlights

• CausalPath builds mechanistic models from proteomic profiles
• It integrates biological pathway models with molecular measurements
• It supports logical reasoning with post-translational modifications
• A web server, free software, and a source code are available

## The bigger picture

Molecular profiling of biological organisms provides us with a great amount of information on cellular differences, but converting it to mechanistic insights is still a very challenging task. A prominent approach is to integrate new measurements with the mechanistic knowledge described in the scientific literature and build a model that is supported by both. Although this can be done in many ways, an adept approach will use the literature knowledge in detail and follow high standards of logical reasoning while integrating the known and the new. This article describes an approach that utilizes the details in human biological pathways to identify pairs of changes with a likely cause-effect relation within. The approach automatically converts comparative proteomic and other molecular profiles into hypotheses of differentially active mechanistic relations that explain how the profiles came to be.

## Graphical abstract

**Figure:**
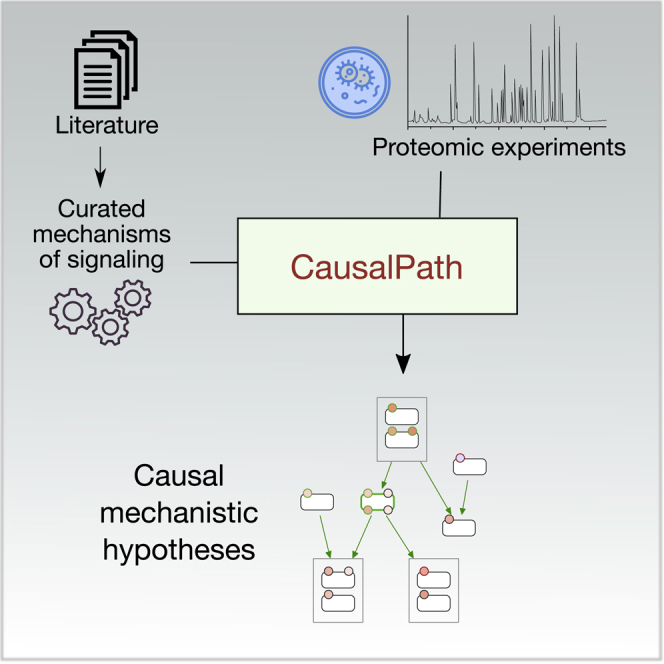

## Teaser

CausalPath integrates detailed biological pathways with proteomic and other molecular profiles to generate mechanistic models explaining how the observed changes are related. It is applicable to a wide range of contexts and a variety of experiment types. The method can be accessed at causalpath.org.

## Introduction

Central to a cell's decision-making processes is a vast network of biochemical reactions. A comprehensive, predictive model of cell biological mechanisms would revolutionize our scientific understanding and have tremendous clinical utility. Modeling efforts can be categorized roughly into two branches. The more established approach is to compile extensive, interconnected pathway models through the curation of reactions based on carefully designed low-throughput controlled experiments. This classic approach led to the first large-scale metabolic maps and later was extended to signaling and transcriptional processes. Today this knowledge is represented in hundreds of pathway and interaction databases (pathguide.org). The newer, data-driven inference approach leverages the recent developments in proteomics and other molecular technologies to directly infer graphical models, *ab initio*, from high-throughput measurements of controlled perturbations and natural variation.^1^, ^2^, ^3^

Both the classic and the data-driven inference approaches have inherent limitations. The classic curation approach uses well-validated fragments of knowledge, but these are extracted from a heterogeneous set of contexts, perturbations, conditions, and even organisms. The resulting models, even when carefully restricted to a particular context, are not well suited to making predictions. The purely data-driven inference approaches, on the other hand, create context-specific, predictive models, but they do not scale in terms of statistical power as the model space is exponentially larger than the observable space.

A strategy to alleviate the power issue of the data-driven approach is to get help from prior knowledge when the perturbations in the data are not sufficient to decide between alternative models.^4^, ^5^, ^6^, ^7^, ^8^, ^9^, ^10^ The methods that use this strategy, however, use prior knowledge in a reduced form, such as simple interaction networks, omitting mechanistic details and their logical harmony with the new data. The more an experiment lacks extensive perturbations, the more it can benefit from prior knowledge. Considering that the vast majority of currently available proteomic experiments have either few perturbations (e.g., before/after a stimulation) or only uncontrolled variation (e.g., profiles from disease cohorts), it is very important that we use prior knowledge in its full potential. In this perturbation-poor setting, model-building activity is transformed into selecting parts of the prior knowledge that can best explain the shape of the data, which we call “pathway extraction.” Here, we present a pathway extraction method, CausalPath, which uses the rich semantics of curated pathway knowledge, including the type of mechanism, the direction, signs of effect, and post-translational modifications. The inferred mechanisms are falsifiable hypotheses that can be experimentally interrogated.

**Figure 1:**
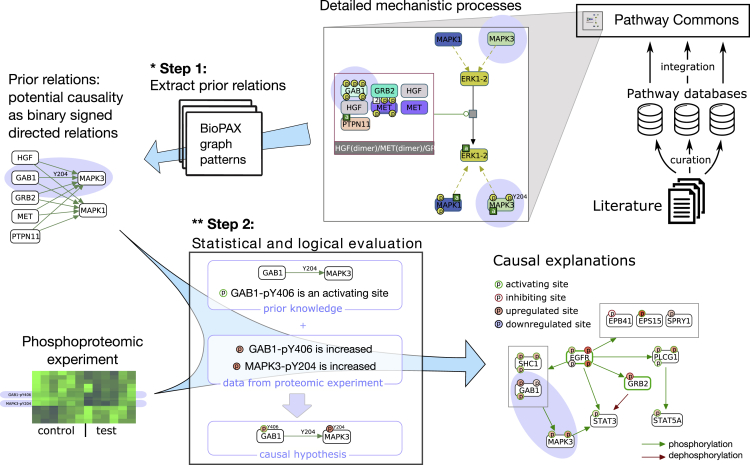
Overview of CausalPath pipeline over an example analysis One relationship CausalPath generated from the EGF stimulation study was GAB1 → MAPK3. Prior information for this relationship was curated into pathway databases, which we integrate into Pathway Commons as detailed mechanistic processes. We detect structural patterns in these processes that indicate that GAB1, when activated through phosphorylation, can, in turn, help in phosphorylation of MAPK3 (step 1). These phosphorylations were correlated in the proteomics dataset in the direction compatible with the prior, so CausalPath selects this relationship as a potential explanation (step 2). The final logical network is a subgraph of the EGF stimulation analysis results at 2 min time frame. For a more comprehensive description of graph notation, please see Figure 3C. We omit phosphorylation site locations while rendering the resulting network for complexity management; these can be inspected interactively within the CausalPath on causalpath.org. (This figure provides conceptual examples for steps 1 and 2 of CausalPath. ∗Step 1 recognizes a variety of pathway structures that can causally link an upstream protein activity to a downstream proteomic feature, which are detailed in Data S1. ∗∗Step 2 checks if the direction of the measured proteomic changes is compatible with the expectations set by the prior information using Equations 1 and 2 [see main text]. Step 2 is demonstrated in more detail in Figure S1.)

CausalPath maps proteomic profiles to curated human pathways from multiple resources that are integrated into the Pathway Commons database,^11^ detects the potential causal links in the pathways between measurable molecular features using a graphical pattern search framework, and identifies the subset of the causal links that can explain correlated changes in a given set of proteomic and other molecular profiles. These explanations are presented as an intuitive network with links to the detailed prior knowledge models and the related literature to create a powerful exploration and analysis platform (Figure 1). This approach in some sense mimics a literature search of a biologist for relationships that explain relationships in his or her data. The method takes into account hundreds of thousands of curated mechanisms, which would be infeasible to do manually. We demonstrate the value of CausalPath on multiple publicly available datasets covering a wide range of scenarios and biological questions: in a set of time-resolved epidermal growth factor (EGF) stimulation experiments we detected EGFR activation with its signaling downstream of MAPKs, including feedback inhibition on EGFR; from ligand-induced and drug-inhibited cell-line experiments, we estimated the precision of CausalPath predictions; from CPTAC (Clinical Proteomic Tumor Analysis Consortium) protein mass spectrometry datasets for ovarian and breast cancer we elucidated general and subtype-specific signaling, as well as regulators of well-known cancer proteins; and in RPPA (Reverse Phase Protein Array) experimental datasets of 32 TCGA (The Cancer Genome Atlas) cancer studies we found a core signaling network that is recurrently identified across many cancer types. These models bring new insights into cancer biology in terms of differences and commonalities of cancers in signaling. CausalPath is freely available to researchers through its website at causalpath.org for analysis of new proteomic experiments.

## Results

### Design and properties of CausalPath

The CausalPath workflow has two main steps: (1) detection of causal priors from pathway databases, performed once and reused in multiple analyses, and (2) matching causal priors with supporting correlated changes in the analyzed data, performed for every analysis. We define a “causal prior” as a set of prior knowledge that as a group suggests a possible causal link between two measurable molecular features.

Existing kinase-substrate databases and transcription factor-target databases are valuable sources for causal priors, but they capture only a small part of the known biology; hence, they are limited for comprehensive causal reasoning. There are other databases that take a more detailed modeling approach for biochemical processes, such as Reactome. The Pathway Commons database provides integration of such detailed models collected from publicly available resources in the format of the BioPAX modeling language. Such models include details like post-translational modifications, molecular complexes, abstractions such as homologies, involvement of small molecules in signaling, and so on. Detailed process models provide a great opportunity to identify causal relations between the molecular measurements, but they require sophisticated algorithms to reason over them.

To detect the causal prior relations, i.e., structures that imply causal relationships between proteins in the Pathway Commons database, we used the BioPAX-pattern software^12^, and manually curated 12 graphical patterns (described in Data S1). Each graphical pattern captures the control mechanisms over either a phosphorylation of a protein or the expression of a gene. Searching for these patterns in Pathway Commons generated 28,517 prior relations in four different types (listed below). To increase coverage, we added relations from several other databases (PhosphoNetworks,^13^ iPTMnet,^14^ TRRUST,^15^ and TFactS),^16^ which are not in Pathway Commons, and increased our relationships to 39,232:

**Table:** 

Relation type	Extracted from Pathway Commons	After addition	Details
Phosphorylation	20,020	24,430	from 2,230 proteins to 3,356 targets
Dephosphorylation	2,766	2,766	from 925 proteins to 338 targets
Expression upregulation	4,921	9,032	from 1,558 proteins to 1,915 targets
Expression downregulation	810	3,004	from 875 proteins to 1,018 targets

The imbalance in the number of relations reflects the representation bias of these relationship types in the scientific literature. We assessed the overlap of these prior relations with the “canonical pathways” gene sets in MSigDB to understand its coverage. This collection has 2,815 gene sets curated from the databases BioCarta, KEGG, NCI-PID, Reactome, and WikiPathways. The genes in our prior relations have a nonzero overlap with 99% of these gene sets. If we redefine “overlap” focusing on relations instead of genes, and require that both the source and the target gene of a relation be in a gene set to count as overlap, then our prior relations have nonzero overlap with 68% of the canonical pathway gene sets.

We define a “causal conjecture” as a pairing of a causal prior with supporting measurements in the molecular dataset that together declare that “one molecular change is the cause of another molecular change.” The *change* here can be detected in two different forms based on the experimental setting: it can be up/downregulation for individual features in a “test versus control” comparison setting, or it can be positive/negative correlations applying to pairs of features in an uncontrolled study, as is common in cancer biology. We call an analysis in the former setting “comparison-based” and the latter “correlation-based.” As an example of comparison-based generation of a causal conjecture, consider the following chain of items from a study that detects a set of proteomic changes after stimulation by EGF:

**Table:** 

1. GAB1-pY406 peptide level is increased in response to EGF stimulation.	(from data)
2. Y406 is an activating phosphorylation site of GAB1.	(from prior knowledge)
3. GAB1 is part of a complex that can phosphorylate MAPK3 at Y204.	(from prior knowledge)
4. The MAPK3-Y204 peptide level is increased in response to EGF stimulation.	(from data)

Items 1 and 4 are direct observations from proteomic profiles and they are observed within the EGF stimulation context, and items 2 and 3 are the knowledge fragments that constitute the causal prior, as reported in publications from other experiments and subsequently curated into pathway databases. The causal conjecture here is that the increase in phospho-GAB1 (GAB1-pY406) after EGF stimulation causes an increase in its activity of helping phosphorylation of MAPK3 and hence an increase in the level of MAPK3-Y204. This is a well-defined, mechanistic, and falsifiable conjecture that is easily testable by perturbations (see Figure S1 for a complete iteration of different forms of causal conjectures used in this study). The important aspect here is that this conjecture is automatically generated, rather than inferred by a researcher.

In the case of a correlation-based analysis, we replace items 1 and 4 with an observed correlation, e.g., “Measured peptide levels of GAB1-pY406 are positively correlated with the peptide levels of MAPK3-Y204,” for a *correlation-based* causality hypothesis.

(Equation 1) $$\;\bar{c_{source}⊕e_{source}⊕s_{relation}⊕c_{target}}=true\;\;\;\left(\text{comparison-based}\right)$$

(Equation 2) $$\;corr_{source,target}⊕\left(e_{source}⊕s_{relation}\right)=true\;\;\;\left(\text{correlation-based}\right)$$

To formalize and generalize the example of causal conjecture detection in comparison-based analysis, we can formulate it with a ternary logical equation (Equation 1), where ⊕ is a ternary XOR operation, the overline is logical negation, *c* represents the change direction of the gene features, *e* represents the effect of the source feature on its activity, and *s* represents the sign of the pathway relation, where c,e,s∈{true,false,unknown}. The four terms in the equation correspond to the four items in the example, which collectively test if the data are consistent with a known causal interaction. The change of gene features are true in the case of upregulation, false in the case of downregulation, and unknown in the case of insignificant. The effect of source feature $e_{source}$ is true in the case of total protein or activating phosphorylation, false in the case of inactivating phosphorylation, and unknown if it is a phosphorylation site with unknown effect. The relation sign, $s_{relation}$, is true for phosphorylation and expression upregulation and false for dephosphorylation and expression downregulation. Any ⊕ operation on unknown value will yield an unknown result, hence the equation does not hold if any value is unknown. In the case of correlation-based causality, instead of the terms $c_{source}$ and $c_{target}$, we use the logical representation of the sign of the correlation ($corr_{source,target}$), where true represents positive correlation and false represents negative correlation (Equation 2). In addition to the logical check by these equations, we limit the phospho regulations (phosphorylation and dephosphorylation) to the explanation of phosphoprotein changes and limit the expressional regulations to the explanation of total protein changes (and optionally mRNA changes).

On top of the logic-based detection of causal interactions, we provide two types of statistical measurements to increase the interpretability of the results. “Network-size test” checks if the correlated changes align with the causal priors in general, which is indicated by a larger number of interactions in the results than would arise by random chance, which we test by data label randomization. “Downstream-size test” checks if a protein on the network has more downstream targets in the results than expected by chance using the same randomization procedure. Significant values from these two tests provide additional evidence suggesting the data are shaped by the priors or that a protein has an influence on the significant number of targets, respectively, which consequently increases our confidence in the results.

### Testing and validation of the method

We performed three studies to evaluate the method's performance and understand its characteristics: (1) To demonstrate the method on a simple test case, we reanalyzed proteomic profiles from an EGF stimulation experiment. (2) We measured the precision of CausalPath results, where we analyze ligand stimulation of four different breast cancer cell lines and test the predictions with protein inhibitors. (3) Using a proteomic experiment that measures the effects of 31 different drugs on PC3 cell lines, we measured CausalPath's ability to relate observed changes to altered drug targets.

We provide analyses for the robustness and reproducibility of CausalPath results, as well as a survey of other methods related to pathway analysis for proteomic datasets in Data S1.

#### Analysis of EGF stimulation on EGFR Flp-in cells

We reanalyzed a recent cell-line EGF stimulation phosphoproteomic dataset^5^ to see if CausalPath can identify downstream events of EGF signaling. The experiment provides mass spectrometry profiles at eight time points, where a total of 1,068 phosphopeptides are measured. We compared each time point with the initial time point (unstimulated cells) to see how the EGF stimulus is propagated over time. Since the data are phosphopeptide only and do not contain any observable change on EGF itself, we included EGF activation as a “hypothesis” to the analysis. At the initial time points, CausalPath detects many EGFR targets and relates them to EGFR phosphorylation and activation. As one expects, both EGF and its receptor EGFR downstream are significantly enriched with changes that indicate their activation. At the fifth time point (16 min), we observe inhibitory feedback phosphorylation of EGFR explainable by MAPK1 and MAPK3 activity, followed by the dramatic dampening of EGF signaling. All the networks up to the fifth time point are significant in size (p < 0.0001).

Interestingly, an explanation for MAPK1/3 phosphorylation is missing in this result. It is known that EGF signaling can activate MAPK1/3 through several steps and multiple paths, but none was captured. In its most strict configuration, CausalPath forces phosphorylation sites in the literature to exactly match the detected sites in the phosphoproteomic data. When we slightly relax this constraint by allowing two amino acids difference in site locations, we detect that SHC1 and GAB1 phosphorylations can causally link EGF stimulation to MAPK3 phosphorylation (Video S1). Site locations reported in the literature are sometimes shifted relative to the sequence of the canonical protein isoform provided by UniProt. For example, it is relatively common in the literature to omit the initial methionine on the protein, which is often cleaved, but UniProt uniformly includes these methionines in its reference sequence. We are actively working on curation-correction tools for addressing these problems in the future. As a stop-gap measure, CausalPath's option to slightly relax the site matching is useful for most applications.

Video S1. CausalPath analysis of the EGF stimulation experiment Each frame of the animated GIF corresponds to a time point in the experiment, which is compared with the initial control time point.

#### Precision of CausalPath results on cell lines stimulated with ligands

We used a recently published RPPA experiment to estimate the precision of CausalPath results. This experiment stimulates four different breast cancer cell lines with seven different ligands, and also treats each cell line/ligand combination using five targeted drugs.^17^

**Table:** 

Cell lines	Ligands	Inhibitor drugs
BT20	EGF	AZD8055 targeting MTOR
BT549	FGF1	BEZ235 targeting PIK3CA and MTOR
MCF7	HGF	GSK690693 targeting AKT
UACC812	IGF1	GSK1120212 targeting MEK
	insulin	PD173074 targeting FGFR
	NRG1	
	PBS∗	

∗The case of PBS represents the lack of ligands that are naturally found in bovine serum that is used as control in all cases.

**Figure 2:**
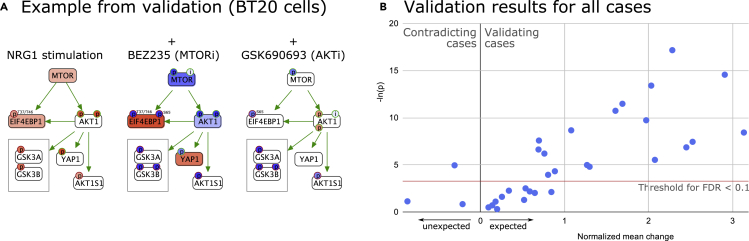
Validation of CausalPath relations on a cell-line ligand-stimulation and drug-inhibition RPPA dataset (A) An example subnetwork from CausalPath results to illustrate how the validation works. The first subnetwork is generated by comparing NRG1-stimulated BT20 cells with the unstimulated control cells. Since this network nominates activated MTOR and AKT1 as the cause of several downstream phosphorylations, we can test these hypotheses using MTOR and AKT inhibitors. The next two graphs show the same subnetwork after the inhibitors are applied (ligand+/inhibitor+ cells are compared with ligand+/inhibitor− cells). See Figure 3C for graph legend. (B) Cumulative validation results from all 32 cases, generated by readouts from 14 distinct antibodies. The x axis has mean changes in the antibody readouts normalized to their global standard deviation and expected direction. A positive value indicates the change is in the expected direction.

We first ignored the drug inhibition samples, and used the ligand stimulation experiments to predict their associated causal relations using CausalPath. Then, we identified the relations in the result networks whose source protein is targeted by one of the inhibitor drugs in the study. For these relations, the inhibition experiments provide validation of their inference. When the drug targeting the upstream protein is applied, if the CausalPath result relation is valid, we expect to see a reverse change downstream of the drug targets (Figure 2A). We identified a total of 32 CausalPath relations in the results that are verifiable by analyzing the existing inhibition experiments. We found that in 29 of the 32 cases the antibody readout changes in our predicted direction, and 3 of them change the other way, suggesting a precision of 0.91 without considering the significance of the change (Figure 2B and Table S1). Nineteen of those changes are statistically significant, with a 0.1 false discovery rate (FDR) cutoff; 18 of these are in the expected direction, validating the CausalPath result, and only one is contradicting. If we assume all insignificant cases are *unchanged*, then the precision estimate drops to 0.56. In reality, the insignificant results are a mix of changed and unchanged, because we chose a threshold to have a reasonably low false positive rate at the expense of a possible high false negative rate. To estimate the false negative rate, we assume that the noise is symmetrically distributed around 0 and assume all three cases on the negative side are noise, predicting three additional unchanged cases on the positive side. This brings the estimate of changed-in-expected-direction cases to 26, suggesting a more realistic precision estimate of 0.81. This level of precision is very reasonable for most studies to justify following up with experimental verification.

#### Analysis of PC3 cell line drug perturbations

To evaluate CausalPath on a series of perturbations systematically, we analyzed the mass spectrometry data from a recent set of drug perturbation experiments on PC3 prostate cancer cell lines where a total of 3,979 phosphopeptides were measured.^18^ For this analysis, we collected known targets of the drugs from the literature and inserted the inactivation of these targets as custom hypotheses. We found that for 14 of the 31 drugs CausalPath can identify proteomic changes that can be explained by inhibiting the drug's known target (Table S2). For four of these drugs, CausalPath detects enrichment downstream of the drug's targets, indicating its inactivation. In other words, even if we do not insert custom hypotheses for known drug targets, CausalPath can correctly predict the targets of these four drugs by evaluating changes in their downstream proteins. These drugs and their identified targets are afuresertib (AKT1), dinaciclib (CDK1, CDK2), flavopiridol (CDK1, CDK2, CDK6), and staurosporine (CDK2, MAPKAPK2) (Table S2). The results indicate that whenever a drug targets CDK1/CDK2 on PC3 cells (three of the drugs in the study), CausalPath can identify it using the downstream-size test. This implies that CDK1/2 activity is playing an important role in PC3 biology, a relatively large number of its targets respond to its inactivation, and also their relations are relatively well modeled in pathway databases.

### Analysis of the CPTAC ovarian cancer dataset

Four hundred eighty-nine high-grade serous ovarian cancer (HGSOC) samples were previously profiled by the TCGA project.^19^ A recent CPTAC project performed proteomic and phosphoproteomic analysis on 174 of the original TCGA ovarian cancer samples using mass spectrometry, providing measurements for 9,600 proteins from the 174 samples and 24,429 phosphosites from 6,769 phosphoproteins from 69 samples.^20^

**Figure 3:**
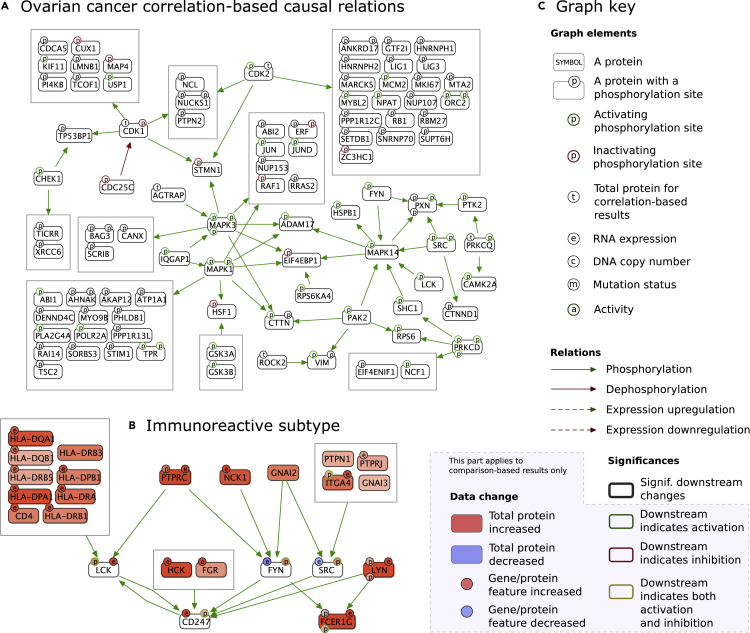
Results for CPTAC ovarian cancer (A) The largest connected component in the correlation-based causality network with phospho regulations. Note that the visual notation of this correlation-based result network is different from that of the comparison-based network in Figure 1, as we have no differential comparison but have pairwise correlations. For a compiled set of examples on how to read parts of a CausalPath result graph, please see Figure S4. (B) Immunoreactive subtype compared with all other samples, where we show RNA expression and DNA copy variation from corresponding TCGA datasets along with the CPTAC proteomic changes. (C) Key for the graph notation for causal explanations in all figures.

Using CausalPath on this dataset, we generated explanations for the observed correlations in the measured peptide levels, using phosphorylation and expression regulation pathway relations. The first case explains phosphopeptide changes through phospho relations, and the second case explains total protein changes through expression regulation relations. In both cases, the upstream “cause” in the explanations is either a total protein or a phosphoprotein change. The resulting phosphorylation network contains 139 relations and the expression network contains 243 relations when we use a 0.1 FDR threshold for correlations. Interestingly, while the size of the phosphorylation network is significantly large (p < 0.0001, calculated by data label randomization), we do not observe this for the expression network (p = 0.6283). The most notable parts of the phosphorylation network include CDK1 and CDK2 downstream, MAPK1 and MAPK3 downstream, and several immune-related proteins such as SRC family kinases, PRKCD, and PRKCQ (Figure 3A).

Potential reasons for the radically different significance values for expression-regulation relations compared with phosphorylations include lower quality of expression regulation priors, higher number of confounding factors, and the relatively weak correlation between total protein measurements and their corresponding RNA expression. To investigate this, we modified CausalPath to use TCGA RNA-sequencing (RNA-seq) data instead of proteomic data for the target genes of expression regulation controls. We obtained 192 expression regulations that explain RNA measurements of 140 genes with proteomic changes of 92 transcription factors or their modulators. The size of the resulting network became significant after this change (p < 0.0001), confirming that the proteomic change is not a very good proxy for RNA expression (and vice versa). In addition, the downstream changes in four transcription factors (STAT1, NFKB1, MCM6, and SPI1) are significantly large (0.1 FDR), suggesting that these factors are significant sources of variance in ovarian cancer.

The correlation-based causal network provides hypotheses for the signaling network parts that are differentially active across samples, but it does not indicate which parts are activated together or whether they align with previously defined molecular subtypes. The original TCGA study on HGSOC samples identifies four molecular subtypes based on RNA expression, termed as immunoreactive, differentiated, proliferative, and mesenchymal.^19^ To understand if we can gain mechanistic insight into the previously defined subtypes, we compared each subtype to all other samples using a t test with Benjamini-Hochberg FDR control on measurements, but we were unable to generate substantial results within a 0.1 FDR threshold, probably due to the large proportion of missing values in the phosphoproteomic dataset combined with the loss of statistical power due to smaller cohort size for each subtype. Then we tried to constrain the search space with the neighborhoods of some of the genes with differential measurements, and relax the FDR threshold at the same time for further exploration. Six SRC family kinases (SFKs) have proteomic evidence for activation in the immunoreactive subtype; hence, we limited the search to the neighborhood of SFKs (SRC, FYN, LYN, LCK, HCK, and FGR), set the FDR threshold to 0.2 for phosphoproteomic data, and identified 27 relations (Figure 3B). The network identifies several human leukocyte antigen system (the major histocompatibility complex in humans) proteins at SFK upstream, along with other genes regulating immune cell activation, such as CD4, ITGA4, PTPRC, PTPRJ, PTPN1, and NCK1. On the network, we identify a signal transmitted from SFKs to CD247 and FCER1G, immune response genes.

### Analysis of the CPTAC breast cancer dataset

**Figure 4:**
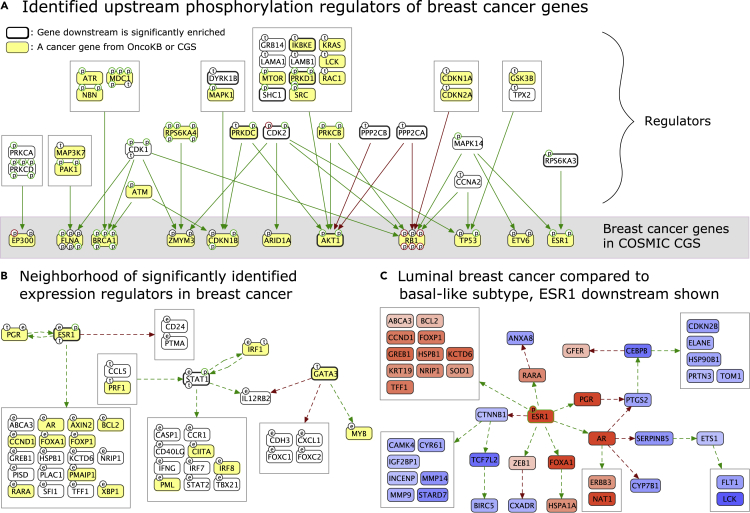
Results for CPTAC breast cancer (A) A subgraph of the correlation-based causal network with phospho regulations focused on the upstream regulators of proteins that are implicated in breast cancer as provided by the COSMIC Cancer Gene Census (CGS) database. There are 43 genes in CGS annotated with breast cancer, for 11 of which we identify phosphorylation regulators. (B) Subgraph of the correlation-based causal network with expression regulations where RNA-seq changes are explained by upstream proteomic changes, focused on the neighborhood of proteins with significant downstream. (C) Luminal A and luminal B subtypes are collectively compared with the basal-like subtype. Only the ESR1 downstream relations are shown.

Another CPTAC project produced proteomic and phosphoproteomic profiles for 105 of the original TCGA breast cancer samples with mass spectrometry,^21^ where 77 of the samples were tagged by the authors as being high quality and were used in this study. Unlike the ovarian cancer dataset, this dataset is rich in correlations, which can be explained by 1,756 phospho regulations and 488 expression regulations. The resulting phosphorylation network has 11 significant proteins (PRKD1, CDK2, DYRK1B, PPP2CA, MAPKAPK2, PPP2CB, RPS6KA3, PRKDC, AKT1, SHC1, and IKBKE) with an enriched downstream (Figure 4A). These enriched proteins all have established functions in breast cancer literature—maybe with the exception of DYRK1B, whose high expression was only recently associated with worse prognosis in breast cancer,^22^ potentially because its inhibitory effect on the cell cycle rescues breast cancer cells from apoptosis and cytotoxic drugs.^23^

Similar to the ovarian cancer results, we detected that the breast cancer phosphorylation network is significant in size (p < 0.0001), while the expression network is not (p = 0.5521), suggesting that the known phosphorylation relations have a much higher impact on the proteomic correlations than known expressional relations. When we use TCGA RNA-seq data instead of proteomic data for the targets of expression regulation, we detect 248 relations that explain RNA changes in 155 target genes by proteomic changes of 120 transcription factors or their modulators. With RNA-seq data, the size of the network is highly significant (p < 0.0001), with 3 transcription factors (GATA3, STAT1, and ESR1) having correlated targets enriched in the results (Figure 4B).

Next, we compared the PAM50 expression subtypes of breast cancer to see if we could get causal explanations of their proteomic differences. We were again challenged by decreased sample sizes and missing values, but we detected that luminal A and luminal B subtypes have significant differences from the basal-like subtype. This time, CausalPath results were not significant in terms of the overall network size (p = 0.2218); nevertheless, they indicate that ESR1 is significantly more active in luminal breast cancers, suggested by both its protein levels and the changes in its downstream (Figure 4C). Transcriptional downstream of ESR1 captures other important elevated transcription factors functioning in the luminal subtypes, such as FOXA1, AR, and PGR. AR is an emerging target in breast cancer.^24^

### Analysis of TCGA RPPA datasets

**Figure 5:**
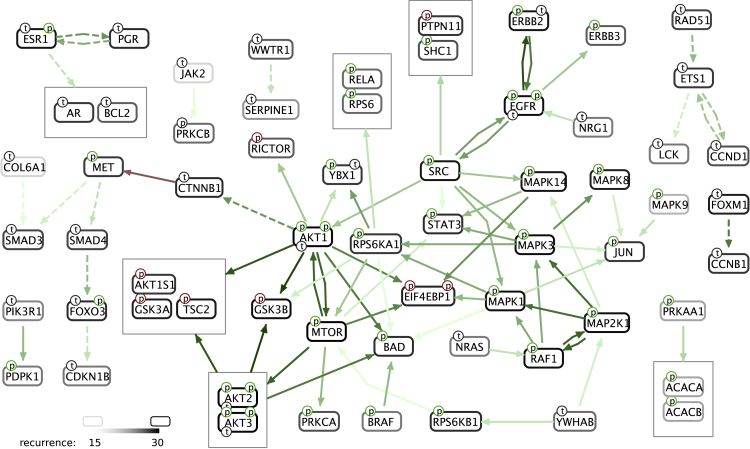
Recurrent results for TCGA RPPA datasets Relations that are identified with correlation-based analysis in at least 15 cancer types are shown, where faintest color indicates 15 and boldest color indicates 30. Please note that the bold node borders are repurposed in the graph notation to display recurrence rate.

There are 32 TCGA studies that provide proteomic and phosphoproteomic measurements of tumor biopsies from various types of cancer patients. Those studies provide RPPA profiles of a total of 7,694 patients using 259 antibodies. The low number of protein measurements in the datasets prevents a comprehensive pathway analysis, but the antibodies are selected for the proteins’ relevancy to cancer in general, and they are typically well studied with many established relations between them. We sought to determine which of these relations most frequently have evidence in the form of correlation across cancer types. We generated a correlation-based causal network for each cancer type using a strict FDR threshold of 0.001, then we ranked these relations according to how many cancer datasets they can explain (Figure 5 and Table S3). We found that AKT to GSK3 signaling is the most frequently observed relation, detectable in 30 cancer types, followed by other downstream proteins of AKT, including MTOR. Relations between several MAPK signaling proteins and EGFR to ERBB2 signaling are also among those observed in the vast majority of cancer types. It is important to note that the results do not indicate that these signaling paths are almost always active in cancers, but they indicate that there is a high patient-to-patient variation in their activity, almost always, making them relevant for precision medicine. This is consistent with many studies reporting the AKT pathway as a major resistance mechanism to chemotherapy and some other targeted therapies.^25^, ^26^, ^27^

## Discussion

### Pathway extraction versus pathway inference

CausalPath is a novel pathway extraction method to aid researchers in understanding experimental observations using known mechanisms with a focus on post-translational modifications. Experimental data reveal protein features that change in coordination, and CausalPath automates the search for causal explanations in the literature. Loosely speaking, context-specific correlations are derived from the data and causality is derived from the literature. Compared with the methods that infer causality from data through mathematical modeling (pathway inference), this method has a much wider application area. Pathway inference methods have a potential to offer more complete results, but they require numerous perturbations and/or time points in the experiments, whereas the pathway extraction strategy is applicable to any simple comparison, or a set of profiles from a cohort with some variance to explain. This is extremely important, since even with the large CPTAC datasets, we are still data limited, especially when we try to understand subtype-specific mechanisms. We believe that we will see parallel progress in both strategies as the data and knowledge increase respectively with a potential convergence in the future. In addition, CausalPath is a great resource for high-confidence *priors*, which can inform the hybrid pathway inference methods that benefit from prior data.

### Novelty in pathway extraction

Even though pathway extraction cannot hypothesize the existence of new relations that were never seen before in any context, its results cannot be dismissed as not novel. Existing relations in pathway databases are collected from diverse contexts, cell types, disease models, etc. For a new context of focus, it is very challenging to identify which of the previously described relations are applicable. Causality-focused pathway extraction approaches provide a means of transferring knowledge between contexts. CausalPath does this by detecting variation patterns of proteomic abundances and detecting their consistency with prior knowledge. A limitation of this approach is its dependency on observable variance; therefore, it cannot identify a signaling relation that does not significantly vary across the compared samples.

#### The added value and future challenges

The added value that our method brings to the field of pathway extraction is three-fold: (1) interpretation of complex mechanistic pathway models, (2) site-specific evaluation of phosphoproteomic measurements, and (3) a logical test for causality between measurements. When all these are combined, pathway extraction becomes a useful tool for “mechanistic model building.” We expect future research will take these ideas further, potentially addressing these two challenges: (1) instead of a binary evaluation of causality (between two proteins), n-ary systems can be developed, and (2) instead of a binary classification of protein modifications as activating and inhibiting, a site can be more accurately mapped to a distinct subset of activities of the protein. These challenges can be tackled gradually as we have more detailed and more complete models of cellular processes in pathway databases.

### Proteomic versus transcriptomic level events

Our results show that evidence of known phospho regulations is more consistently observed in the proteomic data compared with the known expression regulations. In the ovarian and breast cancer datasets, the sizes of the resulting phosphorylation networks are significantly higher compared with background, while expression networks remain similar. In the recurrence study with TCGA RPPA datasets, 34% of the resulting phosphorylation controls recur in at least 15 cancer types (Figure S2). This ratio is only 7% for expression level controls. This is perhaps expected, as a phosphorylation relation can directly explain a phosphopeptide change, while an expression event can only indirectly explain a total protein change requiring the mRNA level of the target to be highly correlated with its protein abundance. While there is definitely an overall correlation (Figure S3), it is not high enough to use protein data as a reliable proxy for mRNA in general. However, there are exceptions, for example, we could identify ESR1 differential activity in luminal breast cancers purely from proteomic data, using expression relations. Based on these observations, CausalPath lets users select the molecular data type to use for targets of expression relations.

### Missing or flawed pathway relations

One major limiting factor in this analysis is a large number of protein phosphorylation sites whose functions are not known; hence, their downstream cannot be included in the causality network. We are actively working to mine these data from the literature using natural language processing tools.^28^ In the meantime, CausalPath reports those sites with an unknown effect that also have significant change at their signaling downstream. Users have the option to review this list of modification sites and manually curate them to increase the coverage of the analysis.

Rarely, in the causality analysis results, we encounter relations that are erroneous. These are generally results of manual curation issues. In these cases, we report them to the source databases, and we remove these erroneous pathway interactions from our network so that future analyses are not affected. We encourage researchers to report such errors to source databases (or alternatively to us), if they come across any, to improve the accuracy of our collective knowledge of biochemical pathways. We are actively working on a collaborative data-explaining platform that will further streamline the curation and error reporting steps.

### Recommendation for use

CausalPath can be applied to the results of any proteomic and phosphoproteomic experiments to identify differential signaling that is supported by literature knowledge. To use CausalPath, the measurement values need to be comparable (normalized) and need to be associated with related gene symbols, and phosphopeptide measurements need to specify the phosphorylation sites with respect to their canonical UniProt sequence, in a special format that is described at the website, causalpath.org. Users can either use the website to execute the analysis or run the analysis locally using CausalPath's open-source Java code. The result networks can be visualized using the viewer embedded in the CausalPath website or by loading to the pathway visualization tools ChiBE^29^^,^^30^ or Newt.^31^

## Experimental procedures

### Resource availability

#### Lead contact

Further information and requests for digital resources should be directed to and will be fulfilled by the lead contact, Özgün Babur (ozgun.babur@umb.edu).

#### Materials availability

This study did not generate new unique reagents.

#### Data and code availability

CausalPath is freely available at http://causalpath.org. Users can upload the proteomic data in a tab-delimited format, along with the analysis parameters, such as how to detect a *change* in the values. Options include averaging a group of values, getting difference/fold-change of two groups of columns, comparing two groups with a t test, or using correlations in a single group. The results are visualized as an interactive network using Cytoscape.js,^32^ and the mechanistic details of each interaction can be viewed in SBGN-PD language^31^ using a layout algorithm specifically designed for compound graph structures.^33^ Alternatively, CausalPath can be run locally as a Java application using the sources at https://github.com/PathwayAndDataAnalysis/causalpath. This repository additionally includes examples running CausalPath from R and Python.^34^ The generated result networks can be visualized with ChiBE,^29^^,^^30^ as well as by uploading analysis output folders to the CausalPath web server at causalpath.org. All network figures in this article were generated with ChiBE.

The results presented in this article can be reproduced using the datasets, parameters, and software in the supplementary archive at https://www.synapse.org/#!Synapse:syn17014378. An alternative URL for this archive is https://doi.org/10.5281/zenodo.4477801.

## Supplemental information

Document S1. Supplemental experimental procedures, Figures S1–S21, and Data S1

Table S1. All the tested cases where an inhibitor drug targets a protein that was predicted to be activated by CausalPath under a ligand stimulation Highlighted p values are significant ones with FDR 0.1. Normalized mean change is positive if the change is in the expected direction, and negative otherwise

Table S2. Summary of the results of analysis of drug perturbations in PC3 cell lines Each perturbation analysis result is evaluated for if any phosphoproteomic change could be causally linked to inhibition of a known target, and if any known target is identifiable solely from the phosphoproteomic changes. Successfully predicted known targets are underlined.

Table S3. Inferred relations and their distribution to the studies in TCGA RPPA datasets The relations are ordered by their detection recurrence in the studies. The studies are ordered by the number of result relations they produce.

Document S2. Article plus supplemental information
