# Supplementary material for: Causal interactions from proteomic profiles: Molecular data meet pathway knowledge
Source: Patterns (N Y). 2021 May 12;2(6):100257. doi: 10.1016/j.patter.2021.100257 (PMC8212145; doi:10.1016/j.patter.2021.100257)
Supplement: Document S1. Supplemental experimental procedures, Figures S1–S21, and Data S1 [file mmc1.pdf]

Patterns, Volume 2

Supplemental information

Causal interactions from proteomic profiles:

Molecular data meet pathway knowledge

Özgün Babur, Augustin Luna, Anil Korkut, Funda Durupinar, Metin Can Siper, Ugur Dogrusoz, Alvaro Sebastian Vaca Jacome, Ryan Peckner, Karen E. Christianson, Jacob D. Jaffe, Paul T. Spellman, Joseph E. Aslan, Chris Sander, and Emek Demir

Supplemental Information

Contents

1 Supplemental Experimental Procedures **2**
1.1 Significance for proteomic data change and correlation . . . 2
1.2 Causality . . . 2
1.3 Derivation of prior relations from detailed mechanistic pathways . . . 2
1.4 Algorithm for selection of explanatory subset of prior relations . . . 2
1.5 Description of causal graph notation . . . 3
1.6 CausalPath parameters . . . 4
1.6.1 Site matching proximity threshold . . . 4
1.6.2 Site effect proximity threshold . . . 4
1.6.3 Gene focus . . . 4
1.6.4 Generation of a data-centric causal network . . . 4
1.6.5 Protein activity . . . 5
1.6.6 Adjusting phosphopeptide measurements with total protein . . . 5
1.6.7 Data type for expressional targets . . . 5
1.6.8 Using custom resources . . . 5

2 Supplementary Figures **5**

3 Data S1 **11**
3.1 Robustness . . . 11
3.2 Reproducibility . . . 11
3.3 Perturbation studies with short or long readout times . . . 11
3.4 Evaluation of effect sizes in all analyses . . . 12
3.5 Previously published methods for pathway analysis of proteomic datasets . . . 13
3.5.1 Temporal Pathway Synthesizer (TPS) . . . 13
3.5.2 PARADIGM . . . 16
3.5.3 pCHIPS . . . 16
3.5.4 SigNetTrainer . . . 17
3.5.5 PHONEMeS . . . 17
3.5.6 Method from Chasman *et al.* . . . 17
3.5.7 PhosphoPath and PTMapper . . . 17
3.5.8 PCST . . . 17
3.5.9 PHOTON . . . 17
3.6 Library of graphical patterns used for causality detection . . . 18
3.7 Extending graphical patterns . . . 31

1 Supplemental Experimental Procedures

1.1 Significance for proteomic data change and correlation

For comparison-based analyses we used a two-tailed t-test for calculating the significance of the difference of the means of the two groups in the comparison, requiring the presence of at least 3 non-missing values from all compared groups. For correlation-based analyses we used the Pearson correlation coefficient and its associated significance, requiring at least 5 samples in the calculation. Both tests assume a null model where molecular readouts change independently. The EGF stimulation dataset provides pre-calculated p-values for all of the pairs of time points [1], which we directly used in the analysis. For the analysis of the cell line dataset from Hill *et al.*, we used the "complete" version of their dataset and did not do any sample or antibody filtering. We used a paired two tailed t-test to compare readouts at multiple time points collectively. In all calculations, we used the Benjamini-Hochberg (BH) method for controlling false discovery rate (FDR), whenever applicable. Prior to the application of the BH method, the software detects data rows that have potential to become a part of a causal hypothesis, without considering their significance or direction, and applies the BH method to only these rows. This step filters out data rows whose significance or direction will never be evaluated during any causal reasoning, and prevents over-correction. We used 0.1 as a default FDR threshold unless indicated otherwise.

1.2 Causality

Both pathway inference and pathway extraction are closely related to formal notions of causality inference, specifically to the Suppes' and Pearl's probabilistic formulations. A probabilistic causal relationship between two events, say from event A to event B, indicates the probability that B depends on the status of A, as described by Patrick Suppes [2]. While using this notion of causality can generate predictive models, it does not tell if A may cause B. For instance, there can be an event X that is causing both A and B, and this will still satisfy Suppes' formulation. To make the model predictive under an intervention scenario, Judea Pearl provided a reformulation: perturbing the status of A will change the probability of B [3]. We follow Pearl's notion and detect mechanism-based evidence for activity change of one protein may affect the abundance of a specific peptide from another protein in pathway databases, as described in the next section and in Supplementary Information.

1.3 Derivation of prior relations from detailed mechanistic pathways

Using the BioPAX-pattern framework, and by studying the structure of the BioPAX models from different resources, we manually defined 12 BioPAX patterns to capture potentially causal binary relations that involve phosphorylation and expression of proteins. We provide the details of these patterns in Supplementary Information, along with examples for what they can detect. The source code of the software that we used for extracting causal priors can be found at https://github.com/PathwayAndDataAnalysis/causal-priors-extractor. We applied the version 1.0.0 of this code on Pathway Commons v9 to generate a part of the causal priors that we used in this study.

1.4 Algorithm for selection of explanatory subset of prior relations

Using the extracted causal priors, CausalPath determines if there is sufficient proteomic data that indicates differential activity of that prior. The pseudocodes below implement the logical equations that check conditions of causality.

Comparison-based detection (algorithm that tests Eq. 1):

```
For each prior relation pr
    s ← pr.source
    t ← pr.target
    if pr is phosphorylation or dephosphorylation
        for each phosphopeptide measurement mpt on matching sites of t
            for each total protein measurement mts on s
                if mts.sign * pr.sign * mpt.sign = 1
                    add pr to results
            for each phosphopeptide measurement mps on s
                if mps.effect * mps.sign * pr.sign * mpt.sign = 1
                    add pr to results
    else if pr is upregulation or downregulation of expression
        for each total protein measurement mtt on t
            for each total protein measurement mts on s
                if mts.sign * pr.sign * mtt.sign = 1
                    add pr to results
            for each phosphopeptide measurement mps on s
                if mps.effect * mps.sign * pr.sign * mtt.sign = 1
                    add pr to results
```

Correlation-based detection (algorithm that tests Eq. 2):

```
For each prior relation pr
    s ← pr.source
    t ← pr.target
    if pr is phosphorylation or dephosphorylation
        for each correlated s tot-prot and site-matching t phosphoprot
            if corr.sign * pr.sign = 1
                add pr to results
        for each correlated s phosphoprot (mps) and site-matched t phosphoprot
            if mps.effect * corr.sign * pr.sign = 1
                add pr to results
    else if pr is upregulation or downregulation of expression
        for each correlated s tot-prot and t tot-prot (mtt)
            if corr.sign * pr.sign = 1
                add pr to results
        for each correlated s phosphoprot (mps) and t tot-prot (mtt)
            if mps.effect * corr.sign * pr.sign = 1
                add pr to results
```

These algorithms use *.sign* and *.effect* properties of variables that takes values -1, 0 and 1, corresponding to *false*, *unknown* and *true*, respectively. Multiplication of these integer values and checking the result value is an alternative formulation to the original logical equations where ternary XOR ($\oplus$) operator is used. When the RNAseq data is used at the targets of expressional control relations, the total protein measurements (*mtt* in the pseudocode) are replaced with the RNAseq measurements of the target (*mrt*).

1.5 Description of causal graph notation

We developed a new graph notation to represent resulting causal explanations as a logical network, where nodes denote proteins and edges denote causal relations (Fig. 3c). Node background is used for color-coding total protein change, while site-specific changes are shown with small circles on the nodes whose border colors indicate whether the site is activating/inhibiting. If additional omic data such as RNA expression, DNA copy number, or mutation status are available, we include them for integrated visualization, using small circles displaying specialized letters. Binary causal relations are represented with edges—green representing

positive, red representing negative, phosphorylations with solid edges and transcriptional regulations with dashed edges. When significance calculation results are available, they are represented on the protein borders, using a bold border when downstream of a protein is significantly large, green border when downstream indicates the protein is activated, and red border when downstream indicates the protein is inactivated. If both the activation-indicating and inhibition-indicating downstream relations are significantly large, then a dark yellow color is used instead. We use topology grouping while rendering result networks, which means we group the proteins with the same network topology under compound nodes on the network for complexity management. To further clarify the graph notation, we provide examples in Supplementary Figure S4.

1.6 CausalPath parameters

CausalPath is designed to explore omic datasets of different sizes, types, and accuracy. Following are some important parameters to consider when using the method.

1.6.1 Site matching proximity threshold

Protein phosphorylation sites in the literature have to exactly match the detected site in the phosphoproteomic dataset to use in causal reasoning by default. Some users may find this too strict since there can be slight shifts in the literature, or some nearby sites of proteins are likely to be phosphorylated by the same kinase. This parameter makes the analysis allow a determined inaccuracy in site mapping to explore such cases. Increasing this parameter will increase the result network size by allowing proximate site matching (Suppl. Fig. S5, blue bars). This parameter can be used to increase to coverage of the results, however, the new relations in the results are likely to have more false positives than other relations. In this manuscript, we used strict site-matching for all the CausalPath analyses unless indicated otherwise.

1.6.2 Site effect proximity threshold

The effect of the phosphorylation sites on the protein activity, as in activating or inactivating, is curated by pathway databases, mostly by PhosphoSitePlus. We also did some small-scale curation for EGF stimulation analysis, and RPPA analyses. CausalPath requires exact matching of these curated site effects with the sites in the data by default, however, this can be too strict because nearby sites generally tend to have similar effects. This parameter lets the analysis use a determined inaccuracy while looking up site effects. Increasing this parameter will increase the result network size by reducing the portion of phosphorylation sites with unknown effect (Suppl. Fig. S5, red bars). Similar to the previous parameter, this parameter increases coverage at the cost of increase in false positives. In this manuscript, we always used accurate matching for site effects.

1.6.3 Gene focus

This parameter lets the analysis use a subset of the literature relations, focusing on the neighborhood of certain proteins indicated by their gene symbols, hence reducing the number of tested hypotheses. We used this parameter during the analysis of ovarian cancer subtypes as described in the relevant section. Gene focus may be useful in two ways: (i) removes irrelevant parts of the prior relations, providing a means of complexity management, (ii) may increase statistical power for differential abundance detection by decreasing the total number of tested peptides.

1.6.4 Generation of a data-centric causal network

CausalPath result networks are gene-centric, meaning that genes are represented with nodes, and all other measurements related to a gene are mapped on the gene's node. When a data row can map to multiple genes, however, this creates a redundancy, as we have in the RPPA analysis results. For example, the AKT phosphoantibody can recognize all three AKTs, so we duplicated the same data on AKT1, AKT2, and AKT3. A similar problem exists for mass spectroscopy when a phosphopeptide can be resolved to multiple homologous proteins, if their sequences are identical around the phosphorylation site, AKTs again being an example. As an alternative to this view, CausalPath can generate data-centric views where nodes represent

data rows unresolved to particular proteins. But this view does not support mapping other available -omics data onto the network, and also the relations are duplicated when more than one data of the same gene can be explained by the same relation.

1.6.5 Protein activity

CausalPath allows users to insert their own hypotheses as to whether a protein is activated or inhibited in the case of a comparison-based analysis. The input has to be a gene symbol associated with a Boolean parameter indicating the hypothesized direction of activity change. We used this option for EGF stimulation analysis to indicate that we expect EGF to be activated because the data only measures phosphoprotein abundances, hence there is no measurable change on EGF itself to include in the analysis otherwise.

1.6.6 Adjusting phosphopeptide measurements with total protein

When both phosphoprotein and total protein measurements are available in a study, an optional adjustment can be done to the phosphopeptide values to reflect their relative abundance to the total protein, before applying CausalPath. Analyses in this manuscript do not include this type of adjustment. Each protein feature is independently used in causality checks.

1.6.7 Data type for expressional targets

CausalPath restricts its logical reasoning within proteomic data by default, however, users can opt to use mRNA data for the targets of expressional relations. We used this parameter during the correlation-based analysis of ovarian and breast cancers as described in the results section. It is also possible to use mRNA and protein data together by using this parameter multiple times.

1.6.8 Using custom resources

CausalPath resources are embedded in its code base by default, and they are subject to change with new versions of the software. To establish reproducibility and customizability, CausalPath allows users to override the following resources: (i) list of priors relations using the “custom-prior-relations-file” parameter, (ii) list of known site effects using the “custom-site-effects-file” parameter, (iii) list of recognized HGNC symbols using the “hgnc-file” parameter. For this manuscript, we provide these resources in a zip archive, to ensure reproducibility and to provide example for resource customization.

2 Supplementary Figures

	Evidence for protein A *activity* change			
types of information chained to form a causal conjecture	Experimental evidence for protein A *feature* change	Prioir knowledge of protein A feature affects protein A activity	Prior knowledge of protein A activity controls protein B feature	Experimental evidence for protein B *feature* change
possible values	phosphopeptide increase<br>phosphopeptide decrease<br>total protein increase<br>total protein decrease	activating phosphorylation site<br>inhibiting phosphorylation site<br>*by default: total protein level positively affects its activity*	phosphorylation<br>dephosphorylation<br>expression upregulation<br>expression downregulation	phosphopeptide increase<br>phosphopeptide decrease<br>total protein increase<br>total protein decrease
all valid combinations	phosphopeptide increase	activating phosphorylation site	phosphorylation	phosphopeptide increase
	phosphopeptide decrease	inhibiting phosphorylation site	phosphorylation	phosphopeptide increase
	phosphopeptide increase	activating phosphorylation site	dephosphorylation	phosphopeptide decrease
	phosphopeptide decrease	inhibiting phosphorylation site	dephosphorylation	phosphopeptide decrease
	phosphopeptide increase	inhibiting phosphorylation site	phosphorylation	phosphopeptide decrease
	phosphopeptide decrease	activating phosphorylation site	phosphorylation	phosphopeptide decrease
	phosphopeptide increase	inhibiting phosphorylation site	dephosphorylation	phosphopeptide increase
	phosphopeptide decrease	activating phosphorylation site	dephosphorylation	phosphopeptide increase
	total protein increase		phosphorylation	phosphopeptide increase
	total protein decrease		phosphorylation	phosphopeptide decrease
	total protein increase		dephosphorylation	phosphopeptide decrease
	total protein decrease		dephosphorylation	phosphopeptide increase
	phosphopeptide increase	activating phosphorylation site	expression upregulation	total protein increase
	phosphopeptide decrease	inhibiting phosphorylation site	expression upregulation	total protein increase
	phosphopeptide increase	activating phosphorylation site	expression downregulation	total protein decrease
	phosphopeptide decrease	inhibiting phosphorylation site	expression downregulation	total protein decrease
	phosphopeptide increase	inhibiting phosphorylation site	expression upregulation	total protein decrease
	phosphopeptide decrease	activating phosphorylation site	expression upregulation	total protein decrease
	phosphopeptide increase	inhibiting phosphorylation site	expression downregulation	total protein increase
	phosphopeptide decrease	activating phosphorylation site	expression downregulation	total protein increase
	total protein increase		expression upregulation	total protein increase
	total protein decrease		expression upregulation	total protein decrease
	total protein increase		expression downregulation	total protein decrease
	total protein decrease		expression downregulation	total protein increase

graphical representation

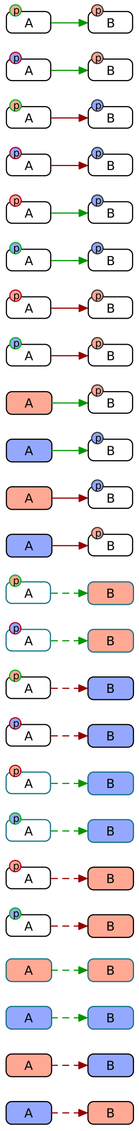

Figure S1: Causal conjecture generation for comparison-based analysis (Step 2 of CausalPath workflow). CausalPath integrates 4 types of information such that the prior information forms a causal bridge between a pair of observed proteomic changes. Each type of information has a fixed number of possible values. Valid combinations of these values are detected using Eq. 1, also iterated here.

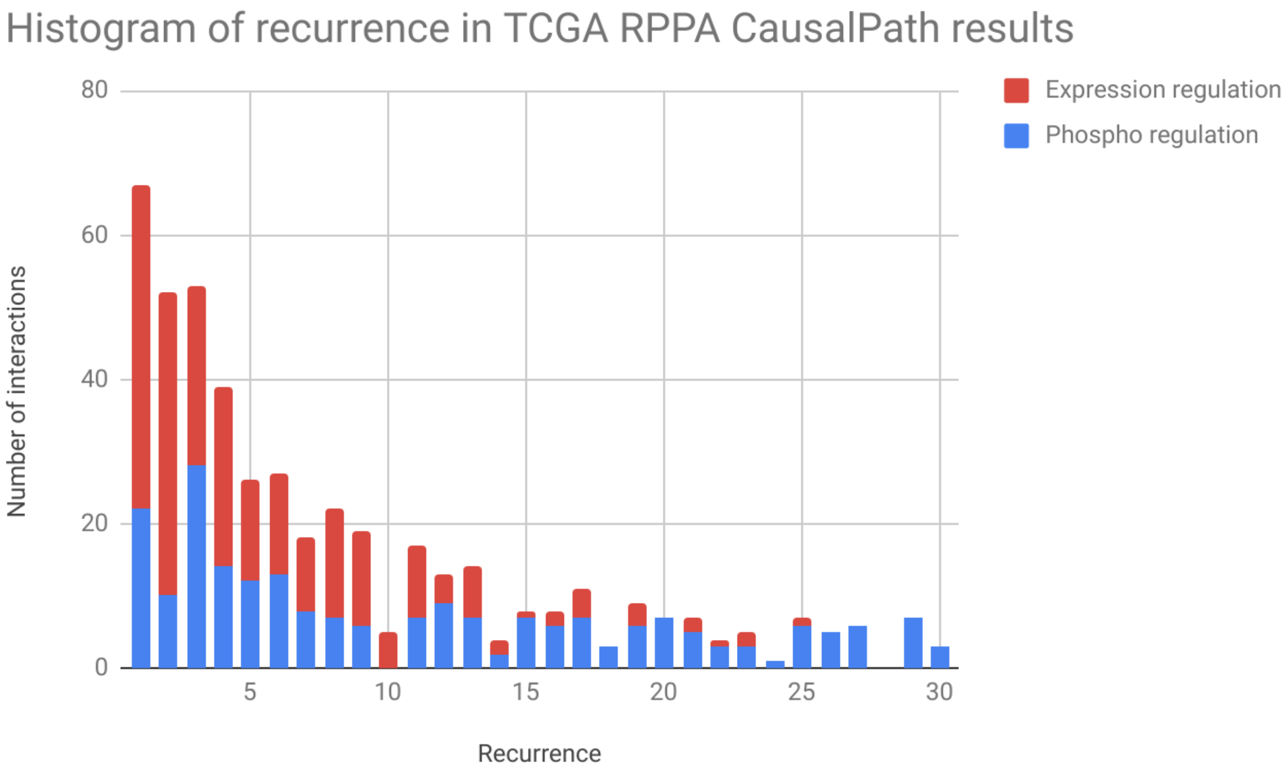

Figure S2: Recurrence histogram for the relations in the results from CausalPath analysis of TCGA RPPA datasets. The histogram has a significant long tail showing a subset of the results are highly recurrent. While the total number of expression regulations and phospho regulations are similar, the high-recurrence tail is dominated by phospho regulations.

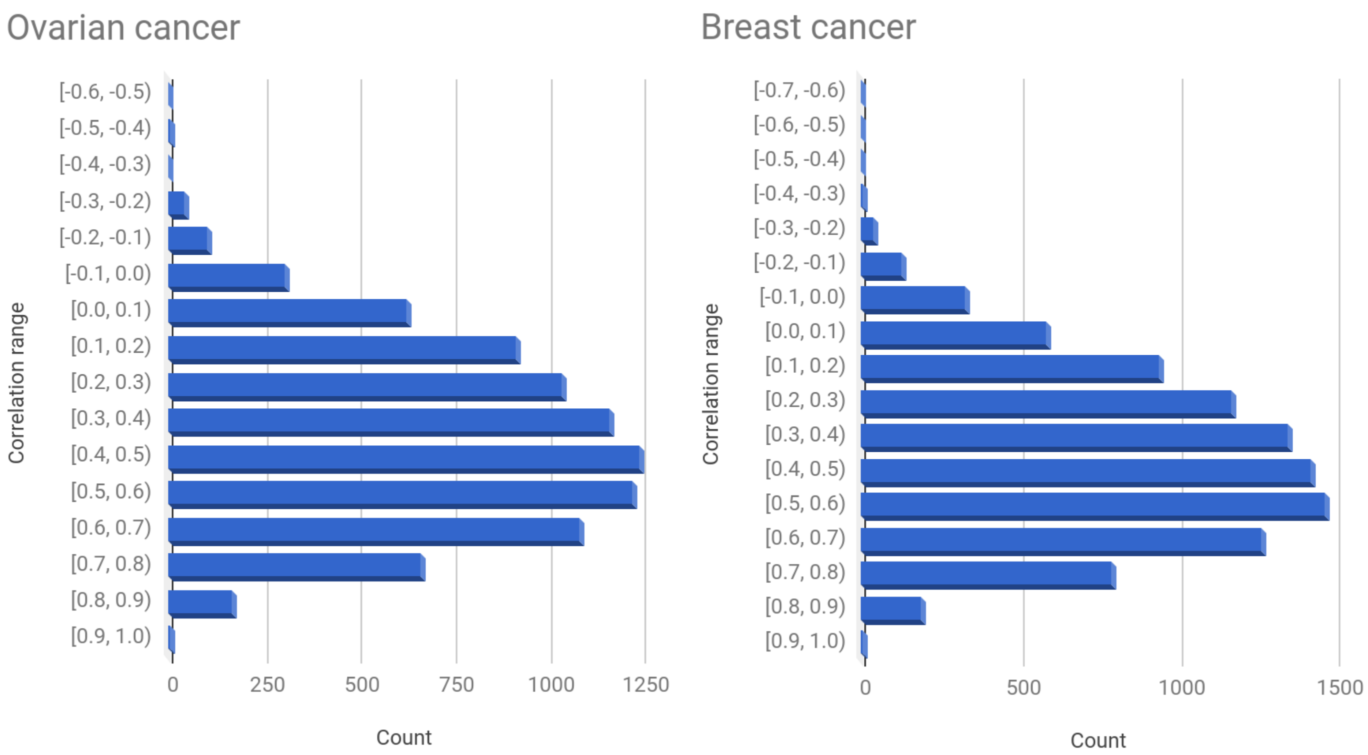

Figure S3: Histograms for the distribution of correlations between mRNA and protein expressions of genes in ovarian and breast cancer datasets. Both datasets have similar distributions, peaking around 0.5 Pearson correlation.

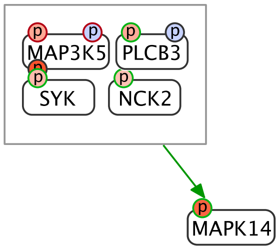

4 proteins are grouped together as possible cause for MAPK14 phosphorylation. This grouping does not imply that they form a complex, but they are grouped only based on their topology in this graph, so that the phosphorylation relation is not drawn 4 times. Each of the 4 proteins can have a different mechanism for phosphorylating MAPK14. MAP3K5 has 3 site-specific measurements on it, two of them are inactivating (red border), and one has unknown effect (black border). Among inactivating, one goes up and one goes down. Here the causal hypothesis is constructed with the site that goes down. The other site change is in conflict with this hypothesis but shown anyway for completeness. Even though the arrow seems to be pointing at "p", this is only a coincidence as CausalPath graphs do not show edge-to-feature mapping, but they may be logically inferred.

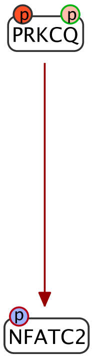

NFATC2 is activated by dephosporylation of its inactivating site. This dephosphorylation can be due to PRKCQ phosphorylation and activation. This relation is unintuitive because PRKCQ is a kinase and not a phosphatase. The causal link from PRKCQ to NFATC2 is a relatively complex sequence of events (right), involves FLNA/PRKCQ complex increasing the intracellular calcium levels, and the calcium inhibiting the inhibitory phosphorylations on NFATC2. This example demonstrates that it is important to not to assume simple phosphorylation/dephosphorylation when reading CausalPath graphs as they can be more complex indirect mechanisms.

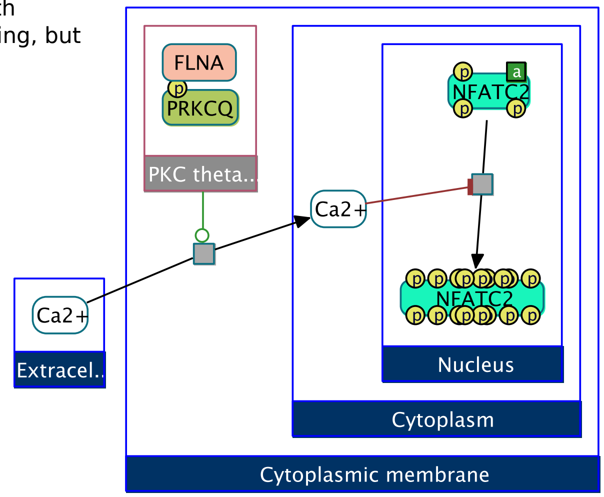

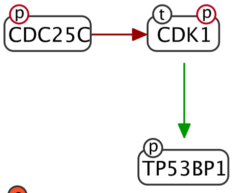

A correlation-based graph of 3 proteins. CDK1 has 2 measured features: total protein (t) and phosphorptein (p). The graph does not show which relation is related to which feature, but in this case, it is inferrable. The target of the dephosphorylation (red edge) has to be the "p" because we do not expect a total protein change with dephosphorylation. As a result, this graph indicates presence of a positive correlation between CDC25C-p and CDK1-p. The source of the phosphorylation (green) relation can either be "t" or "p" as both can be a proxy for the activity of CDK1.

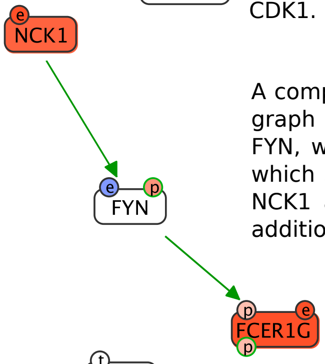

A comparison-based graph where RNA expression changes are also displayed on the graph (e). The graph shows that NCK1 total protein increase may have caused phosphorylation and activation of FYN, which in turn may have caused phosphorylation of FCER1G. The graph does not distinguish which site of FCER1G is phosphorylated by FYN, and can be both. We see that RNA expressions of NCK1 and FCER1G are increased, and RNA expression of FYN is decreased, but these are only additional information and were not used in causal reasoning.

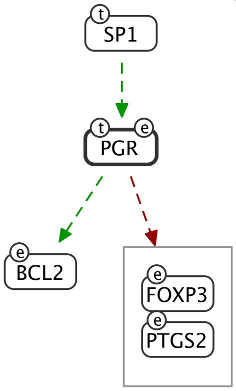

A subgraph from results of a correlation-based analysis where RNA expression data is used at the targets of expression regulations for causal reasoning. SP1 total protein is positively correlated with PGR RNA expression. PGR total protein is positively correlated with RNA expression of its positive target BCL2, and it is negatively correlated with RNA expressions of its negative targets FOXP3 and PTGS2. The sign of these correaltions are not shown on the graph, but we infer them from the nature of their relationships. PGR has a bold border indicating its downstream is significantly large on the network. This can be unintuitive since 3 is not a large number. There can be two reasons for a significance with a few downstream targets: (i) the number of targets in the base network (causal priors) may be a low number, which makes 3 significantly large, (ii) there may not be many RNA expressions correlated with PGR total protein overall, which again makes 3 significantly large.

Figure S4: A selection of examples from CausalPath results (left) and their textual explanation (right).

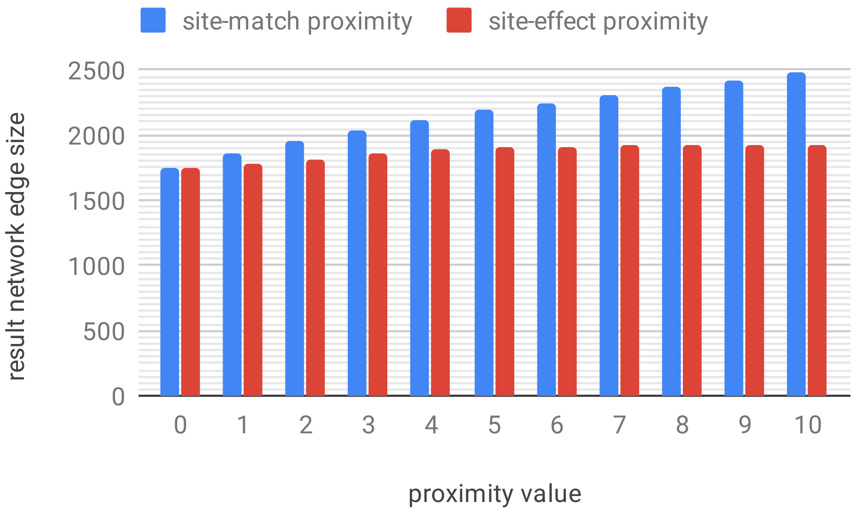

Figure S5: The effect of the parameters "site-match-proximity-threhsold" and "site-effect-proximity-threshold" on the result size of the correlation-based analysis of CPTAC breast cancer dataset using phospho-relations. Increasing the first parameter makes an higher impact on the result size than increasing the second parameter.

3 Data S1

3.1 Robustness

We tested the robustness of CausalPath against noise on the CPTAC breast cancer dataset, on a correlation-based analysis with phosphorylation relations using 0.1 FDR cutoff. We iteratively and cumulatively introduced noise into the dataset by adding random numbers to the proteomic data, drawn from a normal distribution, and generated the causal network. At each step, we recorded the amount of the overlap with the original relations (Fig. S6a). Ideally, as the noise level increases, we would like the method to retain the original results and not allow the noise to generate non-overlapping new relations. Our tests indicate that CausalPath is sensitive to noise, which means overlapping relations rapidly start decreasing in number, however, the method is safe against noise, which means we do not get many non-overlapping relations due to it. This means that the data quality highly affects the size of the results, but it is not risky to try CausalPath on high-noise datasets.

We next assessed the importance of the quality of causal priors on the quality of the results using the same dataset with data label randomizations. Randomization of data labels means random assignment of data rows to the prior network. The chance of a network relation to get accidentally selected into the results after randomization is roughly equal to a random false relation to accidentally pair with compatible data and to get selected into the results. After data label randomization, the correlation-based analysis of CPTAC breast cancer dataset on phosphorylation controls produce an average of 1,127 relations (with a standard deviation of 88) out of 27,196 prior relations. This means a random incorrect causal prior has about 4% chance to generate noise in the results. This is low enough for the method to tolerate occasional inaccurate priors, but the method definitely depends on high-quality of the causal priors to generate useful results. Based on this observation, we used only manually-curated high-quality prior data in our analyses.

3.2 Reproducibility

We tested the reproducibility of CausalPath on the CPTAC breast cancer dataset by using random subsets of the samples, on a correlation-based analysis with phosphorylation relations using 0.1 FDR cutoff. In a total of 100 trials, we used a random half of the samples at each step and checked how frequently each causal relation is reproduced in the results and checked their overlap with the original results where all of the samples were used (Fig. S6b,c). The results indicate that half of the overlapping relations are reproduced in at least 61% of the trials consistently (yellow mark in Fig. S6c), while 11% are reproduced in all of the trials (last bar in Fig. S6b). The significant amount of non-overlapping relations (red) with low reproduction counts in the tests are due to the accumulation of false positives from all 100 trials.

3.3 Perturbation studies with short or long readout times

For the analysis of a perturbation study with time resolution, CausalPath may not be equally successful to explain each time point. Early time points (minutes to hours) are shaped by short range, direct effects and late time points (hours to days) mostly reflect long range, indirect effects. It can be argued that CausalPath is more suitable for the analysis of short range effects due to the nature of pathway databases and due to the way CausalPath uses the pathway knowledge. To investigate this, we repeated the precision estimation study in the section "Precision of CausalPath results on cell lines stimulated with ligands", but this time using only earlier half of the time points or later half of the time points in the study. We were able to test a fewer number of relations in both cases because halving the dataset reduced the statistical power. Instead of 32, we were able to test 12 and 2 relations in the earlier and later time points, respectively (Fig. S7). "Early" test results were similar to the original results in shape, even though we have much fewer measurements to characterize it. In the "late" case, CausalPath generated fewer explanations for the proteomic changes upon ligand stimulation and the portion of them that are testable with the existing drug inhibitions only has 2 relations—both yield insignificant changes with drug inhibition. While this may be due to a worse precision, we do not have enough data to decide. It is possibly true that CausalPath can explain short time courses better than long time courses, however, in this case, it is better to use them together and have a higher statistical power for the paired t-test.

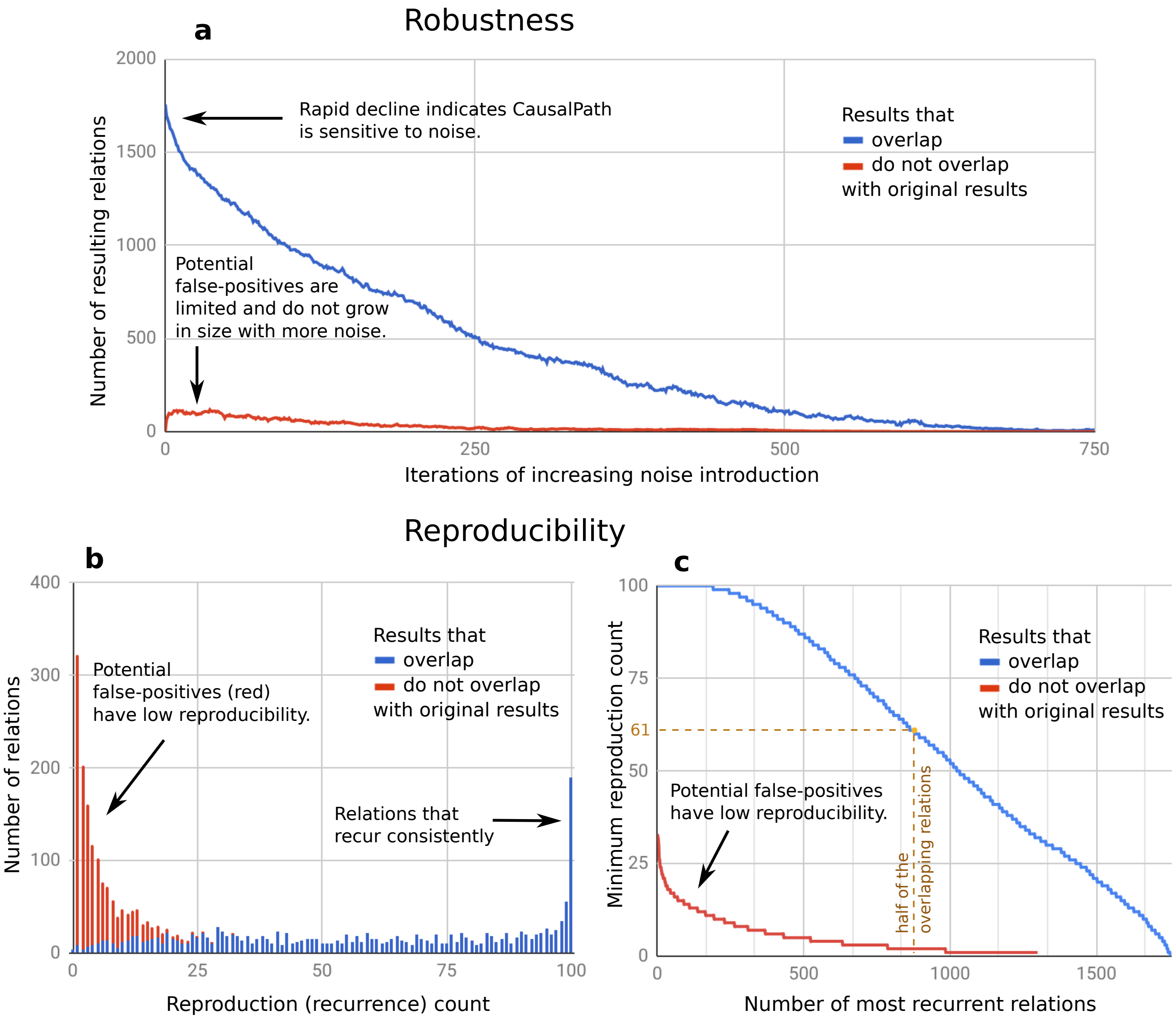

Figure S6: Robustness and reproducibility of CausalPath results on CPTAC breast cancer dataset with phospho relations. (a) Robustness is tested by iteratively introducing noise into proteomic data and then testing overlap of the new CausalPath results with the original results. (b, c) Reproducibility is tested by running CausalPath on random halves of samples. The new results are compared to the 1,756 relations in the original results where all of the samples were used. Non-overlapping results are likely dominated by false positives. The two reproducibility charts describe the same experiment from different viewpoints. Left chart (b) is a histogram of reproduction counts of result relations, while the right chart (c) is cumulative, showing the minimum reproduction ratios of the most reproducible result relations.

3.4 Evaluation of effect sizes in all analyses

When large datasets are analyzed for correlated or changed features, and a p-value threshold from a statistical test is used for identification of significant changes, it is possible that some features with very small effect sizes may pass the p-value threshold. When this happens, the conclusions derived from the results become questionable because of the small size of the effects. We tested if the analyzes in this manuscript are affected from small effect sizes and we have not detected any concerning cases. There are 391 individual CausalPath analyzes that contributed to this manuscript, corresponding to the same amount of analysis folders in the Suppl. Archive. Figure S8a provides an example distribution of effect sizes in a correlation-based study.

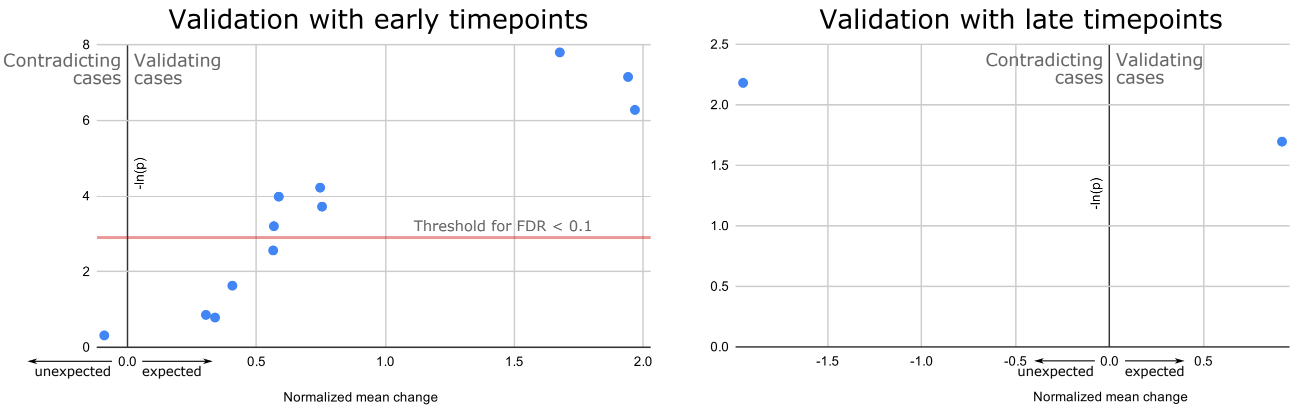

Figure S7: Results of limiting the precision estimation study to early or late time points.

Blue bars are the distribution of all pairwise Pearson correlations in the CPTAC breast cancer proteomic dataset (total protein and phoshpo combined), and the red bars indicate the count of selected features by CausalPath to use in causal hypotheses for explanation of phosphoproteomic changes, corresponding to the specific correlation range (blue and red bars are drawn in different scales, please notice two vertical axes). Here, we observe that there is no feature selection when the correlation is close to zero. In this example, the smallest absolute value of the selected correlations is 0.25. We checked how this smallest value changes in all the correlation-based analyses (Fig. S8b), and found that the average of the smallest values is 0.35, and the smallest of the smallest values is 0.12.

We did a similar check on correlation-based analyses. As an example, Figure S8c represents the comparison of luminal breast cancer to the basal subtype, again on CPTAC breast cancer proteomic dataset. Blue bars show the distribution of all changes, normalized with the standard deviation of that feature. Red bars are the distribution of the features that CausalPath selected for using in causal hypotheses (again, shown in a different scale). We observe that there are no red bars very close to zero change, indicating very small effect sizes are not used in the results. The smallest selected effect size in this analysis is 0.45 standard deviation. We checked how this smallest effect size changes in all comparison-based analyses, and found that the average of the smallest effect size is 1.15 and the smallest of the smallest effect sizes is 0.32 (Fig. S8d). (Note: Some of the comparison-based analyses use a paired t-test. In such cases, the normalization factor is not the standard deviation of the feature, but it is the standard deviation of all the paired difference values for that feature.)

3.5 Previously published methods for pathway analysis of proteomic datasets

There is no method comparable to CausalPath for its ability to identify causal relations from pathway databases that can explain given proteomic datasets. There are, however, methods developed for other forms of pathway analysis for proteomics. These methods generally use prior information in the form of networks stripped from mechanistic details, and aim to build a network structure that most fits to the profiling data at hand using an optimization method. During the development of CausalPath, instead of a network optimization, we intentionally focused on better usage of prior information by processing mechanistic details in pathway databases and using them in logical reasoning for causality. CausalPath, in that sense, is not competing with these alternative methods, but it is complementary to them. CausalPath can easily be paired with any other network optimization method for further complexity management and for using strong priors in network optimization. Below is a short survey of these other pathway analysis methods.

3.5.1 Temporal Pathway Synthesizer (TPS)

TPS uses PPI and kinase-substrate networks in the process of inferring signaling relations from temporal post-perturbation proteomic data [1]. The method first runs a prize-collecting Steiner tree (PCST) algorithm between the perturbed agent and the modified proteins to reduce the PPI network, then it uses this reduced

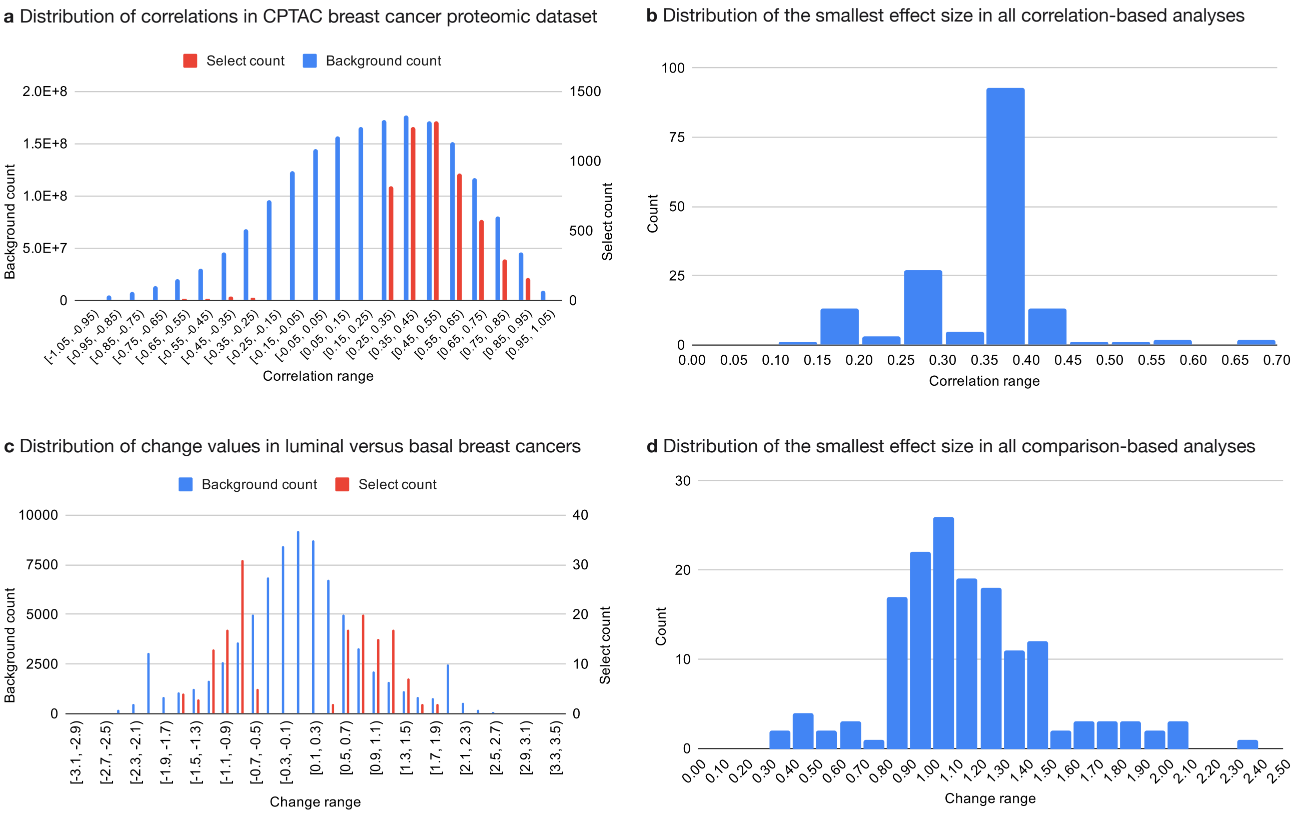

Figure S8: Illustration of the effects sizes used in causal hypothesis generation. Upper charts are related to correlation-based analyses and lower charts are related to comparison-based analyses. Charts on the left are example cases from CPTAC breast cancer proteomic dataset. Charts on the right are the overall distributions of the smallest effect size in each analysis. The average background correlation on (a) is 0.23 and the average background change value on (c) is -0.03.

network and the substrate-kinase network as constraints in its search for a model that do not violate temporal constraints (upstream changes are not happening after downstream changes) observed in the data. TPS does not consider if a measured phosphorylation site is activating or inhibiting of its downstream activity, and it does not check if the kinase phosphorylates from the matching location of the substrate observed in the data. Also, TPS is limited to perturbation studies with temporal proteomic profiling.

The EGF stimulation study that we analyze in the results section was originally produced by the authors of the TPS method to demonstrate its capabilities. A comparison of our results with theirs indicates large differences in resulting models. TPS finds 413 directed relations that fit in the constraints of the system. CausalPath, on the other hand, identifies 50 relations collectively for all time frames (all unique relations in the Suppl. Animation 2). Only 3 of these relations are overlapping, one of them being EGF $\rightarrow$ EGFR. There are many reasons for this huge difference in results. TPS is more focused on evaluating the temporal constraints and less focused on the strength of the priors. For example, when two proteins are known to be interacting and this interaction is selected by PCST, TPS algorithm puts a directed edge between them according to the timing of their phosphorylation changes. From CausalPath perspective, this is a very loose inference because a protein interaction is very weak evidence for a causal effect. CausalPath looks for prior knowledge of a phosphorylation relation and the knowledge of site-effect for the regulator. This results in fewer relations but stronger hypotheses due to their evidence of existence in at least one experimental setup. From TPS perspective, CausalPath misses the information embedded in the temporal order of the phosphorylations. During the development of CausalPath, we considered evaluating the order of phosphorylations when this data is available but decided against it due to several reasons. The most important reason lies in the nature of statistical detection methods that we use. When we detect a phosphorylation change, comparing cases against controls using a statistical method, we often choose a stringent significance threshold to keep the false positives under a certain amount. But a stringent threshold practically means a high level of false negatives—the cases that we miss to detect. Accordingly, we can trust the positives in the results more than the negatives. This is generally acceptable when we base our interpretations only on the positive results, just like we do in CausalPath, finding the explanatory causal relations between positive results. However, when we use negative results in the interpretation, such as *lack* of phosphorylation change, the high false negative rate would cause a high rate of misinterpretations. For instance, to infer a directed relation from A to B, TPS requires B is not phosphorylated before A, however, due to the high rate of false negatives, B may be actually phosphorylated before A but not showing up in the results. To properly check for this constraint, one should design an experimental setup and a companion statistical test that evaluate the timing of peptide changes, and detect one peptide increased/decreased *significantly sooner/later* than the other. The second reason for our not focusing on temporal order is that we consider that an effect can have multiple causes. Cause 1 may generate the Effect at an early time point, then Cause 2 can appear and start supporting the Effect in a later time point. We would not like to miss such multi-cause effects, especially when they are already documented in the literature, and considering Cause 1 may be invisible to the analysis.

We tried to estimate the precision of TPS using the same procedure that we applied to CausalPath in the main manuscript, section "Precision of CausalPath results on cell lines stimulated with ligands". This evaluation is not straightforward because TPS does not claim to generate falsifiable hypotheses as CausalPath does, but only generates a relevant network, where the relevancy is not biologically well-defined. However, the presence of directed edges in the output network, and the requirement of temporal evidence implies causality between the mapped proteomic measurements. We assessed the precision of this implication using the RPPA experiment results from [4] where cell lines are treated with protein ligands and inhibitor drugs. We generated a result network for each cell line - ligand stimulation experiment, then we used the drug-treated samples to validate directed relations in the results. More specifically, if the source protein of a directed edge in the results has a prediction of "activation" on a time point, and that protein is targeted by a drug in the experiments, we expected the measured feature of the downstream protein of the relation will change in the reverse direction when the drug is applied, on the same or later time points. Using the measurements on 4 cell lines with 6 ligands (skipping PBS case because TPS requires the target of perturbation as input) and 5 drugs allowed us to test a total of 54 directed edges in TPS results. Out of 54, we observed a change towards the expected direction in 34 cases, and towards the unexpected direction in 20 cases (Fig. S9). 6 of the changes pass a 0.1 FDR threshold, where 5 of them are in the expected direction. While we observe a higher number of changes in the expected direction, this imbalance is not significant (P

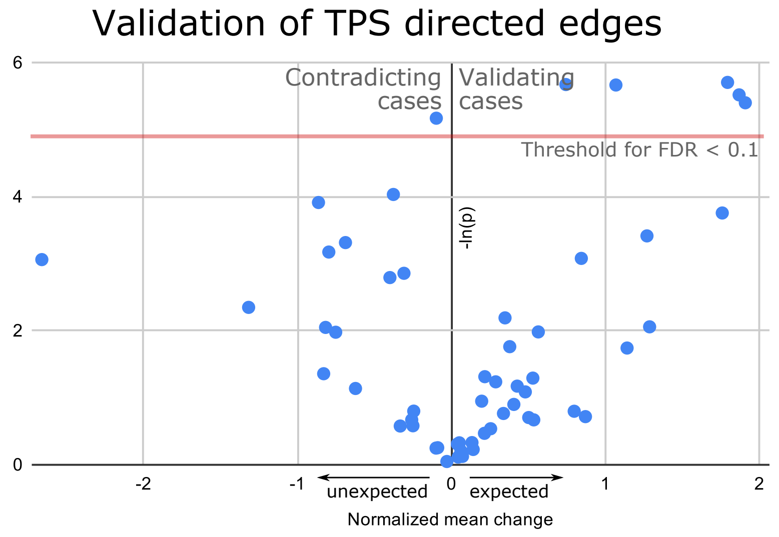

Figure S9: Evaluation of the precision of TPS directed edges on the RPPA experiment of ligand-stimulation and drug-treatment of breast cancer cell lines. Positive direction on the x-axis is the expected direction of change of downstream proteins upon drug treatment. Negative is the unexpected direction. Red line indicates a threshold of 0.1 FDR in the detected changes.

= 0.075, binomial test). If we use the same symmetrical-noise assumption that we used for estimating the precision of CausalPath, we assume all 20 changes on the unexpected direction are noise and expect to have the same amount on the expected direction. This predicts that 14 surplus changes on the expected direction are actual changes, which means 14 of 54 cases validate positive, amounting to a precision of 0.26. The less-specific use of prior knowledge by TPS is the most likely cause for this lower precision. A second factor is the reduced statistical power. The temporal angle of TPS makes it require more data for similar statistical power. Limiting a t-test to a specific time window reduces the number of samples used in the calculation. We predict if there were more replicates in the RPPA experiment, TPS precision would increase. The input and output files for this analysis can be found in the Suppl. Archive.

3.5.2 PARADIGM

PARADIGM is one of the earliest pathway analysis methods developed originally for RNA expression and copy number variations and later extended to other data types including proteomics [5]. It uses the pathway models from NCI Pathway Interaction Database (PID), converts them into a factor graph, and predicts each entity's activity level using an expectation-maximization algorithm. PARADIGM does not provide any site-specific data handling. The method assumes each mapped data type is a positive indicator of activity, and does not differentiate between activating and inhibiting phosphorylations. It also does not check if a site-specific phosphorylation has a downstream phosphorylation change at the matching site.

3.5.3 pCHIPS

This is a network propagation method for proteomic and other data, based on the TieDIE [6] algorithm, where the purpose is to link differentially active kinases (indicated by proteomic data) to the differentially active transcription factors (indicated by RNAseq measurements of targets) [7]. Proteomic changes on the kinases are propagated downstream, differential transcription factor activities are propagated upstream on the signaling network, and the overlap is identified as a possible linking path or combination of paths.

While linking kinases to transcription factors implies causality, pCHIPS does not check the conditions of causality such as if the proteomic change is indicative of activation or inhibition, or if the linking path has a positive or a negative effect on the transcription factor activity, or their compatibility for a causality hypothesis.

3.5.4 SigNetTrainer

SigNetTrainer [8] is a set of algorithms that score the fitness of molecular readouts to a given set of perturbations and a given directed and signed interaction graph, and solves several interesting problems using integer linear programming, such as finding an optimal subgraph that is most consistent with the measurements, or finding minimal set of new relations that will make the network and the measurements consistent. SigNetTrainer does not perform any phosphorylation site-specific operation and does not differentiate between activating and inhibiting phosphorylations.

3.5.5 PHONEMeS

PHONEMeS [(9)] builds models in the form of Boolean networks that best fit to a given set of phosphoproteomic perturbation data. It uses a background network derived from prior information, and searches for the best Boolean network that is predictive for the given set of perturbations. The Boolean framework assumes that every protein has two discrete states, hence PHONEMeS checks the data if evidence for such a dichotomy exists, and uses only those phosphopeptides. PHONEMeS does not check for any site matching constraint, and it does not distinguish between activating/inhibiting phosphorylations.

3.5.6 Method from Chasman *et al.*

Chasman *et al.* demonstrate their network inference method on identification of yeast adaptive pathways to NaCl stress [10]. They compile a background network with directed and undirected relations, identify a set of genes/proteins by differential expression, phosphoproteome changes and stress fitness contribution, and find optimal paths from signaling proteins to gene regulation proteins employing integer programming (IP). Their method does not perform any site-specific evaluation of phosphorylations. Also the method does not distinguish between increased and decreased phosphopeptides, but considers them as *changed*.

3.5.7 PhosphoPath and PTMapper

Both methods are implemented as a Cytoscape plugin to visualize kinase-substrate relations on the protein-protein interaction (PPI) network [11; 12]. Users can run a network enrichment analysis on the PPI network for the given proteomic and other datasets, then visualize the known kinase-substrate relations on the enriched region. Neither of the methods perform any site-specific operation or evaluation.

3.5.8 PCST

This method maps proteomic and transcriptomic data on the proteins on a weighted PPI and protein-DNA interaction network, then identifies a minimal subnetwork that connects the mapped molecules, prioritizing the most reliable connections [13]. Authors formulate this as a prize-collecting Steiner tree (PCST) problem and solve with a known algorithm.

3.5.9 PHOTON

This method maps proteomic data from a perturbation study onto the proteins on a weighted PPI network, then calculates a score for each protein based on the weighted average of the observed proteomic changes on its neighbors on the network [14]. The method generates a result network by connecting the perturbed protein and the proteins with a high score on the PPI network.

3.6 Library of graphical patterns used for causality detection

Here we provide all 12 graphical BioPAX patterns that CausalPath uses to understand phosphorylation, dephosphorylation, expression upregulation, and expression downregulation relations in Pathway Commons. Each figure shows the pattern structure, a sample BioPAX data as rendered in SBGN by ChiBE, and the identified relations from the sample BioPAX using the pattern. Understanding the pattern structure requires the basic knowledge of BioPAX data structure. These patterns are implemented using the BioPAX-pattern framework, which is a Java library.

The patterns are composed of labeled BioPAX objects and certain relations between them. A common component in all patterns is the handling of generic molecules and complexes, which can be arbitrarily nested. The relation "self or more generic" allows to link the molecule towards parent generics and encapsulating complexes, the relation "self or less generic" allows link toward specific proteins and complex members.

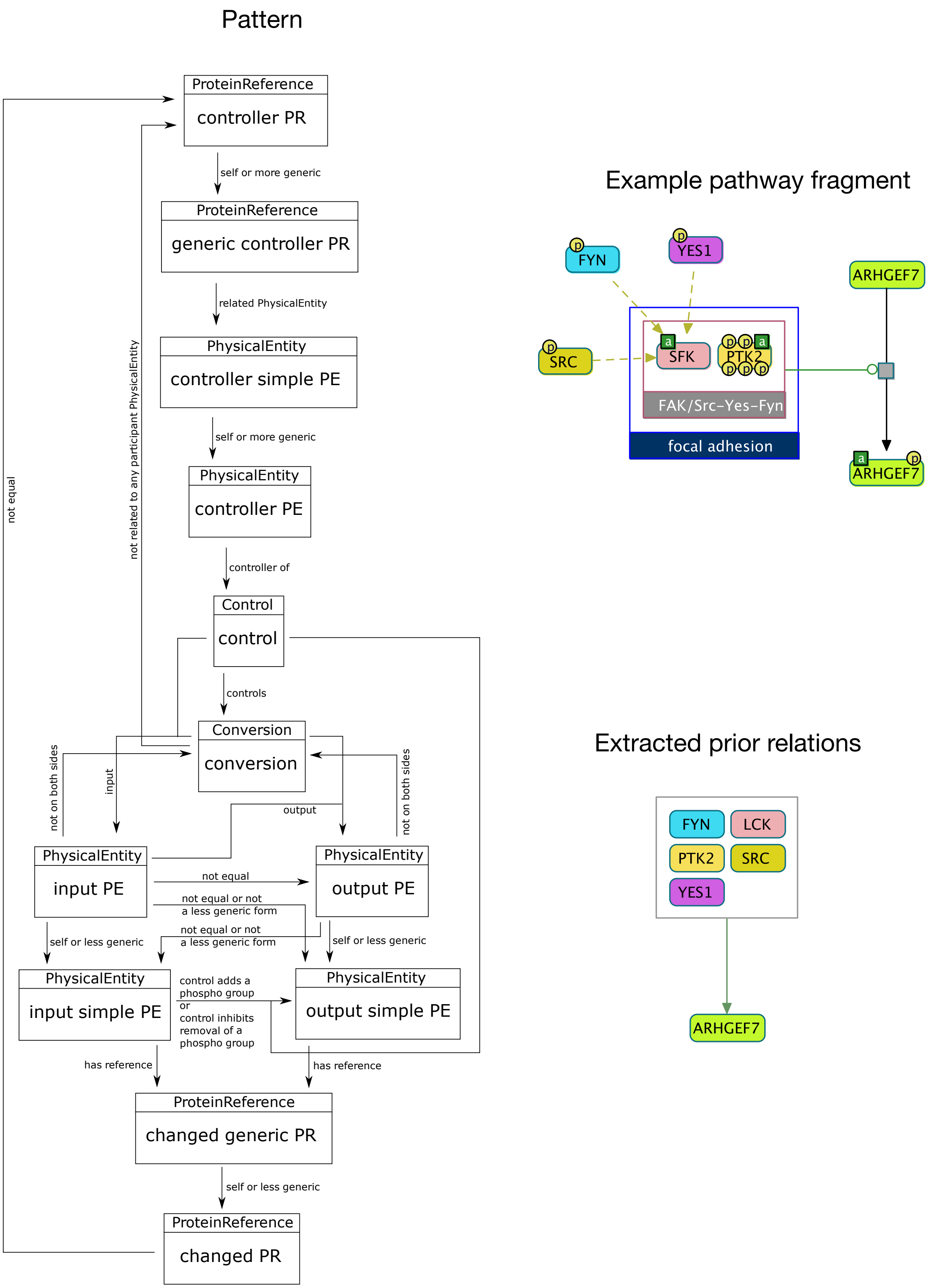

Figure S10: Pattern 1 for phosphorylation. This is the most frequently used pattern to describe phosphorylations where the regulator proteins activate a Conversion that adds phosphorylation to a protein, or inhibit a Conversion that removes phosphorylation from the downstream protein.

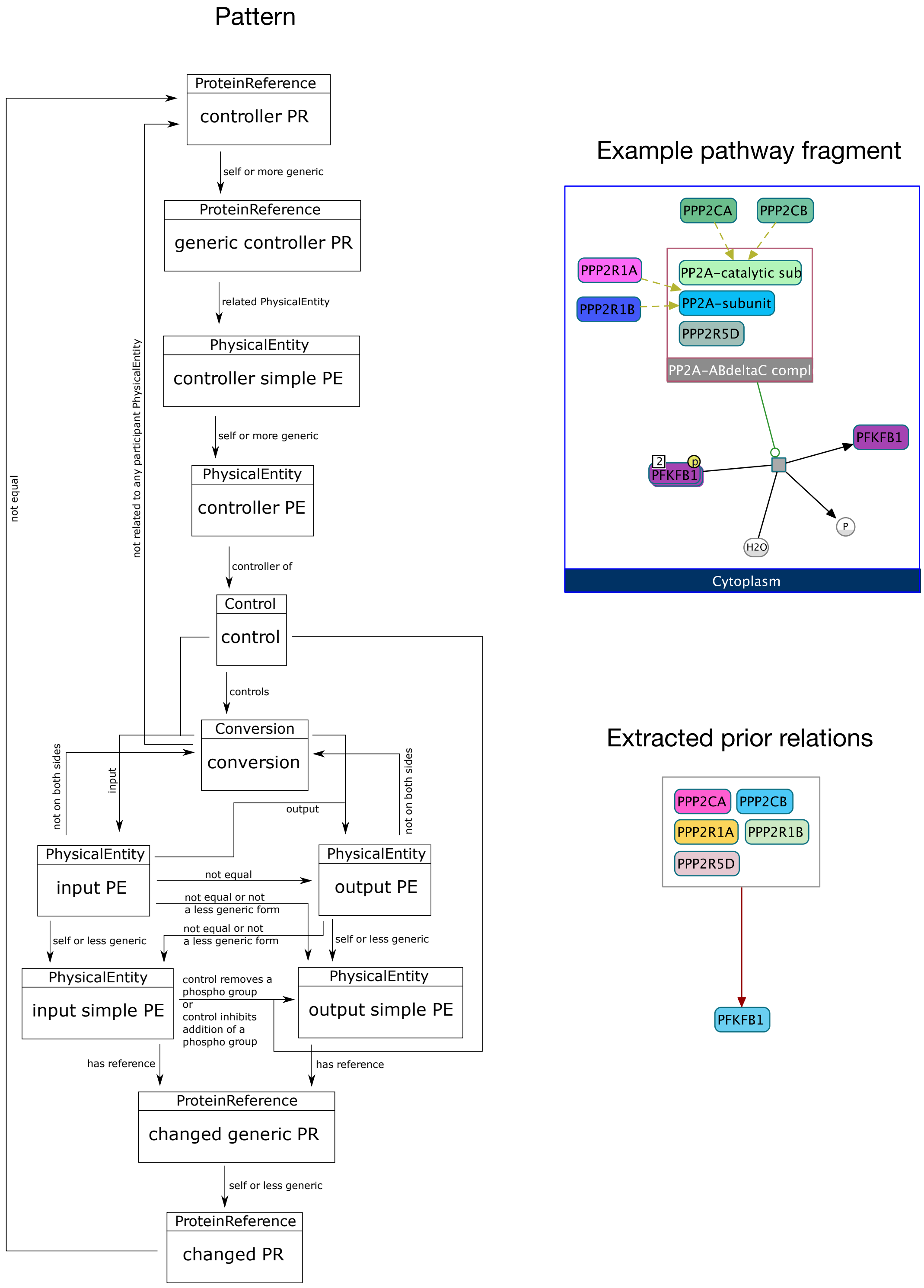

Figure S11: Pattern 1 for dephosphorylation. This is the most frequently used pattern to describe dephosphorylations where the regulator proteins activate a Conversion that removes phosphorylation from a protein, or inhibit a Conversion that adds phosphorylation to the downstream protein.

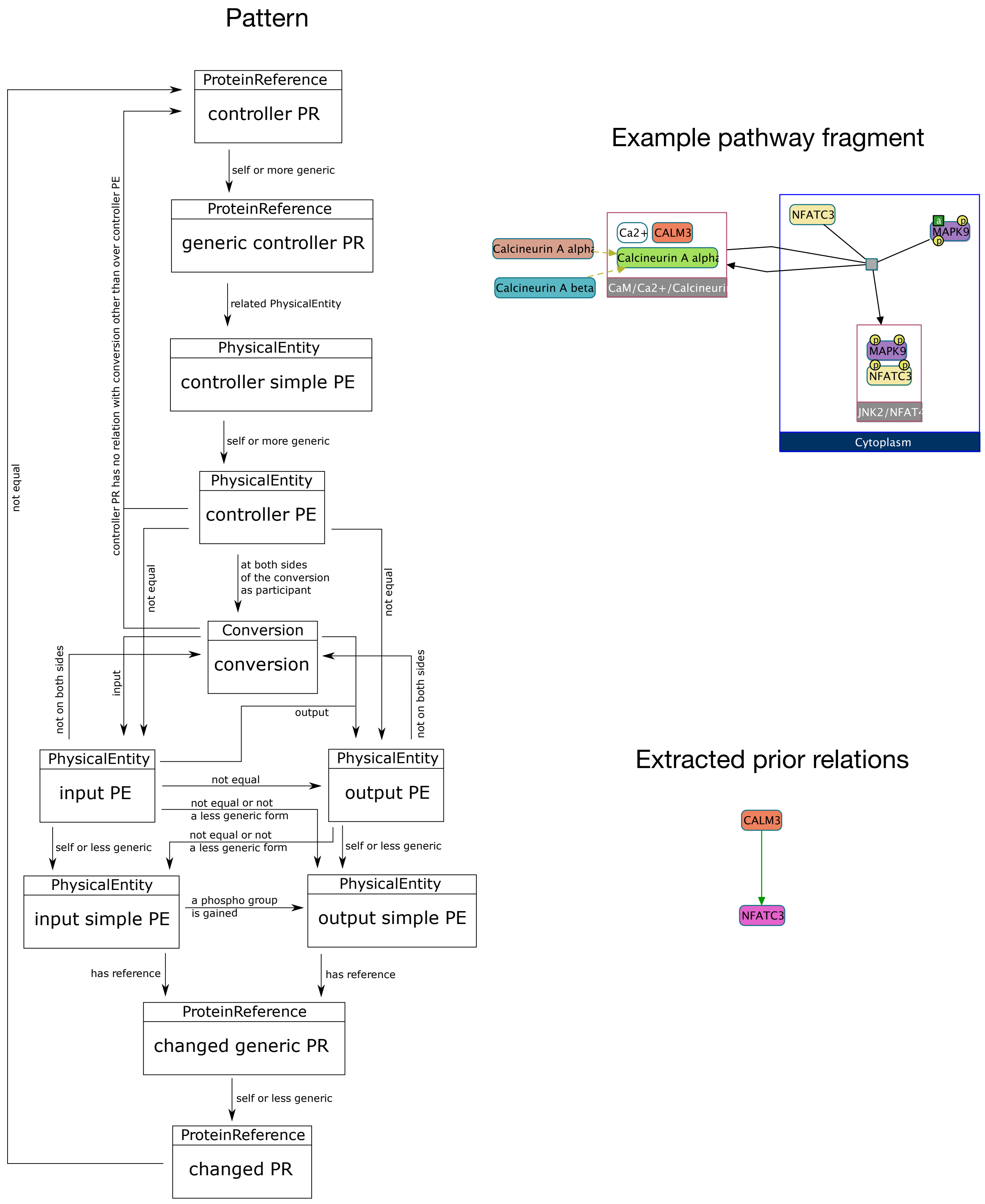

Figure S12: Pattern 2 for phosphorylation. This pattern captures the cases where the regulator proteins are not modeled as regulators of a Conversion, but modeled as inputs and outputs of the same Conversion.

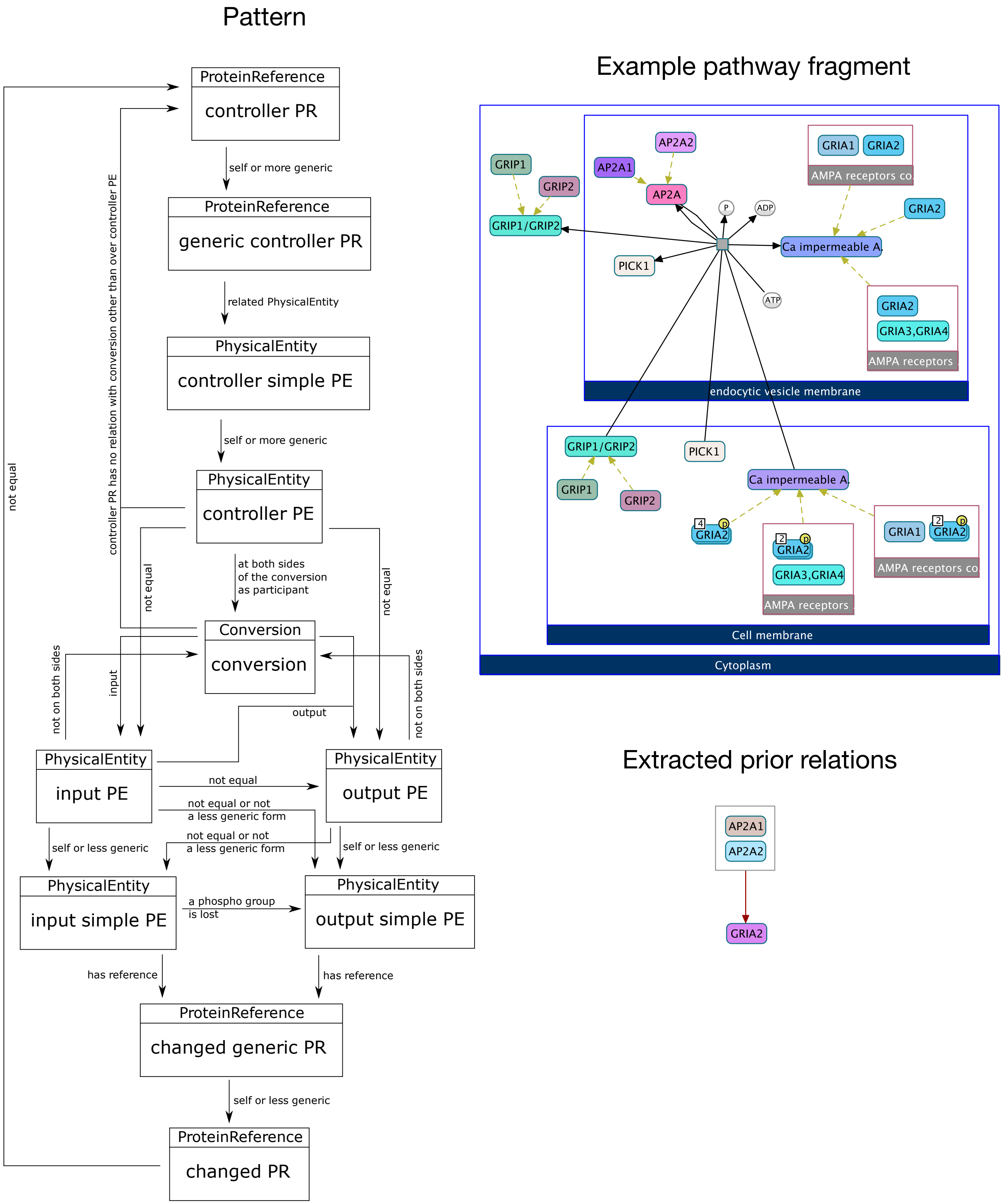

Figure S13: Pattern 2 for dephosphorylation. This pattern captures the cases where the regulator proteins are not modeled as regulators of a Conversion, but modeled as inputs and outputs of the same Conversion.

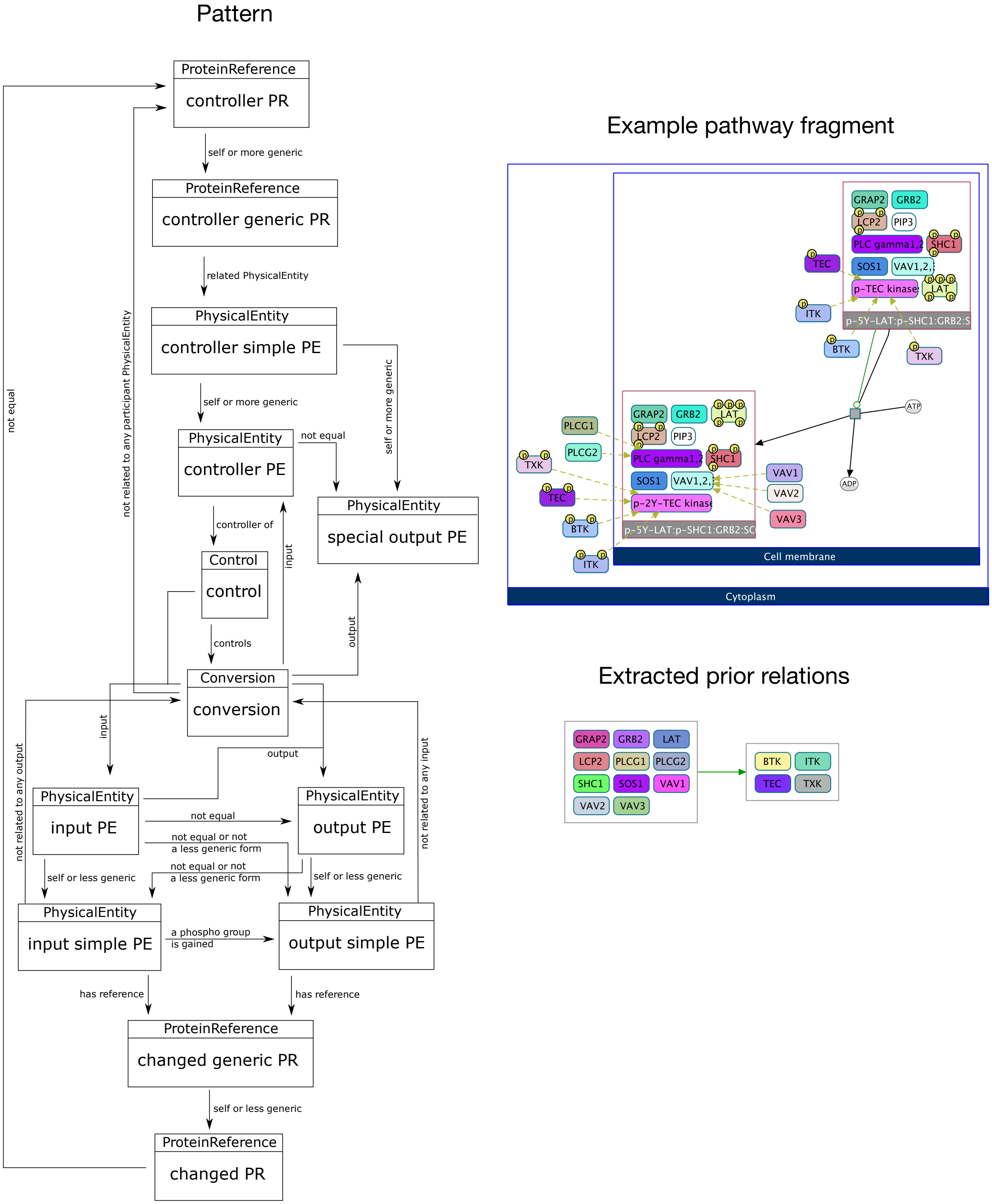

Figure S14: Pattern 3 for phosphorylation. This pattern captures the cases where some proteins in a complex are phosphorylated, and the input complex is also designated to be the controller of the Conversion.

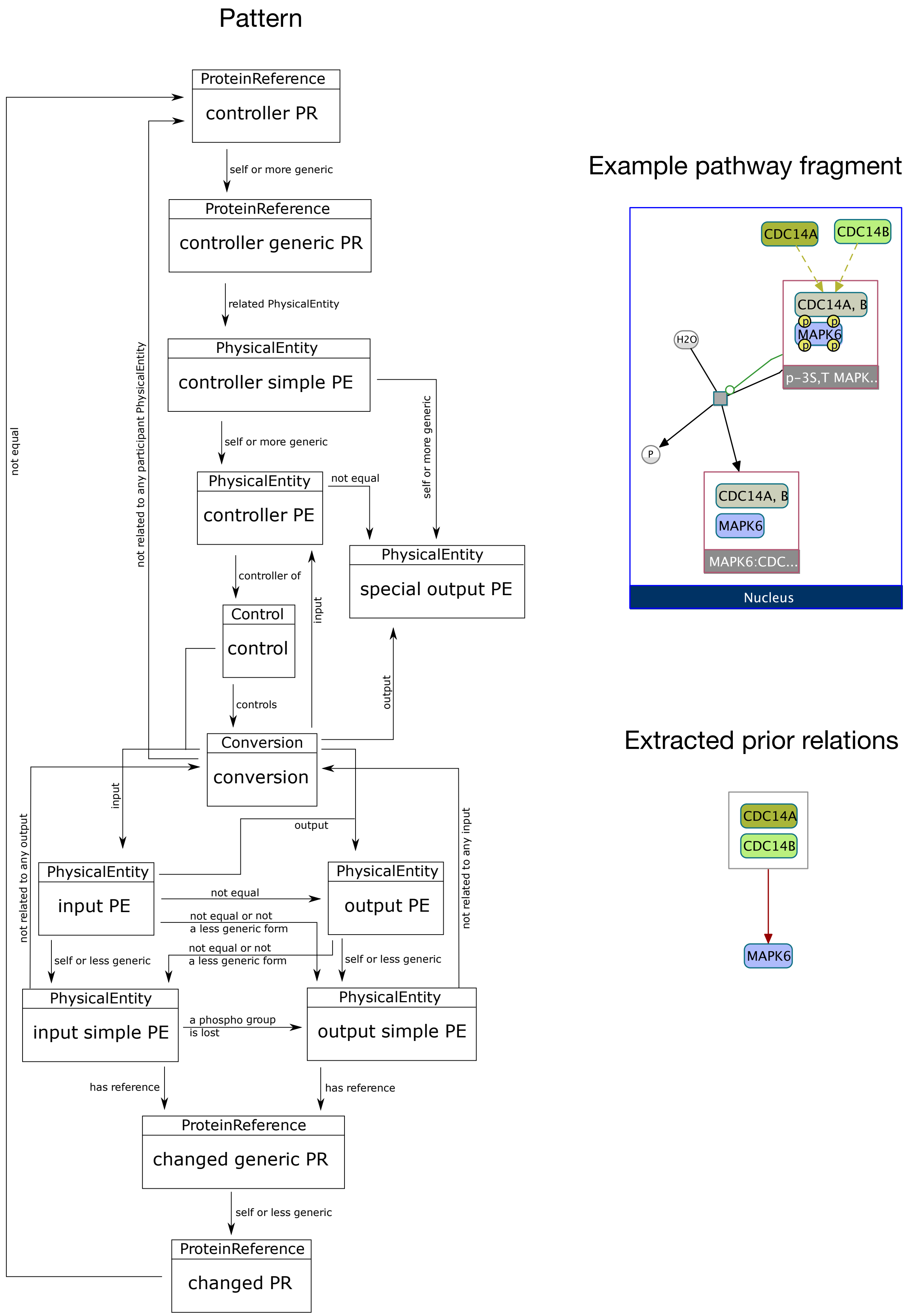

Figure S15: Pattern 3 for dephosphorylation. This pattern captures the cases where some proteins in a complex are phosphorylated, and the input complex is also designated to be the controller of the Conversion.

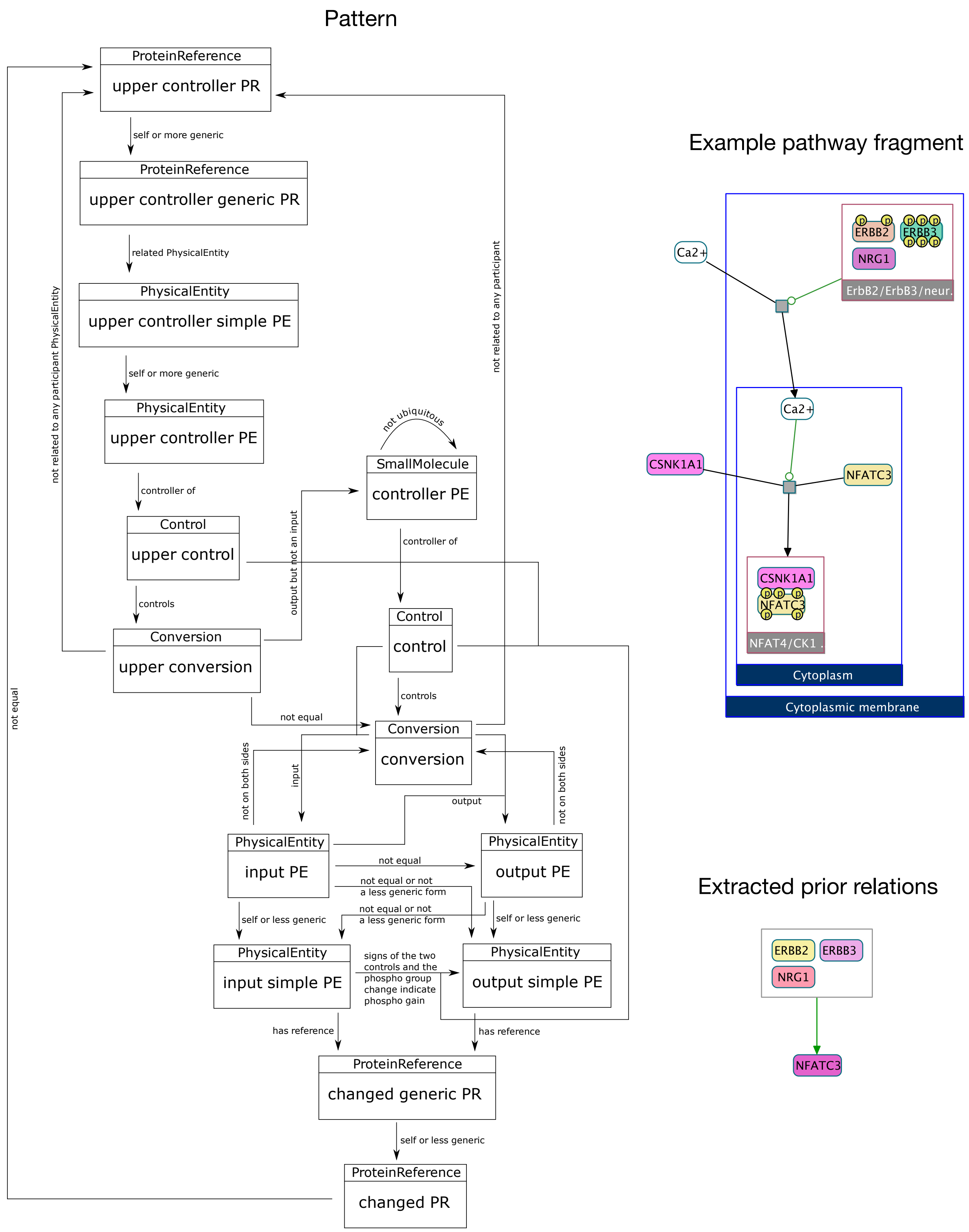

Figure S16: Pattern 4 for phosphorylation. This pattern captures the cases where the controller transmits its effect through a controlling small molecule.

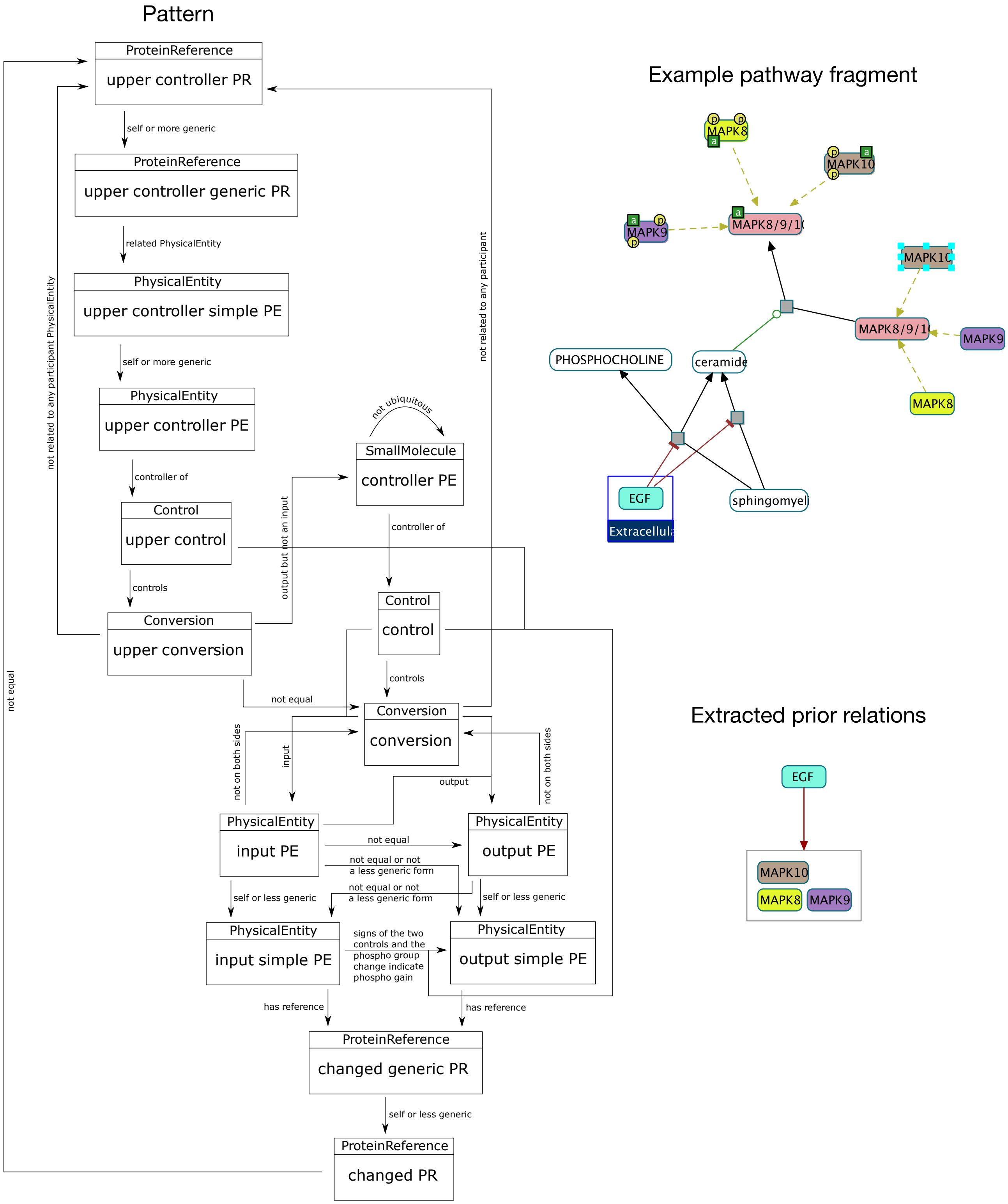

Figure S17: Pattern 4 for dephosphorylation. This pattern captures the cases where the controller transmits its effect through a controlling small molecule.

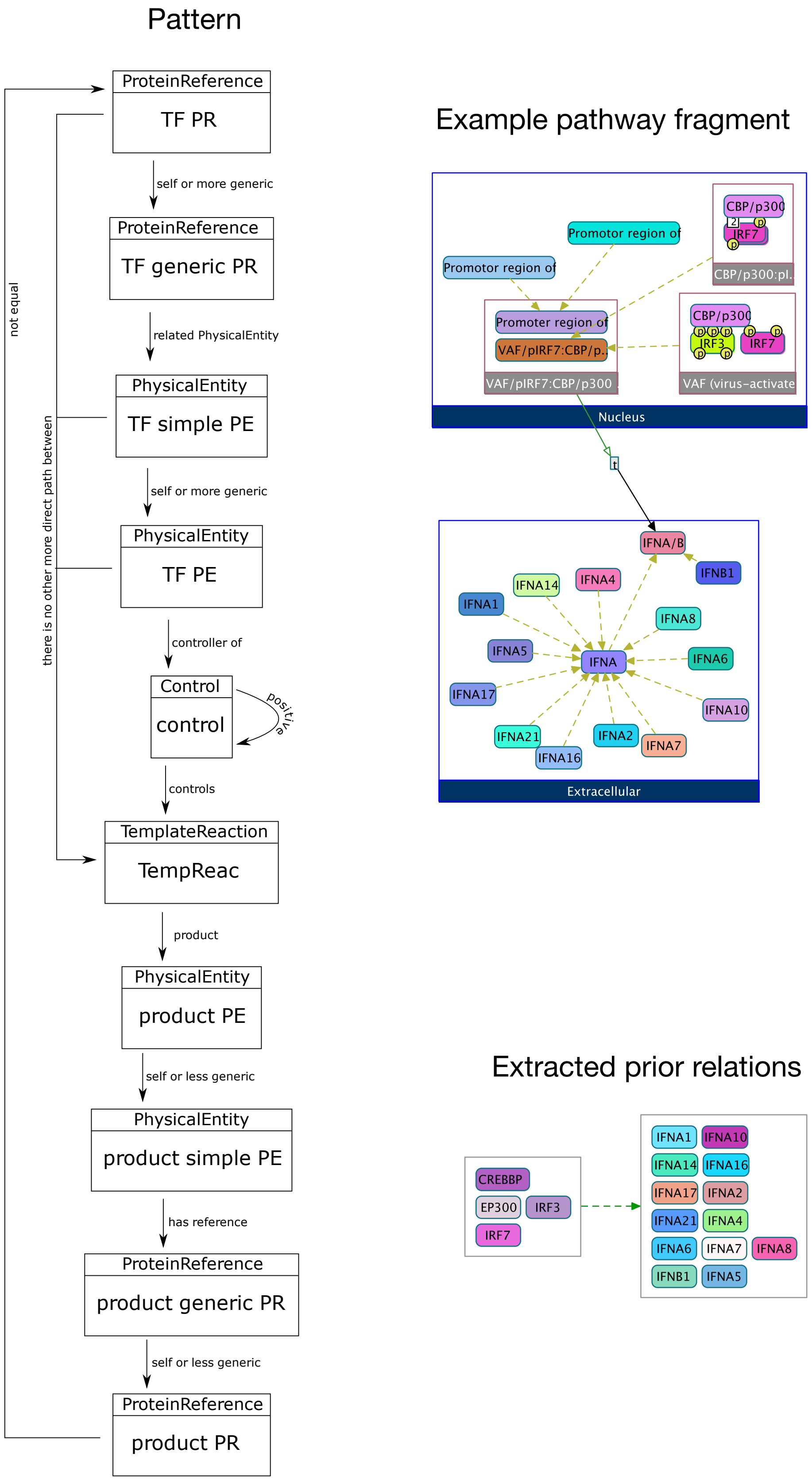

Figure S18: Pattern 1 for expression upregulation. This is the most frequent pattern for expression upregulation where the regulator activates a TemplateReaction.

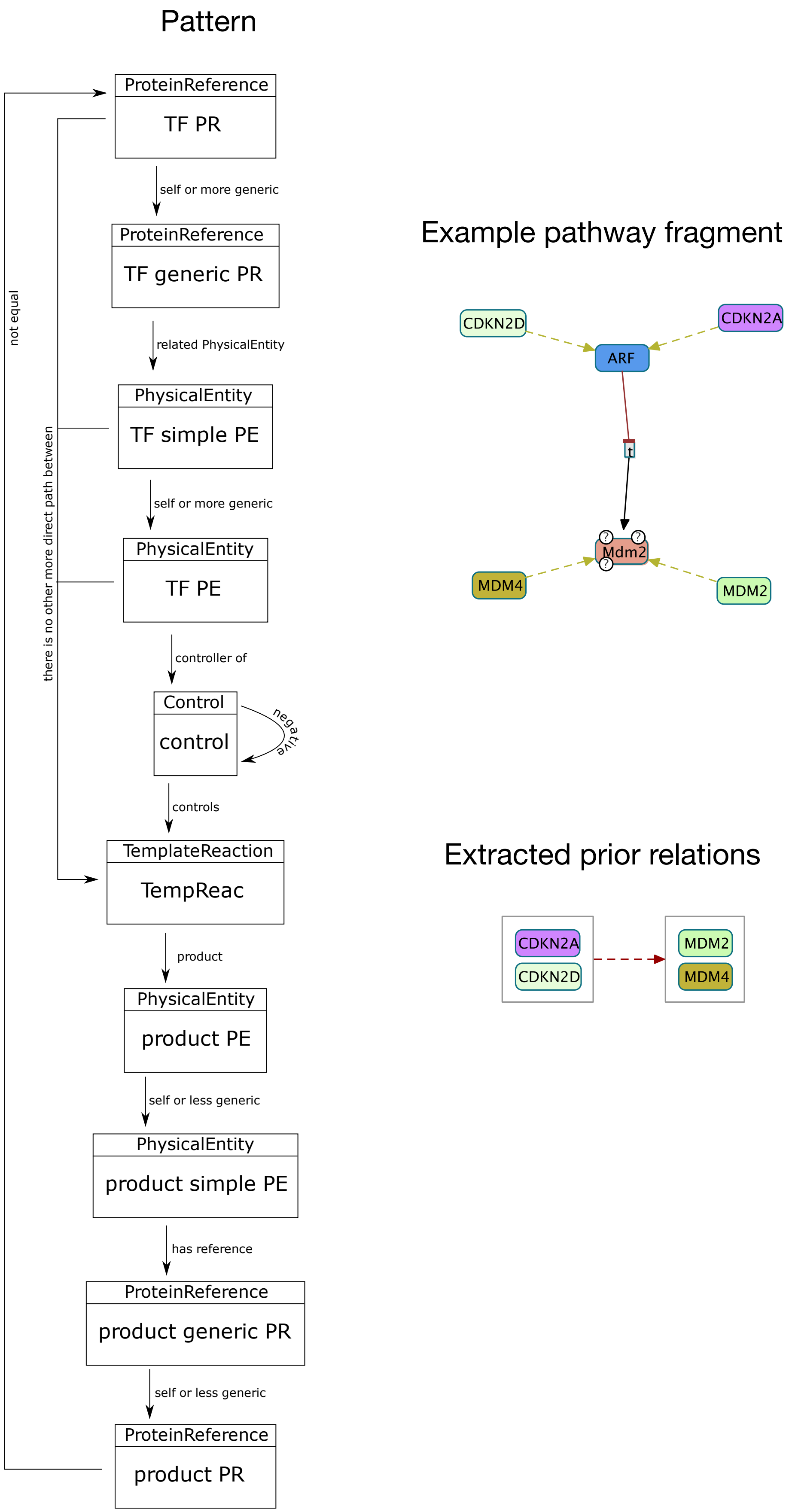

Figure S19: Pattern 1 for expression downregulation. This is the most frequent pattern for expression downregulation where the regulator inhibits a TemplateReaction.

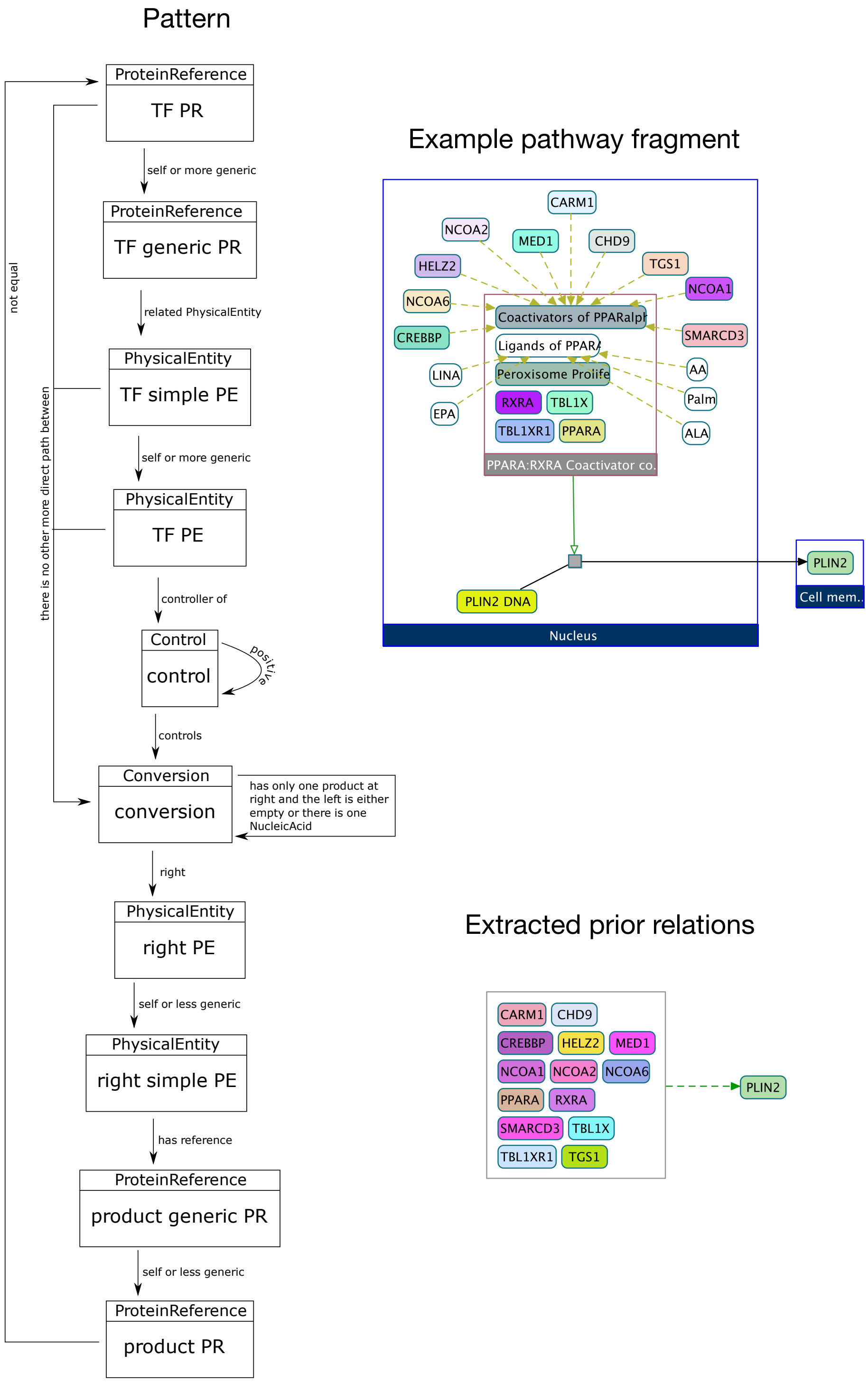

Figure S20: Pattern 2 for expression upregulation. This pattern captures the cases where a Conversion is used instead of a TemplateReaction.

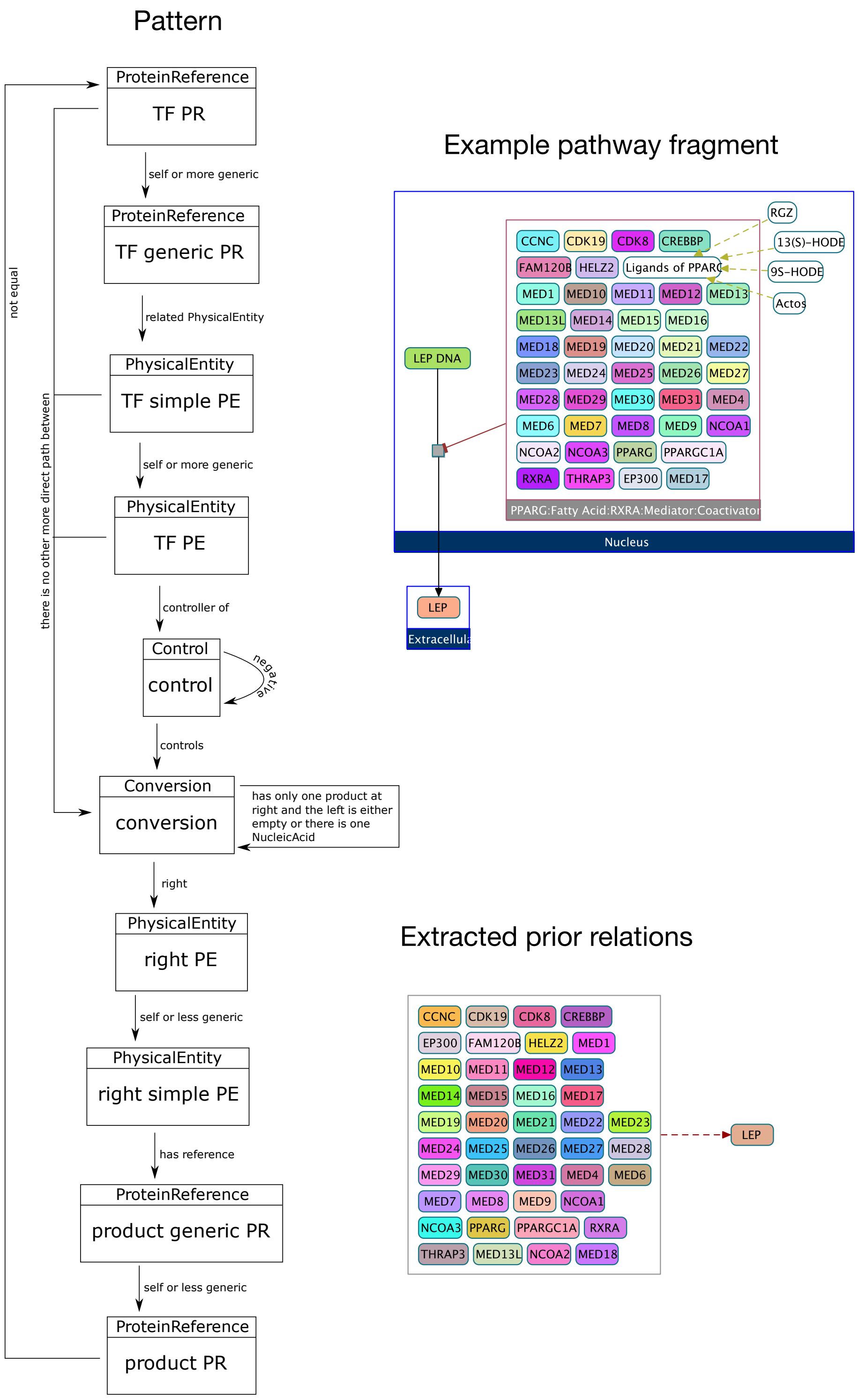

Figure S21: Pattern 2 for expression downregulation. This pattern captures the cases where a Conversion is used instead of a TemplateReaction.

3.7 Extending graphical patterns

The graphical patterns listed in this document are developed considering the available pathway relations in Pathway Commons v9, and implemented in the GitHub project at https://github.com/PathwayAndDataAnalysis/causal-priors-extractor. These patterns can be extended to either cover new data sources or to capture new relationship types. For new data sources, it is very likely that the listed patterns will be applicable as is, however not guaranteed. When needed, the graph structures in the new data source should be investigated and new graph structures should be captured by adding new patterns to the framework. Adding a new pattern can be done by adding a new class that extends from `org.biopax.paxtools.pattern.miner.MinerAdapter` (or one of its children) and override the method `constructPattern()`, just like the other patterns in the project.

The procedure is similar for capturing new types of relationships. Developers need to study the structure of the existing BioPAX data to understand in what forms the information is encoded. Then a pattern can be built to detect those structures. This process requires an understanding of BioPAX [15] and the BioPAX-pattern framework [16, https://github.com/BioPAX/Paxtools/tree/master/pattern].

References

[1] Köksal AS, Beck K, Cronin DR, McKenna A, Camp ND, Srivastava S, et al. Synthesizing signaling pathways from temporal phosphoproteomic data. Cell reports 2018;24(13):3607–18.

[2] Suppes P. A probabilistic theory of causality. North-Holland Publishing Company Amsterdam; 1970.

[3] Pearl J. Causality: models, reasoning and inference. Econometric Theory 2003;19(675-685):46.

[4] Hill SM, Nesser NK, Johnson-Camacho K, Jeffress M, Johnson A, Boniface C, et al. Context specificity in causal signaling networks revealed by phosphoprotein profiling. Cell systems 2017;4(1):73–83.

[5] Vaske CJ, Benz SC, Sanborn JZ, Earl D, Szeto C, Zhu J, et al. Inference of patient-specific pathway activities from multi-dimensional cancer genomics data using paradigm. Bioinformatics 2010;26(12):i237–45.

[6] Paull EO, Carlin DE, Niepel M, Sorger PK, Haussler D, Stuart JM. Discovering causal pathways linking genomic events to transcriptional states using tied diffusion through interacting events (tiedie). Bioinformatics 2013;29(21):2757–64.

[7] Drake JM, Paull EO, Graham NA, Lee JK, Smith BA, Titz B, et al. Phosphoproteome integration reveals patient-specific networks in prostate cancer. Cell 2016;166(4):1041–54.

[8] Melas IN, Samaga R, Alexopoulos LG, Klamt S. Detecting and removing inconsistencies between experimental data and signaling network topologies using integer linear programming on interaction graphs. PLoS computational biology 2013;9(9):e1003204.

[9] Terfve CD, Wilkes EH, Casado P, Cutillas PR, Saez-Rodriguez J. Large-scale models of signal propagation in human cells derived from discovery phosphoproteomic data. Nature communications 2015;6:8033.

[10] Chasman D, Ho YH, Berry DB, Nemec CM, MacGilvray ME, Hose J, et al. Pathway connectivity and signaling coordination in the yeast stress-activated signaling network. Molecular systems biology 2014;10(11):759.

[11] Raaijmakers LM, Giansanti P, Possik PA, Mueller J, Peeper DS, Heck AJ, et al. Phosphopath: Visualization of phosphosite-centric dynamics in temporal molecular networks. Journal of proteome research 2015;14(10):4332–41.

[12] Narushima Y, Kozuka-Hata H, Tsumoto K, Inoue JI, Oyama M. Quantitative phosphoproteomics-based molecular network description for high-resolution kinase-substrate interactome analysis. Bioinformatics 2016;:btw164.

[13] Huang J, Zhang T, Linstroth L, Tillman Z, Otegui MS, Owen HA, et al. Control of anther cell differentiation by the small protein ligand tpd1 and its receptor ems1 in arabidopsis. PLOS Genet 2016;12(8):e1006147.

[14] Rudolph JD, de Graauw M, van de Water B, Geiger T, Sharan R. Elucidation of signaling pathways from large-scale phosphoproteomic data using protein interaction networks. Cell Systems 2016;3(6):585–93.

[15] Demir E, Cary MP, Paley S, Fukuda K, Lemer C, Vastrik I, et al. The biopax community standard for pathway data sharing. Nature Biotechnology 2010;28(9):935–42.

[16] Babur Ö, Aksoy BA, Rodchenkov I, Sümer SO, Sander C, Demir E. Pattern search in BioPAX models. Bioinformatics 2014;30(1):139–40.
