# Supplementary material for: Causal interactions from proteomic profiles: Molecular data meet pathway knowledge
Source: Patterns (N Y). 2021 May 12;2(6):100257. doi: 10.1016/j.patter.2021.100257 (PMC8212145; doi:10.1016/j.patter.2021.100257)
Supplement: Document S2. Article plus supplemental information [file mmc6.pdf]

Article

Patterns

Causal interactions from proteomic profiles: Molecular data meet pathway knowledge

Graphical abstract

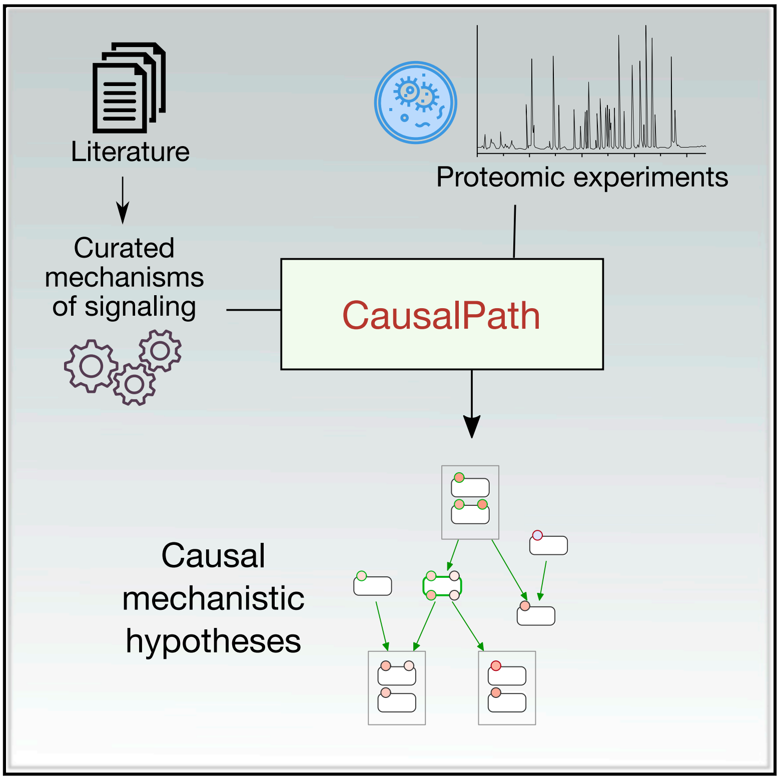

Highlights

- CausalPath builds mechanistic models from proteomic profiles
- It integrates biological pathway models with molecular measurements
- It supports logical reasoning with post-translational modifications
- A web server, free software, and a source code are available

Authors

Özgün Babur, Augustin Luna, Anil Korkut, ..., Joseph E. Aslan, Chris Sander, Emek Demir

Correspondence

ozgun.babur@umb.edu

In brief

CausalPath integrates detailed biological pathways with proteomic and other molecular profiles to generate mechanistic models explaining how the observed changes are related. It is applicable to a wide range of contexts and a variety of experiment types. The method can be accessed at causalpath.org.

Babur et al., 2021, Patterns 2, 100257
June 11, 2021 © 2021 The Author(s).
https://doi.org/10.1016/j.patter.2021.100257

CellPress

Patterns

CellPress
OPEN ACCESS

Article

Causal interactions from proteomic profiles: Molecular data meet pathway knowledge

Özgün Babur,[1,11,*] Augustin Luna,[2] Anil Korkut,[3] Funda Durupinar,[1] Metin Can Siper,[4] Ugur Dogrusoz,[5] Alvaro Sebastian Vaca Jacome,[6] Ryan Peckner,[6,7] Karen E. Christianson,[6] Jacob D. Jaffe,[6] Paul T. Spellman,[4,8] Joseph E. Aslan,[9] Chris Sander,[2] and Emek Demir[4,8,10]

[1]Computer Science Department, University of Massachusetts Boston, 100 William T. Morrissey Boulevard, Boston, MA 02125, USA
[2]cBio Center for Computational and Systems Biology, Dana-Farber Cancer Institute and Department of Cell Biology, Harvard Medical School, Boston, MA 02215, USA
[3]Department of Bioinformatics and Computational Biology, The University of Texas MD Anderson Cancer Center, Houston, TX 77030, USA
[4]Computational Biology Program, Oregon Health and Science University, 3181 SW Sam Jackson Park Road, Portland, OR 97239, USA
[5]Computer Engineering Department, Bilkent University, Ankara 06800, Turkey
[6]The Broad Institute of MIT and Harvard, Cambridge, MA 02142, USA
[7]Cogen Therapeutics, Cambridge, MA 02139, USA
[8]Department of Molecular and Medical Genetics, Oregon Health and Science University, 3181 SW Sam Jackson Park Road, Portland, OR 97239, USA
[9]Knight Cardiovascular Institute, Oregon Health and Science University, 3181 SW Sam Jackson Park Road, Portland, OR 97239, USA
[10]Pacific Northwest National Laboratories, 902 Battelle Boulevard, Richland, WA 99354, USA
[11]Lead contact
*Correspondence: ozgun.babur@umb.edu
https://doi.org/10.1016/j.patter.2021.100257

THE BIGGER PICTURE Molecular profiling of biological organisms provides us with a great amount of information on cellular differences, but converting it to mechanistic insights is still a very challenging task. A prominent approach is to integrate new measurements with the mechanistic knowledge described in the scientific literature and build a model that is supported by both. Although this can be done in many ways, an adept approach will use the literature knowledge in detail and follow high standards of logical reasoning while integrating the known and the new. This article describes an approach that utilizes the details in human biological pathways to identify pairs of changes with a likely cause-effect relation within. The approach automatically converts comparative proteomic and other molecular profiles into hypotheses of differentially active mechanistic relations that explain how the profiles came to be.

1 2 3 4 5 **Development/Pre-production:** Data science output has been rolled out/validated across multiple domains/problems

SUMMARY

We present a computational method to infer causal mechanisms in cell biology by analyzing changes in high-throughput proteomic profiles on the background of prior knowledge captured in biochemical reaction knowledge bases. The method mimics a biologist's traditional approach of explaining changes in data using prior knowledge but does this at the scale of hundreds of thousands of reactions. This is a specific example of how to automate scientific reasoning processes and illustrates the power of mapping from experimental data to prior knowledge via logic programming. The identified mechanisms can explain how experimental and physiological perturbations, propagating in a network of reactions, affect cellular responses and their phenotypic consequences. Causal pathway analysis is a powerful and flexible discovery tool for a wide range of cellular profiling data types and biological questions. The automated causation inference tool, as well as the source code, are freely available at http://causalpath.org.

INTRODUCTION

Central to a cell's decision-making processes is a vast network of biochemical reactions. A comprehensive, predictive model of cell biological mechanisms would revolutionize our scientific understanding and have tremendous clinical utility. Modeling efforts can be categorized roughly into two branches. The more established approach is to compile extensive,

This is an open access article under the CC BY license (http://creativecommons.org/licenses/by/4.0/).

interconnected pathway models through the curation of reactions based on carefully designed low-throughput controlled experiments. This classic approach led to the first large-scale metabolic maps and later was extended to signaling and transcriptional processes. Today this knowledge is represented in hundreds of pathway and interaction databases (pathguide.org). The newer, data-driven inference approach leverages the recent developments in proteomics and other molecular technologies to directly infer graphical models, *ab initio*, from high-throughput measurements of controlled perturbations and natural variation.[1–3]

Both the classic and the data-driven inference approaches have inherent limitations. The classic curation approach uses well-validated fragments of knowledge, but these are extracted from a heterogeneous set of contexts, perturbations, conditions, and even organisms. The resulting models, even when carefully restricted to a particular context, are not well suited to making predictions. The purely data-driven inference approaches, on the other hand, create context-specific, predictive models, but they do not scale in terms of statistical power as the model space is exponentially larger than the observable space.

A strategy to alleviate the power issue of the data-driven approach is to get help from prior knowledge when the perturbations in the data are not sufficient to decide between alternative models.[4–10] The methods that use this strategy, however, use prior knowledge in a reduced form, such as simple interaction networks, omitting mechanistic details and their logical harmony with the new data. The more an experiment lacks extensive perturbations, the more it can benefit from prior knowledge. Considering that the vast majority of currently available proteomic experiments have either few perturbations (e.g., before/after a stimulation) or only uncontrolled variation (e.g., profiles from disease cohorts), it is very important that we use prior knowledge in its full potential. In this perturbation-poor setting, model-building activity is transformed into selecting parts of the prior knowledge that can best explain the shape of the data, which we call ‘‘pathway extraction.’’ Here, we present a pathway extraction method, CausalPath, which uses the rich semantics of curated pathway knowledge, including the type of mechanism, the direction, signs of effect, and post-translational modifications. The inferred mechanisms are falsifiable hypotheses that can be experimentally interrogated.

CausalPath maps proteomic profiles to curated human pathways from multiple resources that are integrated into the Pathway Commons database,[11] detects the potential causal links in the pathways between measurable molecular features using a graphical pattern search framework, and identifies the subset of the causal links that can explain correlated changes in a given set of proteomic and other molecular profiles. These explanations are presented as an intuitive network with links to the detailed prior knowledge models and the related literature to create a powerful exploration and analysis platform (Figure 1). This approach in some sense mimics a literature search of a biologist for relationships that explain relationships in his or her data. The method takes into account hundreds of thousands of curated mechanisms, which would be infeasible to do manually. We demonstrate the value of CausalPath on multiple publicly available datasets covering a wide range of scenarios and biological questions: in a set of time-resolved epidermal growth factor (EGF) stimulation experiments we detected EGFR activation with its signaling downstream of MAPKs, including feedback inhibition on EGFR; from ligand-induced and drug-inhibited cell-line experiments, we estimated the precision of CausalPath predictions; from CPTAC (Clinical Proteomic Tumor Analysis Consortium) protein mass spectrometry datasets for ovarian and breast cancer we elucidated general and subtype-specific signaling, as well as regulators of well-known cancer proteins; and in RPPA (Reverse Phase Protein Array) experimental datasets of 32 TCGA (The Cancer Genome Atlas) cancer studies we found a core signaling network that is recurrently identified across many cancer types. These models bring new insights into cancer biology in terms of differences and commonalities of cancers in signaling. CausalPath is freely available to researchers through its website at causalpath.org for analysis of new proteomic experiments.

RESULTS

Design and properties of CausalPath

The CausalPath workflow has two main steps: (1) detection of causal priors from pathway databases, performed once and reused in multiple analyses, and (2) matching causal priors with supporting correlated changes in the analyzed data, performed for every analysis. We define a ‘‘causal prior’’ as a set of prior knowledge that as a group suggests a possible causal link between two measurable molecular features.

Existing kinase-substrate databases and transcription factor-target databases are valuable sources for causal priors, but they capture only a small part of the known biology; hence, they are limited for comprehensive causal reasoning. There are other databases that take a more detailed modeling approach for biochemical processes, such as Reactome. The Pathway Commons database provides integration of such detailed models collected from publicly available resources in the format of the BioPAX modeling language. Such models include details like post-translational modifications, molecular complexes, abstractions such as homologies, involvement of small molecules in signaling, and so on. Detailed process models provide a great opportunity to identify causal relations between the molecular measurements, but they require sophisticated algorithms to reason over them.

To detect the causal prior relations, i.e., structures that imply causal relationships between proteins in the Pathway Commons database, we used the BioPAX-pattern software[12], and manually curated 12 graphical patterns (described in Data S1). Each graphical pattern captures the control mechanisms over either a phosphorylation of a protein or the expression of a gene. Searching for these patterns in Pathway Commons generated 28,517 prior relations in four different types (listed below). To increase coverage, we added relations from several other databases (PhosphoNetworks,[13] iPTMnet,[14] TRRUST,[15] and TFactS),[16] which are not in Pathway Commons, and increased our relationships to 39,232:

CellPress
OPEN ACCESS

Relation type	Extracted from Pathway Commons	After addition	Details
Phosphorylation	20,020	24,430	from 2,230 proteins to 3,356 targets
Dephosphorylation	2,766	2,766	from 925 proteins to 338 targets
Expression upregulation	4,921	9,032	from 1,558 proteins to 1,915 targets
Expression downregulation	810	3,004	from 875 proteins to 1,018 targets

The imbalance in the number of relations reflects the representation bias of these relationship types in the scientific literature. We assessed the overlap of these prior relations with the "canonical pathways" gene sets in MSigDB to understand its coverage. This collection has 2,815 gene sets curated from the databases BioCarta, KEGG, NCI-PID, Reactome, and WikiPathways. The genes in our prior relations have a nonzero overlap with 99% of these gene sets. If we redefine "overlap" focusing on relations instead of genes, and require that both the source and the target gene of a relation be in a gene set to count as overlap, then our prior relations have nonzero overlap with 68% of the canonical pathway gene sets.

We define a "causal conjecture" as a pairing of a causal prior with supporting measurements in the molecular dataset that together declare that "one molecular change is the cause of another molecular change." The *change* here can be detected in two different forms based on the experimental setting: it can be up/downregulation for individual features in a "test versus control" comparison setting, or it can be positive/negative correlations applying to pairs of features in an uncontrolled study, as is common in cancer biology. We call an analysis in the former setting "comparison-based" and the latter "correlation-based." As an example of comparison-based generation of a causal conjecture, consider the following chain of items from a study that detects a set of proteomic changes after stimulation by EGF:

1. GAB1-pY406 peptide level is increased in response to EGF stimulation.	(from data)
2. Y406 is an activating phosphorylation site of GAB1.	(from prior knowledge)
3. GAB1 is part of a complex that can phosphorylate MAPK3 at Y204.	(from prior knowledge)
4. The MAPK3-Y204 peptide level is increased in response to EGF stimulation.	(from data)

Items 1 and 4 are direct observations from proteomic profiles and they are observed within the EGF stimulation context, and items 2 and 3 are the knowledge fragments that constitute the causal prior, as reported in publications from other experiments and subsequently curated into pathway databases. The causal conjecture here is that the increase in phospho-GAB1 (GAB1-pY406) after EGF stimulation causes an increase in its activity of helping phosphorylation of MAPK3 and hence an increase in the level of MAPK3-Y204. This is a well-defined, mechanistic, and falsifiable conjecture that is easily testable by perturbations (see Figure S1 for a complete iteration of different forms of causal conjectures used in this study). The important aspect here is that this conjecture is automatically generated, rather than inferred by a researcher.

In the case of a correlation-based analysis, we replace items 1 and 4 with an observed correlation, e.g., "Measured peptide levels of GAB1-pY406 are positively correlated with the peptide levels of MAPK3-Y204," for a *correlation-based* causality hypothesis.

$$\overline{\mathbf{c}_{\mathbf{source}} \oplus \mathbf{e}_{\mathbf{source}} \oplus \mathbf{s}_{\mathbf{relation}} \oplus \mathbf{c}_{\mathbf{target}}} = \mathbf{true} \quad (\text{comparison-based}) \tag{Equation 1}$$

$$\mathbf{corr}_{\mathbf{source,target}} \oplus (\mathbf{e}_{\mathbf{source}} \oplus \mathbf{s}_{\mathbf{relation}}) = \mathbf{true} \quad (\text{correlation-based}) \tag{Equation 2}$$

To formalize and generalize the example of causal conjecture detection in comparison-based analysis, we can formulate it with a ternary logical equation (Equation 1), where $\oplus$ is a ternary XOR operation, the overline is logical negation, *c* represents the change direction of the gene features, *e* represents the effect of the source feature on its activity, and *s* represents the sign of the pathway relation, where $c, e, s \in \{true, false, unknown\}$. The four terms in the equation correspond to the four items in the example, which collectively test if the data are consistent with a known causal interaction. The change of gene features are *true* in the case of upregulation, *false* in the case of downregulation, and *unknown* in the case of insignificant. The effect of source feature e_{source} is *true* in the case of total protein or activating phosphorylation, *false* in the case of inactivating phosphorylation, and *unknown* if it is a phosphorylation site with unknown effect. The relation sign, $s_{relation}$, is *true* for phosphorylation and expression upregulation and *false* for dephosphorylation and expression downregulation. Any $\oplus$ operation on *unknown* value will yield an *unknown* result, hence the equation does not hold if any value is *unknown*. In the case of correlation-based causality, instead of the terms c_{source} and c_{target}, we use the logical representation of the sign of the correlation ($corr_{source,target}$), where *true* represents positive correlation and *false* represents negative correlation (Equation 2). In addition to the logical check by these equations, we limit the phospho regulations (phosphorylation and dephosphorylation) to the explanation of phosphoprotein changes and limit the expressional regulations to the explanation of total protein changes (and optionally mRNA changes).

On top of the logic-based detection of causal interactions, we provide two types of statistical measurements to increase the interpretability of the results. "Network-size test" checks if the correlated changes align with the causal priors in general, which is indicated by a larger number of interactions in the results than would arise by random chance, which we test by data label randomization. "Downstream-size test" checks if a protein on the network has more downstream targets in the results than expected by chance using the same randomization procedure. Significant values from these two tests provide additional evidence suggesting the data are shaped by the priors or that a protein has an influence on the significant number of targets, respectively, which consequently increases our confidence in the results.

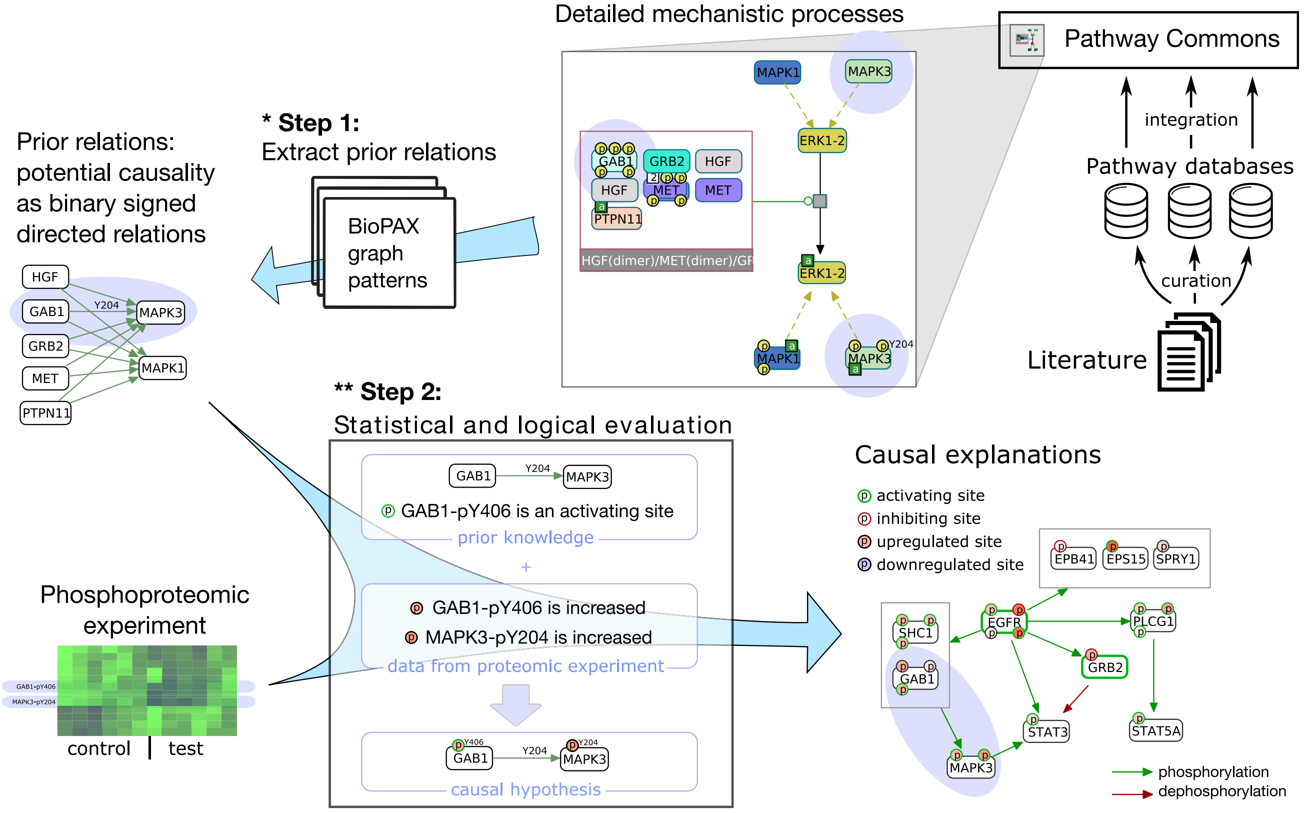

Figure 1. Overview of CausalPath pipeline over an example analysis

One relationship CausalPath generated from the EGF stimulation study was GAB1 → MAPK3. Prior information for this relationship was curated into pathway databases, which we integrate into Pathway Commons as detailed mechanistic processes. We detect structural patterns in these processes that indicate that GAB1, when activated through phosphorylation, can, in turn, help in phosphorylation of MAPK3 (step 1). These phosphorylations were correlated in the proteomics dataset in the direction compatible with the prior, so CausalPath selects this relationship as a potential explanation (step 2). The final logical network is a subgraph of the EGF stimulation analysis results at 2 min time frame. For a more comprehensive description of graph notation, please see Figure 3C. We omit phosphorylation site locations while rendering the resulting network for complexity management; these can be inspected interactively within the CausalPath on causalpath.org. (This figure provides conceptual examples for steps 1 and 2 of CausalPath. *Step 1 recognizes a variety of pathway structures that can causally link an upstream protein activity to a downstream proteomic feature, which are detailed in Data S1. **Step 2 checks if the direction of the measured proteomic changes is compatible with the expectations set by the prior information using Equations 1 and 2 [see main text]. Step 2 is demonstrated in more detail in Figure S1.)

Testing and validation of the method

We performed three studies to evaluate the method's performance and understand its characteristics: (1) To demonstrate the method on a simple test case, we reanalyzed proteomic profiles from an EGF stimulation experiment. (2) We measured the precision of CausalPath results, where we analyze ligand stimulation of four different breast cancer cell lines and test the predictions with protein inhibitors. (3) Using a proteomic experiment that measures the effects of 31 different drugs on PC3 cell lines, we measured CausalPath's ability to relate observed changes to altered drug targets.

We provide analyses for the robustness and reproducibility of CausalPath results, as well as a survey of other methods related to pathway analysis for proteomic datasets in Data S1.

Analysis of EGF stimulation on EGFR Flp-in cells

We reanalyzed a recent cell-line EGF stimulation phosphoproteomic dataset[5] to see if CausalPath can identify downstream events of EGF signaling. The experiment provides mass spectrometry profiles at eight time points, where a total of 1,068 phosphopeptides are measured. We compared each time point with the initial time point (unstimulated cells) to see how the EGF stimulus is propagated over time. Since the data are phosphopeptide only and do not contain any observable change on EGF itself, we included EGF activation as a "hypothesis" to the analysis. At the initial time points, CausalPath detects many EGFR targets and relates them to EGFR phosphorylation and activation. As one expects, both EGF and its receptor EGFR downstream are significantly enriched with changes that indicate their activation. At the fifth time point (16 min), we observe inhibitory feedback phosphorylation of EGFR explainable by MAPK1 and MAPK3 activity, followed by the dramatic dampening of EGF signaling. All the networks up to the fifth time point are significant in size ($p < 0.0001$).

Interestingly, an explanation for MAPK1/3 phosphorylation is missing in this result. It is known that EGF signaling can activate MAPK1/3 through several steps and multiple paths, but none was captured. In its most strict configuration, CausalPath forces phosphorylation sites in the literature to exactly match the detected sites in the phosphoproteomic data. When we slightly relax this constraint by allowing two amino acids difference in site locations, we detect that SHC1 and GAB1 phosphorylations can causally link EGF stimulation to MAPK3 phosphorylation

CellPress
OPEN ACCESS

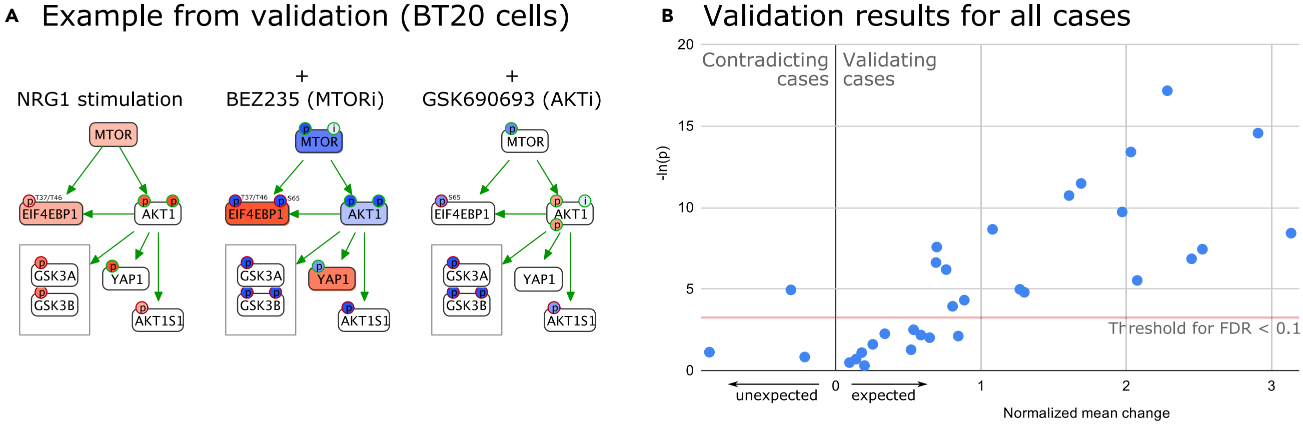

Figure 2. Validation of CausalPath relations on a cell-line ligand-stimulation and drug-inhibition RPPA dataset

(A) An example subnetwork from CausalPath results to illustrate how the validation works. The first subnetwork is generated by comparing NRG1-stimulated BT20 cells with the unstimulated control cells. Since this network nominates activated MTOR and AKT1 as the cause of several downstream phosphorylations, we can test these hypotheses using MTOR and AKT inhibitors. The next two graphs show the same subnetwork after the inhibitors are applied (ligand+/inhibitor+ cells are compared with ligand+/inhibitor− cells). See Figure 3C for graph legend.

(B) Cumulative validation results from all 32 cases, generated by readouts from 14 distinct antibodies. The x axis has mean changes in the antibody readouts normalized to their global standard deviation and expected direction. A positive value indicates the change is in the expected direction.

(Video S1). Site locations reported in the literature are sometimes shifted relative to the sequence of the canonical protein isoform provided by UniProt. For example, it is relatively common in the literature to omit the initial methionine on the protein, which is often cleaved, but UniProt uniformly includes these methionines in its reference sequence. We are actively working on curation-correction tools for addressing these problems in the future. As a stop-gap measure, CausalPath's option to slightly relax the site matching is useful for most applications.

Precision of CausalPath results on cell lines stimulated with ligands

We used a recently published RPPA experiment to estimate the precision of CausalPath results. This experiment stimulates four different breast cancer cell lines with seven different ligands, and also treats each cell line/ligand combination using five targeted drugs.[17]

Cell lines	Ligands	Inhibitor drugs
BT20	EGF	AZD8055 targeting MTOR
BT549	FGF1	BEZ235 targeting PIK3CA and MTOR
MCF7	HGF	GSK690693 targeting AKT
UACC812	IGF1	GSK1120212 targeting MEK
	insulin	PD173074 targeting FGFR
	NRG1	
	PBS*	

*The case of PBS represents the lack of ligands that are naturally found in bovine serum that is used as control in all cases.

We first ignored the drug inhibition samples, and used the ligand stimulation experiments to predict their associated causal relations using CausalPath. Then, we identified the relations in the result networks whose source protein is targeted by one of the inhibitor drugs in the study. For these relations, the inhibition experiments provide validation of their inference. When the drug targeting the upstream protein is applied, if the CausalPath result relation is valid, we expect to see a reverse change downstream of the drug targets (Figure 2A). We identified a total of 32 CausalPath relations in the results that are verifiable by analyzing the existing inhibition experiments. We found that in 29 of the 32 cases the antibody readout changes in our predicted direction, and 3 of them change the other way, suggesting a precision of 0.91 without considering the significance of the change (Figure 2B and Table S1). Nineteen of those changes are statistically significant, with a 0.1 false discovery rate (FDR) cutoff; 18 of these are in the expected direction, validating the CausalPath result, and only one is contradicting. If we assume all insignificant cases are *unchanged*, then the precision estimate drops to 0.56. In reality, the insignificant results are a mix of changed and unchanged, because we chose a threshold to have a reasonably low false positive rate at the expense of a possible high false negative rate. To estimate the false negative rate, we assume that the noise is symmetrically distributed around 0 and assume all three cases on the negative side are noise, predicting three additional unchanged cases on the positive side. This brings the estimate of changed-in-expected-direction cases to 26, suggesting a more realistic precision estimate of 0.81. This level of precision is very reasonable for most studies to justify following up with experimental verification.

Analysis of PC3 cell line drug perturbations

To evaluate CausalPath on a series of perturbations systematically, we analyzed the mass spectrometry data from a recent set of drug perturbation experiments on PC3 prostate cancer cell lines where a total of 3,979 phosphopeptides were measured.[18] For this analysis, we collected known targets of the drugs from the literature and inserted the inactivation of these targets as custom hypotheses. We found that for 14 of the 31 drugs CausalPath can identify proteomic changes that can be explained by inhibiting the drug's known target (Table S2). For

four of these drugs, CausalPath detects enrichment downstream of the drug's targets, indicating its inactivation. In other words, even if we do not insert custom hypotheses for known drug targets, CausalPath can correctly predict the targets of these four drugs by evaluating changes in their downstream proteins. These drugs and their identified targets are afuresertib (AKT1), dinaciclib (CDK1, CDK2), flavopiridol (CDK1, CDK2, CDK6), and staurosporine (CDK2, MAPKAPK2) (Table S2). The results indicate that whenever a drug targets CDK1/CDK2 on PC3 cells (three of the drugs in the study), CausalPath can identify it using the downstream-size test. This implies that CDK1/2 activity is playing an important role in PC3 biology, a relatively large number of its targets respond to its inactivation, and also their relations are relatively well modeled in pathway databases.

Analysis of the CPTAC ovarian cancer dataset

Four hundred eighty-nine high-grade serous ovarian cancer (HGSOC) samples were previously profiled by the TCGA project.[19] A recent CPTAC project performed proteomic and phosphoproteomic analysis on 174 of the original TCGA ovarian cancer samples using mass spectrometry, providing measurements for 9,600 proteins from the 174 samples and 24,429 phosphosites from 6,769 phosphoproteins from 69 samples.[20]

Using CausalPath on this dataset, we generated explanations for the observed correlations in the measured peptide levels, using phosphorylation and expression regulation pathway relations. The first case explains phosphopeptide changes through phospho relations, and the second case explains total protein changes through expression regulation relations. In both cases, the upstream "cause" in the explanations is either a total protein or a phosphoprotein change. The resulting phosphorylation network contains 139 relations and the expression network contains 243 relations when we use a 0.1 FDR threshold for correlations. Interestingly, while the size of the phosphorylation network is significantly large ($p < 0.0001$, calculated by data label randomization), we do not observe this for the expression network ($p = 0.6283$). The most notable parts of the phosphorylation network include CDK1 and CDK2 downstream, MAPK1 and MAPK3 downstream, and several immune-related proteins such as SRC family kinases, PRKCD, and PRKCQ (Figure 3A).

Potential reasons for the radically different significance values for expression-regulation relations compared with phosphorylations include lower quality of expression regulation priors, higher number of confounding factors, and the relatively weak correlation between total protein measurements and their corresponding RNA expression. To investigate this, we modified CausalPath to use TCGA RNA-sequencing (RNA-seq) data instead of proteomic data for the target genes of expression regulation controls. We obtained 192 expression regulations that explain RNA measurements of 140 genes with proteomic changes of 92 transcription factors or their modulators. The size of the resulting network became significant after this change ($p < 0.0001$), confirming that the proteomic change is not a very good proxy for RNA expression (and vice versa). In addition, the downstream changes in four transcription factors (STAT1, NFKB1, MCM6, and SPI1) are significantly large (0.1 FDR), suggesting that these factors are significant sources of variance in ovarian cancer.

The correlation-based causal network provides hypotheses for the signaling network parts that are differentially active across samples, but it does not indicate which parts are activated together or whether they align with previously defined molecular subtypes. The original TCGA study on HGSOC samples identifies four molecular subtypes based on RNA expression, termed as immunoreactive, differentiated, proliferative, and mesenchymal.[19] To understand if we can gain mechanistic insight into the previously defined subtypes, we compared each subtype to all other samples using a t test with Benjamini-Hochberg FDR control on measurements, but we were unable to generate substantial results within a 0.1 FDR threshold, probably due to the large proportion of missing values in the phosphoproteomic dataset combined with the loss of statistical power due to smaller cohort size for each subtype. Then we tried to constrain the search space with the neighborhoods of some of the genes with differential measurements, and relax the FDR threshold at the same time for further exploration. Six SRC family kinases (SFKs) have proteomic evidence for activation in the immunoreactive subtype; hence, we limited the search to the neighborhood of SFKs (SRC, FYN, LYN, LCK, HCK, and FGR), set the FDR threshold to 0.2 for phosphoproteomic data, and identified 27 relations (Figure 3B). The network identifies several human leukocyte antigen system (the major histocompatibility complex in humans) proteins at SFK upstream, along with other genes regulating immune cell activation, such as CD4, ITGA4, PTPRC, PTPRJ, PTPN1, and NCK1. On the network, we identify a signal transmitted from SFKs to CD247 and FCER1G, immune response genes.

Analysis of the CPTAC breast cancer dataset

Another CPTAC project produced proteomic and phosphoproteomic profiles for 105 of the original TCGA breast cancer samples with mass spectrometry,[21] where 77 of the samples were tagged by the authors as being high quality and were used in this study. Unlike the ovarian cancer dataset, this dataset is rich in correlations, which can be explained by 1,756 phospho regulations and 488 expression regulations. The resulting phosphorylation network has 11 significant proteins (PRKD1, CDK2, DYRK1B, PPP2CA, MAPKAPK2, PPP2CB, RPS6KA3, PRKDC, AKT1, SHC1, and IKBKE) with an enriched downstream (Figure 4A). These enriched proteins all have established functions in breast cancer literature—maybe with the exception of DYRK1B, whose high expression was only recently associated with worse prognosis in breast cancer,[22] potentially because its inhibitory effect on the cell cycle rescues breast cancer cells from apoptosis and cytotoxic drugs.[23]

Similar to the ovarian cancer results, we detected that the breast cancer phosphorylation network is significant in size ($p < 0.0001$), while the expression network is not ($p = 0.5521$), suggesting that the known phosphorylation relations have a much higher impact on the proteomic correlations than known expressional relations. When we use TCGA RNA-seq data instead of proteomic data for the targets of expression regulation, we detect 248 relations that explain RNA changes in 155 target genes by proteomic changes of 120 transcription factors or their modulators. With RNA-seq data, the size of the network is highly significant ($p < 0.0001$), with 3 transcription factors

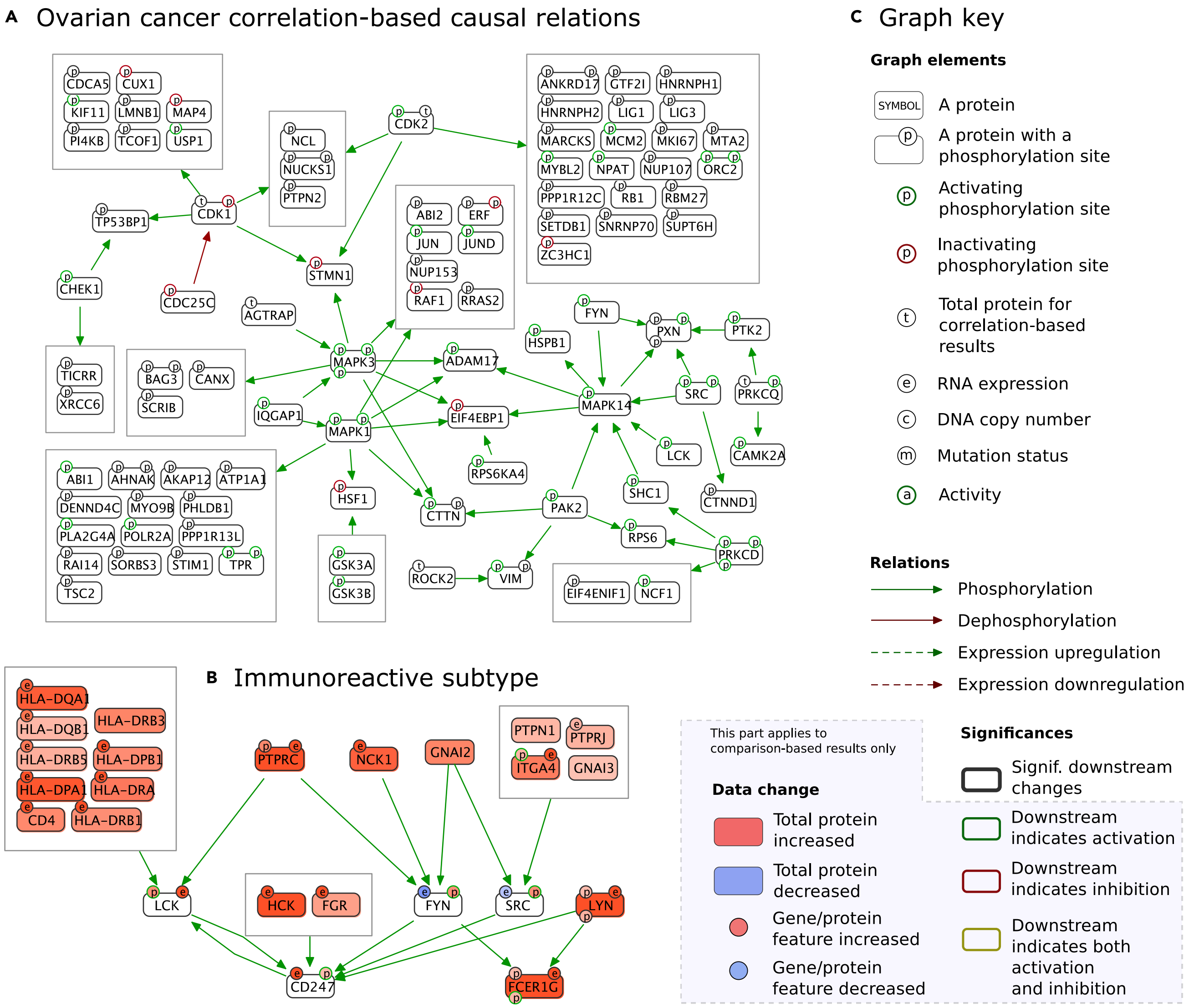

Figure 3. Results for CPTAC ovarian cancer

(A) The largest connected component in the correlation-based causality network with phospho regulations. Note that the visual notation of this correlation-based result network is different from that of the comparison-based network in Figure 1, as we have no differential comparison but have pairwise correlations. For a compiled set of examples on how to read parts of a CausalPath result graph, please see Figure S4.

(B) Immunoreactive subtype compared with all other samples, where we show RNA expression and DNA copy variation from corresponding TCGA datasets along with the CPTAC proteomic changes.

(C) Key for the graph notation for causal explanations in all figures.

(GATA3, STAT1, and ESR1) having correlated targets enriched in the results (Figure 4B).

Next, we compared the PAM50 expression subtypes of breast cancer to see if we could get causal explanations of their proteomic differences. We were again challenged by decreased sample sizes and missing values, but we detected that luminal A and luminal B subtypes have significant differences from the basal-like subtype. This time, CausalPath results were not significant in terms of the overall network size (p = 0.2218); nevertheless, they indicate that ESR1 is significantly more active in luminal breast cancers, suggested by both its protein levels and the changes in its downstream (Figure 4C). Transcriptional downstream of ESR1 captures other important elevated transcription factors functioning in the luminal subtypes, such as FOXA1, AR, and PGR. AR is an emerging target in breast cancer.[24]

Analysis of TCGA RPPA datasets

There are 32 TCGA studies that provide proteomic and phosphoproteomic measurements of tumor biopsies from various types of cancer patients. Those studies provide RPPA profiles of a total of 7,694 patients using 259 antibodies. The low number of protein measurements in the datasets prevents a comprehensive pathway analysis, but the antibodies are selected for the

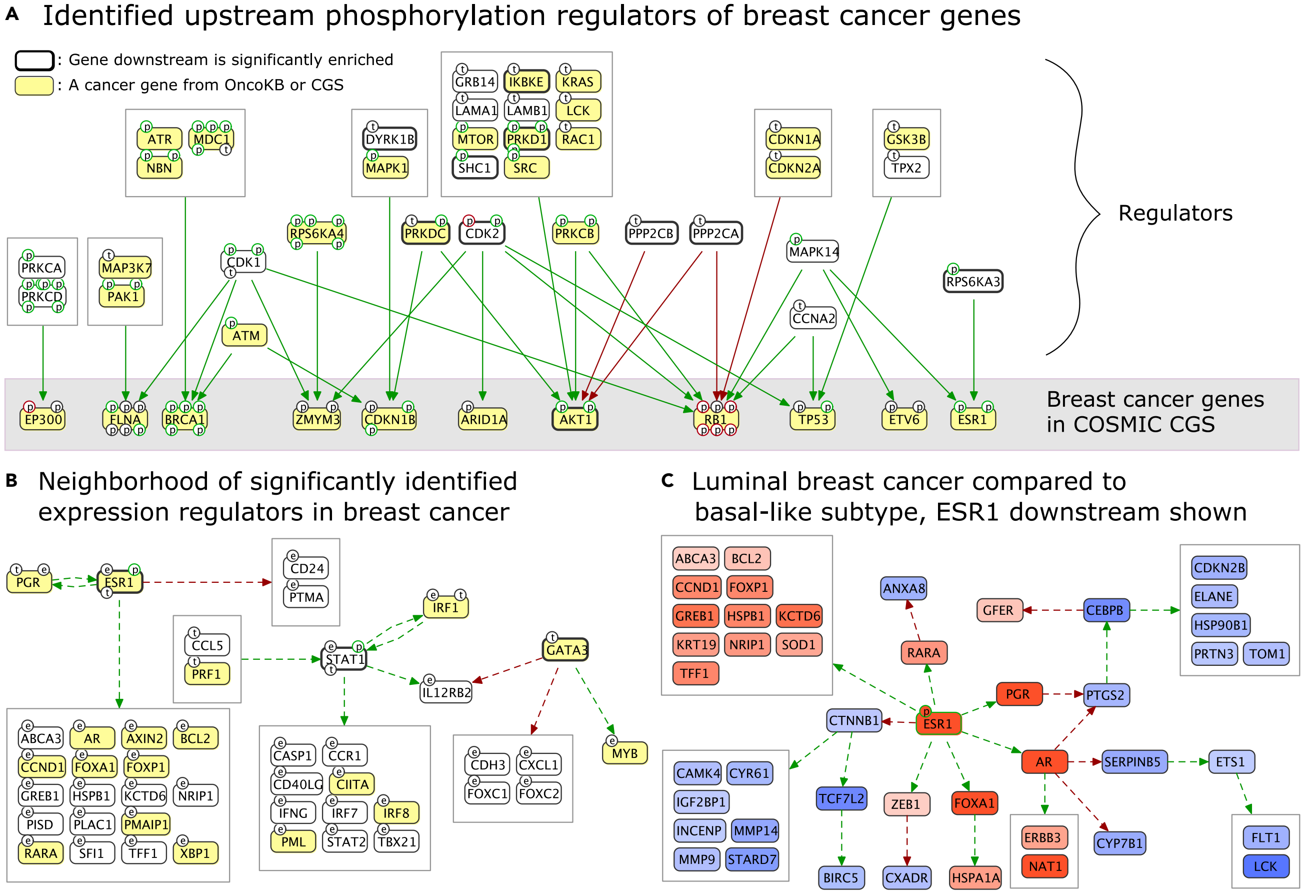

Figure 4. Results for CPTAC breast cancer

(A) A subgraph of the correlation-based causal network with phospho regulations focused on the upstream regulators of proteins that are implicated in breast cancer as provided by the COSMIC Cancer Gene Census (CGS) database. There are 43 genes in CGS annotated with breast cancer, for 11 of which we identify phosphorylation regulators.

(B) Subgraph of the correlation-based causal network with expression regulations where RNA-seq changes are explained by upstream proteomic changes, focused on the neighborhood of proteins with significant downstream.

(C) Luminal A and luminal B subtypes are collectively compared with the basal-like subtype. Only the ESR1 downstream relations are shown.

proteins' relevancy to cancer in general, and they are typically well studied with many established relations between them. We sought to determine which of these relations most frequently have evidence in the form of correlation across cancer types. We generated a correlation-based causal network for each cancer type using a strict FDR threshold of 0.001, then we ranked these relations according to how many cancer datasets they can explain (Figure 5 and Table S3). We found that AKT to GSK3 signaling is the most frequently observed relation, detectable in 30 cancer types, followed by other downstream proteins of AKT, including MTOR. Relations between several MAPK signaling proteins and EGFR to ERBB2 signaling are also among those observed in the vast majority of cancer types. It is important to note that the results do not indicate that these signaling paths are almost always active in cancers, but they indicate that there is a high patient-to-patient variation in their activity, almost always, making them relevant for precision medicine. This is consistent with many studies reporting the AKT pathway as a major resistance mechanism to chemotherapy and some other targeted therapies.[25–27]

DISCUSSION

Pathway extraction versus pathway inference

CausalPath is a novel pathway extraction method to aid researchers in understanding experimental observations using known mechanisms with a focus on post-translational modifications. Experimental data reveal protein features that change in coordination, and CausalPath automates the search for causal explanations in the literature. Loosely speaking, context-specific correlations are derived from the data and causality is derived from the literature. Compared with the methods that infer causality from data through mathematical modeling (pathway inference), this method has a much wider application area. Pathway inference methods have a potential to offer more complete results, but they require numerous perturbations and/or time points in the experiments, whereas the pathway extraction strategy is applicable to any simple comparison, or a set of profiles from a cohort with some variance to explain. This is extremely important, since even with the large CPTAC datasets, we are still data limited, especially when we try to understand subtype-

CellPress
OPEN ACCESS

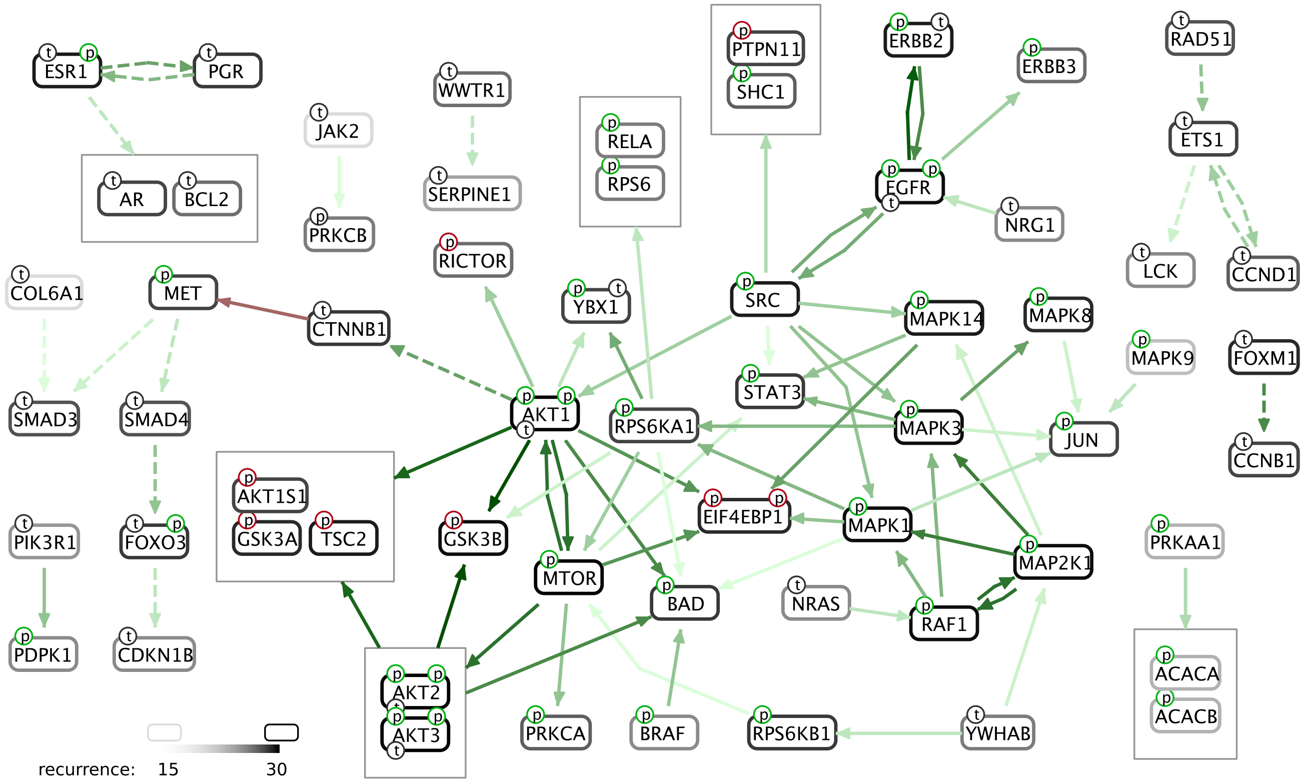

Figure 5. Recurrent results for TCGA RPPA datasets

Relations that are identified with correlation-based analysis in at least 15 cancer types are shown, where faintest color indicates 15 and boldest color indicates 30. Please note that the bold node borders are repurposed in the graph notation to display recurrence rate.

specific mechanisms. We believe that we will see parallel progress in both strategies as the data and knowledge increase respectively with a potential convergence in the future. In addition, CausalPath is a great resource for high-confidence *priors*, which can inform the hybrid pathway inference methods that benefit from prior data.

Novelty in pathway extraction

Even though pathway extraction cannot hypothesize the existence of new relations that were never seen before in any context, its results cannot be dismissed as not novel. Existing relations in pathway databases are collected from diverse contexts, cell types, disease models, etc. For a new context of focus, it is very challenging to identify which of the previously described relations are applicable. Causality-focused pathway extraction approaches provide a means of transferring knowledge between contexts. CausalPath does this by detecting variation patterns of proteomic abundances and detecting their consistency with prior knowledge. A limitation of this approach is its dependency on observable variance; therefore, it cannot identify a signaling relation that does not significantly vary across the compared samples.

The added value and future challenges

The added value that our method brings to the field of pathway extraction is three-fold: (1) interpretation of complex mechanistic pathway models, (2) site-specific evaluation of phosphoproteomic measurements, and (3) a logical test for causality between measurements. When all these are combined, pathway extraction becomes a useful tool for "mechanistic model building." We expect future research will take these ideas further, potentially addressing these two challenges: (1) instead of a binary evaluation of causality (between two proteins), n-ary systems can be developed, and (2) instead of a binary classification of protein modifications as activating and inhibiting, a site can be more accurately mapped to a distinct subset of activities of the protein. These challenges can be tackled gradually as we have more detailed and more complete models of cellular processes in pathway databases.

Proteomic versus transcriptomic level events

Our results show that evidence of known phospho regulations is more consistently observed in the proteomic data compared with the known expression regulations. In the ovarian and breast cancer datasets, the sizes of the resulting phosphorylation networks are significantly higher compared with background, while expression networks remain similar. In the recurrence study with TCGA RPPA datasets, 34% of the resulting phosphorylation controls recur in at least 15 cancer types (Figure S2). This ratio is only 7% for expression level controls. This is perhaps expected, as a phosphorylation relation can directly explain a phosphopeptide change, while an expression event can only indirectly explain a total protein change requiring the mRNA level of the target to be highly correlated with its protein abundance. While there is definitely an overall correlation (Figure S3), it is

not high enough to use protein data as a reliable proxy for mRNA in general. However, there are exceptions, for example, we could identify ESR1 differential activity in luminal breast cancers purely from proteomic data, using expression relations. Based on these observations, CausalPath lets users select the molecular data type to use for targets of expression relations.

Missing or flawed pathway relations

One major limiting factor in this analysis is a large number of protein phosphorylation sites whose functions are not known; hence, their downstream cannot be included in the causality network. We are actively working to mine these data from the literature using natural language processing tools.[28] In the meantime, CausalPath reports those sites with an unknown effect that also have significant change at their signaling downstream. Users have the option to review this list of modification sites and manually curate them to increase the coverage of the analysis.

Rarely, in the causality analysis results, we encounter relations that are erroneous. These are generally results of manual curation issues. In these cases, we report them to the source databases, and we remove these erroneous pathway interactions from our network so that future analyses are not affected. We encourage researchers to report such errors to source databases (or alternatively to us), if they come across any, to improve the accuracy of our collective knowledge of biochemical pathways. We are actively working on a collaborative data-explaining platform that will further streamline the curation and error reporting steps.

Recommendation for use

CausalPath can be applied to the results of any proteomic and phosphoproteomic experiments to identify differential signaling that is supported by literature knowledge. To use CausalPath, the measurement values need to be comparable (normalized) and need to be associated with related gene symbols, and phosphopeptide measurements need to specify the phosphorylation sites with respect to their canonical UniProt sequence, in a special format that is described at the website, causalpath.org. Users can either use the website to execute the analysis or run the analysis locally using CausalPath's open-source Java code. The result networks can be visualized using the viewer embedded in the CausalPath website or by loading to the pathway visualization tools ChiBE[29,30] or Newt.[31]

EXPERIMENTAL PROCEDURES

Resource availability

Lead contact

Further information and requests for digital resources should be directed to and will be fulfilled by the lead contact, Özgün Babur (ozgun.babur@umb.edu).

Materials availability

This study did not generate new unique reagents.

Data and code availability

CausalPath is freely available at http://causalpath.org. Users can upload the proteomic data in a tab-delimited format, along with the analysis parameters, such as how to detect a *change* in the values. Options include averaging a group of values, getting difference/fold-change of two groups of columns, comparing two groups with a t test, or using correlations in a single group. The results are visualized as an interactive network using Cytoscape.js,[32] and the mechanistic details of each interaction can be viewed in SBGN-PD language[31] using a layout algorithm specifically designed for compound graph structures.[33] Alternatively, CausalPath can be run locally as a Java application using the sources at https://github.com/PathwayAndDataAnalysis/causalpath. This repository additionally includes examples running CausalPath from R and Python.[34] The generated result networks can be visualized with ChiBE,[29,30] as well as by uploading analysis output folders to the CausalPath web server at causalpath.org. All network figures in this article were generated with ChiBE.

The results presented in this article can be reproduced using the datasets, parameters, and software in the supplementary archive at https://www.synapse.org/#!Synapse:syn17014378. An alternative URL for this archive is https://doi.org/10.5281/zenodo.4477801.

SUPPLEMENTAL INFORMATION

Supplemental information can be found online at https://doi.org/10.1016/j.patter.2021.100257.

ACKNOWLEDGMENTS

We thank Hannah Manning and Olga Nikolova for critical reading of the manuscript. This work was sponsored by DARPA under the Big Mechanism Program (contract W911NF-14-C-0119) and the U.S. Army Research Office (contract ACC-APG_RTP W911NF), and by NIH grants R01HL146549 (to J.E.A.), U41HG006623 (Pathway Commons), and P41GM103504 (National Resource for Network Biology). A.K. is supported by MD Anderson Cancer Center support grant P30 CA016672 (Bioinformatics Shared Resource) and by an OCRA collaborative research grant. U.D. is supported by the Scientific and Technological Research Council of Turkey (grant 118E131). The results published here are in part based upon data generated by the Clinical Proteomic Tumor Analysis Consortium (NCI/NIH) and TCGA Research Network: http://cancergenome.nih.gov/.

AUTHOR CONTRIBUTIONS

Ö.B. and A.K. conceived the idea. Ö.B. designed and developed the method and performed all the analyses with help from A.K., A.L., J.E.A., and U.D. F.D. designed and developed the web service with help from M.C.S. and U.D. A.S.V.J., R.P., and K.E.C. contributed to the analysis of the PC3 drug perturbation dataset under the supervision of J.D.J. P.T.S. supervised the validation study on breast cancer cell lines. E.D. and C.S. supervised the project. Ö.B., E.D., A.L., C.S., and J.E.A. wrote the manuscript with help from F.D., U.D., and A.K.

DECLARATION OF INTERESTS

The authors declare no competing interests.

Received: October 1, 2020
Revised: November 10, 2020
Accepted: April 9, 2021
Published: May 12, 2021

REFERENCES

1. Molinelli, E.J., Korkut, A., Wang, W., Miller, M.L., Gauthier, N.P., Jing, X., Kaushik, P., He, Q., Mills, G., Solit, D.B., and Pratilas, C.A. (2013). Perturbation biology: inferring signaling networks in cellular systems. PLoS Comput. Biol. *9*, e1003290.
2. Hill, S.M., Heiser, L.M., Cokelaer, T., Unger, M., Nesser, N.K., Carlin, D.E., Zhang, Y., Sokolov, A., Paull, E.O., Wong, C.K., et al. (2016). Inferring causal molecular networks: empirical assessment through a community-based effort. Nat. Methods *13*, 310.
3. Triantafillou, S., Lagani, V., Heinze-Deml, C., Schmidt, A., Tegner, J., and Tsamardinos, I. (2017). Predicting causal relationships from biological data: applying automated causal discovery on mass cytometry data of human immune cells. Sci. Rep. *7*, 12724.

CellPress
OPEN ACCESS

4. Korkut, A., Wang, W., Demir, E., Aksoy, B.A., Jing, X., Molinelli, E.J., Babur, Ö., Bemis, D.L., Onur Sumer, S., Solit, D.B., et al. (2015). Perturbation biology nominates upstream–downstream drug combinations in raf inhibitor resistant melanoma cells. Elife *4*, e04640.

5. Köksal, A.S., Beck, K., Cronin, D.R., McKenna, A., Camp, N.D., Srivastava, S., MacGilvray, M.E., Bodík, R., Wolf-Yadlin, A., Fraenkel, E., et al. (2018). Synthesizing signaling pathways from temporal phosphoproteomic data. Cell Rep. *24*, 3607–3618.

6. Vaske, C.J., Benz, S.C., Sanborn, J.Z., Earl, D., Szeto, C., Zhu, J., Haussler, D., and Stuart, J.M. (2010). Inference of patient-specific pathway activities from multi-dimensional cancer genomics data using paradigm. Bioinformatics *26*, i237–i245.

7. Drake, J.M., Paull, E.O., Graham, N.A., Lee, J.K., Smith, B.A., Titz, B., Stoyanova, T., Faltermeier, C.M., Uzunangelov, V., Carlin, D.E., et al. (2016). Phosphoproteome integration reveals patient-specific networks in prostate cancer. Cell *166*, 1041–1054.

8. Melas, I.N., Samaga, R., Alexopoulos, L.G., and Klamt, S. (2013). Detecting and removing inconsistencies between experimental data and signaling network topologies using integer linear programming on interaction graphs. PLoS Comput. Biol. *9*, e1003204.

9. Terfve, C.D., Wilkes, E.H., Casado, P., Cutillas, P.R., and Saez-Rodriguez, J. (2015). Large-scale models of signal propagation in human cells derived from discovery phosphoproteomic data. Nat. Commun. *6*, 8033.

10. Chasman, D., Ho, Y.H., Berry, D.B., Nemec, C.M., MacGilvray, M.E., Hose, J., Merrill, A.E., Lee, M.V., Will, J.L., Coon, J.J., et al. (2014). Pathway connectivity and signaling coordination in the yeast stress-activated signaling network. Mol. Syst. Biol. *10*, 759.

11. Cerami, E.G., Gross, B.E., Demir, E., Rodchenkov, I., Babur, Ö., Anwar, N., Schultz, N., Bader, G.D., and Sander, C. (2011). Pathway commons, a web resource for biological pathway data. Nucleic Acids Res. *39*, D685–D690.

12. Babur, Ö., Aksoy, B.A., Rodchenkov, I., Sümer, S.O., Sander, C., and Demir, E. (2014). Pattern search in BioPAX models. Bioinformatics *30*, 139–140.

13. Hu, J., Rho, H.S., Newman, R.H., Zhang, J., Zhu, H., Qian, J., and Phosphonetworks. (2013). A database for human phosphorylation networks. Bioinformatics *30*, 141–142.

14. Ross, K.E., Huang, H., Ren, J., Arighi, C.N., Li, G., Tudor, C.O., Lv, M., Lee, J.Y., Chen, S.C., Vijay-Shanker, K., and Wu, C.H. (2017). iptmnet: Integrative bioinformatics for studying PTM networks. Protein Bioinformatics, 333–353.

15. Han, H., Cho, J.W., Lee, S., Yun, A., Kim, H., Bae, D., Yang, S., Kim, C.Y., Lee, M., Kim, E., et al. (2017). Trrust v2: an expanded reference database of human and mouse transcriptional regulatory interactions. Nucleic Acids Res. *46*, D380–D386.

16. Essaghir, A., and Demoulin, J.B. (2012). A minimal connected network of transcription factors regulated in human tumors and its application to the quest for universal cancer biomarkers. PLoS One *7*, e39666.

17. Hill, S.M., Nesser, N.K., Johnson-Camacho, K., Jeffress, M., Johnson, A., Boniface, C., Spencer, S.E., Lu, Y., Heiser, L.M., Lawrence, Y., et al. (2017). Context specificity in causal signaling networks revealed by phosphoprotein profiling. Cell Syst. *4*, 73–83.

18. Peckner, R., Myers, S.A., Jacome, A.S.V., Egertson, J.D., Abelin, J.G., MacCoss, M.J., Carr, S.A., and Jaffe, J.D. (2018). Specter: linear deconvolution for targeted analysis of data-independent acquisition mass spectrometry proteomics. Nat. Methods *15*, 371.

19. The Cancer Genome Atlas Research Network. (2011). Integrated genomic analyses of ovarian carcinoma. Nature *474*, 609–615.

20. Zhang, H., Liu, T., Zhang, Z., Payne, S.H., Zhang, B., McDermott, J.E., Zhou, J.Y., Petyuk, V.A., Chen, L., Ray, D., et al. (2016). Integrated proteogenomic characterization of human high-grade serous ovarian cancer. Cell *166*, 755–765.

21. Mertins, P., Mani, D., Ruggles, K.V., Gillette, M.A., Clauser, K.R., Wang, P., Wang, X., Qiao, J.W., Cao, S., Petralia, F., et al. (2016). Proteogenomics connects somatic mutations to signalling in breast cancer. Nature *534*, 55–62.

22. Chen, Y., Wang, S., He, Z., Sun, F., Huang, Y., Ni, Q., Wang, H., Wang, Y., and Cheng, C. (2017). Dyrk1b overexpression is associated with breast cancer growth and a poor prognosis. Hum. Pathol. *66*, 48–58.

23. Becker, W. (2018). A wake-up call to quiescent cancer cells–potential use of dyrk 1b inhibitors in cancer therapy. FEBS J. *285*, 1203–1211.

24. Kono, M., Fujii, T., Lim, B., Karuturi, M.S., Tripathy, D., and Ueno, N.T. (2017). Androgen receptor function and androgen receptor–targeted therapies in breast cancer: a review. JAMA Oncol. *3*, 1266–1273.

25. Cassinelli, G., Zuco, V., Gatti, L., Lanzi, C., Zaffaroni, N., Colombo, D., and Perego, P. (2013). Targeting the akt kinase to modulate survival, invasiveness and drug resistance of cancer cells. Curr. Med. Chem. *20*, 1923–1945.

26. Jacobsen, K., Bertran-Alamillo, J., Molina, M.A., Teixidó, C., Karachaliou, N., Pedersen, M.H., Castellví, J., Garzón, M., Codony-Servat, C., Codony-Servat, J., and Giménez-Capitán, A. (2017). Convergent akt activation drives acquired egfr inhibitor resistance in lung cancer. Nat. Commun. *8*, 410.

27. West, K.A., Castillo, S.S., and Dennis, P.A. (2002). Activation of the pi3k/akt pathway and chemotherapeutic resistance. Drug Resist. Updates *5*, 234–248.

28. Valenzuela-Escárcega, M.A., Babur, Ö., Hahn-Powell, G., Bell, D., Hicks, T., Noriega-Atala, E., Wang, X., Surdeanu, M., Demir, E., and Morrison, C.T. (2018). Large-scale automated machine reading discovers new cancer-driving mechanisms. Database *2018*, bay098.

29. Babur, O., Dogrusoz, U., Demir, E., and Sander, C. (2010). ChiBE: interactive visualization and manipulation of BioPAX pathway models. Bioinformatics *26*, 429–431.

30. Babur, Ö., Dogrusoz, U., Çakir, M., Aksoy, B.A., Schultz, N., Sander, C., and Demir, E. (2014). Integrating biological pathways and genomic profiles with ChiBE 2. BMC Genomics *15*, 642.

31. Sari, M., Bahceci, I., Dogrusoz, U., Sumer, S.O., Aksoy, B.A., Babur, Ö., and Demir, E. (2015). Sbgnviz: a tool for visualization and complexity management of SBGN process description maps. PLoS One *10*, e0128985.

32. Franz, M., Lopes, C.T., Huck, G., Dong, Y., Sumer, O., and Bader, G.D. (2015). Cytoscape. js: a graph theory library for visualisation and analysis. Bioinformatics *32*, 309–311.

33. Dogrusoz, U., Giral, E., Cetintas, A., Civril, A., and Demir, E. (2009). A layout algorithm for undirected compound graphs. Inf. Sci. *179*, 980–994.

34. Luna, A., Babur, Ö., Aksoy, B.A., Demir, E., and Sander, C. (2015). PaxtoolsR: pathway analysis in R using pathway commons. Bioinformatics *32*, 1262–1264.

Patterns, Volume *2*

Supplemental information

Causal interactions from proteomic profiles:

Molecular data meet pathway knowledge

Özgün Babur, Augustin Luna, Anil Korkut, Funda Durupinar, Metin Can Siper, Ugur Dogrusoz, Alvaro Sebastian Vaca Jacome, Ryan Peckner, Karen E. Christianson, Jacob D. Jaffe, Paul T. Spellman, Joseph E. Aslan, Chris Sander, and Emek Demir

Supplemental Information

Contents

1 Supplemental Experimental Procedures **2**
1.1 Significance for proteomic data change and correlation . . . 2
1.2 Causality . . . 2
1.3 Derivation of prior relations from detailed mechanistic pathways . . . 2
1.4 Algorithm for selection of explanatory subset of prior relations . . . 2
1.5 Description of causal graph notation . . . 3
1.6 CausalPath parameters . . . 4
1.6.1 Site matching proximity threshold . . . 4
1.6.2 Site effect proximity threshold . . . 4
1.6.3 Gene focus . . . 4
1.6.4 Generation of a data-centric causal network . . . 4
1.6.5 Protein activity . . . 5
1.6.6 Adjusting phosphopeptide measurements with total protein . . . 5
1.6.7 Data type for expressional targets . . . 5
1.6.8 Using custom resources . . . 5

2 Supplementary Figures **5**

3 Data S1 **11**
3.1 Robustness . . . 11
3.2 Reproducibility . . . 11
3.3 Perturbation studies with short or long readout times . . . 11
3.4 Evaluation of effect sizes in all analyses . . . 12
3.5 Previously published methods for pathway analysis of proteomic datasets . . . 13
3.5.1 Temporal Pathway Synthesizer (TPS) . . . 13
3.5.2 PARADIGM . . . 16
3.5.3 pCHIPS . . . 16
3.5.4 SigNetTrainer . . . 17
3.5.5 PHONEMeS . . . 17
3.5.6 Method from Chasman *et al.* . . . 17
3.5.7 PhosphoPath and PTMapper . . . 17
3.5.8 PCST . . . 17
3.5.9 PHOTON . . . 17
3.6 Library of graphical patterns used for causality detection . . . 18
3.7 Extending graphical patterns . . . 31

1 Supplemental Experimental Procedures

1.1 Significance for proteomic data change and correlation

For comparison-based analyses we used a two-tailed t-test for calculating the significance of the difference of the means of the two groups in the comparison, requiring the presence of at least 3 non-missing values from all compared groups. For correlation-based analyses we used the Pearson correlation coefficient and its associated significance, requiring at least 5 samples in the calculation. Both tests assume a null model where molecular readouts change independently. The EGF stimulation dataset provides pre-calculated p-values for all of the pairs of time points [1], which we directly used in the analysis. For the analysis of the cell line dataset from Hill *et al.*, we used the "complete" version of their dataset and did not do any sample or antibody filtering. We used a paired two tailed t-test to compare readouts at multiple time points collectively. In all calculations, we used the Benjamini-Hochberg (BH) method for controlling false discovery rate (FDR), whenever applicable. Prior to the application of the BH method, the software detects data rows that have potential to become a part of a causal hypothesis, without considering their significance or direction, and applies the BH method to only these rows. This step filters out data rows whose significance or direction will never be evaluated during any causal reasoning, and prevents over-correction. We used 0.1 as a default FDR threshold unless indicated otherwise.

1.2 Causality

Both pathway inference and pathway extraction are closely related to formal notions of causality inference, specifically to the Suppes' and Pearl's probabilistic formulations. A probabilistic causal relationship between two events, say from event A to event B, indicates the probability that B depends on the status of A, as described by Patrick Suppes [2]. While using this notion of causality can generate predictive models, it does not tell if A may cause B. For instance, there can be an event X that is causing both A and B, and this will still satisfy Suppes' formulation. To make the model predictive under an intervention scenario, Judea Pearl provided a reformulation: perturbing the status of A will change the probability of B [3]. We follow Pearl's notion and detect mechanism-based evidence for activity change of one protein may affect the abundance of a specific peptide from another protein in pathway databases, as described in the next section and in Supplementary Information.

1.3 Derivation of prior relations from detailed mechanistic pathways

Using the BioPAX-pattern framework, and by studying the structure of the BioPAX models from different resources, we manually defined 12 BioPAX patterns to capture potentially causal binary relations that involve phosphorylation and expression of proteins. We provide the details of these patterns in Supplementary Information, along with examples for what they can detect. The source code of the software that we used for extracting causal priors can be found at https://github.com/PathwayAndDataAnalysis/causal-priors-extractor. We applied the version 1.0.0 of this code on Pathway Commons v9 to generate a part of the causal priors that we used in this study.

1.4 Algorithm for selection of explanatory subset of prior relations

Using the extracted causal priors, CausalPath determines if there is sufficient proteomic data that indicates differential activity of that prior. The pseudocodes below implement the logical equations that check conditions of causality.

Comparison-based detection (algorithm that tests Eq. 1):

```
For each prior relation pr
    s ← pr.source
    t ← pr.target
    if pr is phosphorylation or dephosphorylation
        for each phosphopeptide measurement mpt on matching sites of t
            for each total protein measurement mts on s
                if mts.sign * pr.sign * mpt.sign = 1
                    add pr to results
            for each phosphopeptide measurement mps on s
                if mps.effect * mps.sign * pr.sign * mpt.sign = 1
                    add pr to results
    else if pr is upregulation or downregulation of expression
        for each total protein measurement mtt on t
            for each total protein measurement mts on s
                if mts.sign * pr.sign * mtt.sign = 1
                    add pr to results
            for each phosphopeptide measurement mps on s
                if mps.effect * mps.sign * pr.sign * mtt.sign = 1
                    add pr to results
```

Correlation-based detection (algorithm that tests Eq. 2):

```
For each prior relation pr
    s ← pr.source
    t ← pr.target
    if pr is phosphorylation or dephosphorylation
        for each correlated s tot-prot and site-matching t phosphoprot
            if corr.sign * pr.sign = 1
                add pr to results
        for each correlated s phosphoprot (mps) and site-matched t phosphoprot
            if mps.effect * corr.sign * pr.sign = 1
                add pr to results
    else if pr is upregulation or downregulation of expression
        for each correlated s tot-prot and t tot-prot (mtt)
            if corr.sign * pr.sign = 1
                add pr to results
        for each correlated s phosphoprot (mps) and t tot-prot (mtt)
            if mps.effect * corr.sign * pr.sign = 1
                add pr to results
```

These algorithms use *.sign* and *.effect* properties of variables that takes values -1, 0 and 1, corresponding to *false*, *unknown* and *true*, respectively. Multiplication of these integer values and checking the result value is an alternative formulation to the original logical equations where ternary XOR ($\oplus$) operator is used. When the RNAseq data is used at the targets of expressional control relations, the total protein measurements (*mtt* in the pseudocode) are replaced with the RNAseq measurements of the target (*mrt*).

1.5 Description of causal graph notation

We developed a new graph notation to represent resulting causal explanations as a logical network, where nodes denote proteins and edges denote causal relations (Fig. 3c). Node background is used for color-coding total protein change, while site-specific changes are shown with small circles on the nodes whose border colors indicate whether the site is activating/inhibiting. If additional omic data such as RNA expression, DNA copy number, or mutation status are available, we include them for integrated visualization, using small circles displaying specialized letters. Binary causal relations are represented with edges—green representing

positive, red representing negative, phosphorylations with solid edges and transcriptional regulations with dashed edges. When significance calculation results are available, they are represented on the protein borders, using a bold border when downstream of a protein is significantly large, green border when downstream indicates the protein is activated, and red border when downstream indicates the protein is inactivated. If both the activation-indicating and inhibition-indicating downstream relations are significantly large, then a dark yellow color is used instead. We use topology grouping while rendering result networks, which means we group the proteins with the same network topology under compound nodes on the network for complexity management. To further clarify the graph notation, we provide examples in Supplementary Figure S4.

1.6 CausalPath parameters

CausalPath is designed to explore omic datasets of different sizes, types, and accuracy. Following are some important parameters to consider when using the method.

1.6.1 Site matching proximity threshold

Protein phosphorylation sites in the literature have to exactly match the detected site in the phosphoproteomic dataset to use in causal reasoning by default. Some users may find this too strict since there can be slight shifts in the literature, or some nearby sites of proteins are likely to be phosphorylated by the same kinase. This parameter makes the analysis allow a determined inaccuracy in site mapping to explore such cases. Increasing this parameter will increase the result network size by allowing proximate site matching (Suppl. Fig. S5, blue bars). This parameter can be used to increase to coverage of the results, however, the new relations in the results are likely to have more false positives than other relations. In this manuscript, we used strict site-matching for all the CausalPath analyses unless indicated otherwise.

1.6.2 Site effect proximity threshold

The effect of the phosphorylation sites on the protein activity, as in activating or inactivating, is curated by pathway databases, mostly by PhosphoSitePlus. We also did some small-scale curation for EGF stimulation analysis, and RPPA analyses. CausalPath requires exact matching of these curated site effects with the sites in the data by default, however, this can be too strict because nearby sites generally tend to have similar effects. This parameter lets the analysis use a determined inaccuracy while looking up site effects. Increasing this parameter will increase the result network size by reducing the portion of phosphorylation sites with unknown effect (Suppl. Fig. S5, red bars). Similar to the previous parameter, this parameter increases coverage at the cost of increase in false positives. In this manuscript, we always used accurate matching for site effects.

1.6.3 Gene focus

This parameter lets the analysis use a subset of the literature relations, focusing on the neighborhood of certain proteins indicated by their gene symbols, hence reducing the number of tested hypotheses. We used this parameter during the analysis of ovarian cancer subtypes as described in the relevant section. Gene focus may be useful in two ways: (i) removes irrelevant parts of the prior relations, providing a means of complexity management, (ii) may increase statistical power for differential abundance detection by decreasing the total number of tested peptides.

1.6.4 Generation of a data-centric causal network

CausalPath result networks are gene-centric, meaning that genes are represented with nodes, and all other measurements related to a gene are mapped on the gene's node. When a data row can map to multiple genes, however, this creates a redundancy, as we have in the RPPA analysis results. For example, the AKT phosphoantibody can recognize all three AKTs, so we duplicated the same data on AKT1, AKT2, and AKT3. A similar problem exists for mass spectroscopy when a phosphopeptide can be resolved to multiple homologous proteins, if their sequences are identical around the phosphorylation site, AKTs again being an example. As an alternative to this view, CausalPath can generate data-centric views where nodes represent

data rows unresolved to particular proteins. But this view does not support mapping other available -omics data onto the network, and also the relations are duplicated when more than one data of the same gene can be explained by the same relation.

1.6.5 Protein activity

CausalPath allows users to insert their own hypotheses as to whether a protein is activated or inhibited in the case of a comparison-based analysis. The input has to be a gene symbol associated with a Boolean parameter indicating the hypothesized direction of activity change. We used this option for EGF stimulation analysis to indicate that we expect EGF to be activated because the data only measures phosphoprotein abundances, hence there is no measurable change on EGF itself to include in the analysis otherwise.

1.6.6 Adjusting phosphopeptide measurements with total protein

When both phosphoprotein and total protein measurements are available in a study, an optional adjustment can be done to the phosphopeptide values to reflect their relative abundance to the total protein, before applying CausalPath. Analyses in this manuscript do not include this type of adjustment. Each protein feature is independently used in causality checks.

1.6.7 Data type for expressional targets

CausalPath restricts its logical reasoning within proteomic data by default, however, users can opt to use mRNA data for the targets of expressional relations. We used this parameter during the correlation-based analysis of ovarian and breast cancers as described in the results section. It is also possible to use mRNA and protein data together by using this parameter multiple times.

1.6.8 Using custom resources

CausalPath resources are embedded in its code base by default, and they are subject to change with new versions of the software. To establish reproducibility and customizability, CausalPath allows users to override the following resources: (i) list of priors relations using the "custom-prior-relations-file" parameter, (ii) list of known site effects using the "custom-site-effects-file" parameter, (iii) list of recognized HGNC symbols using the "hgnc-file" parameter. For this manuscript, we provide these resources in a zip archive, to ensure reproducibility and to provide example for resource customization.

2 Supplementary Figures

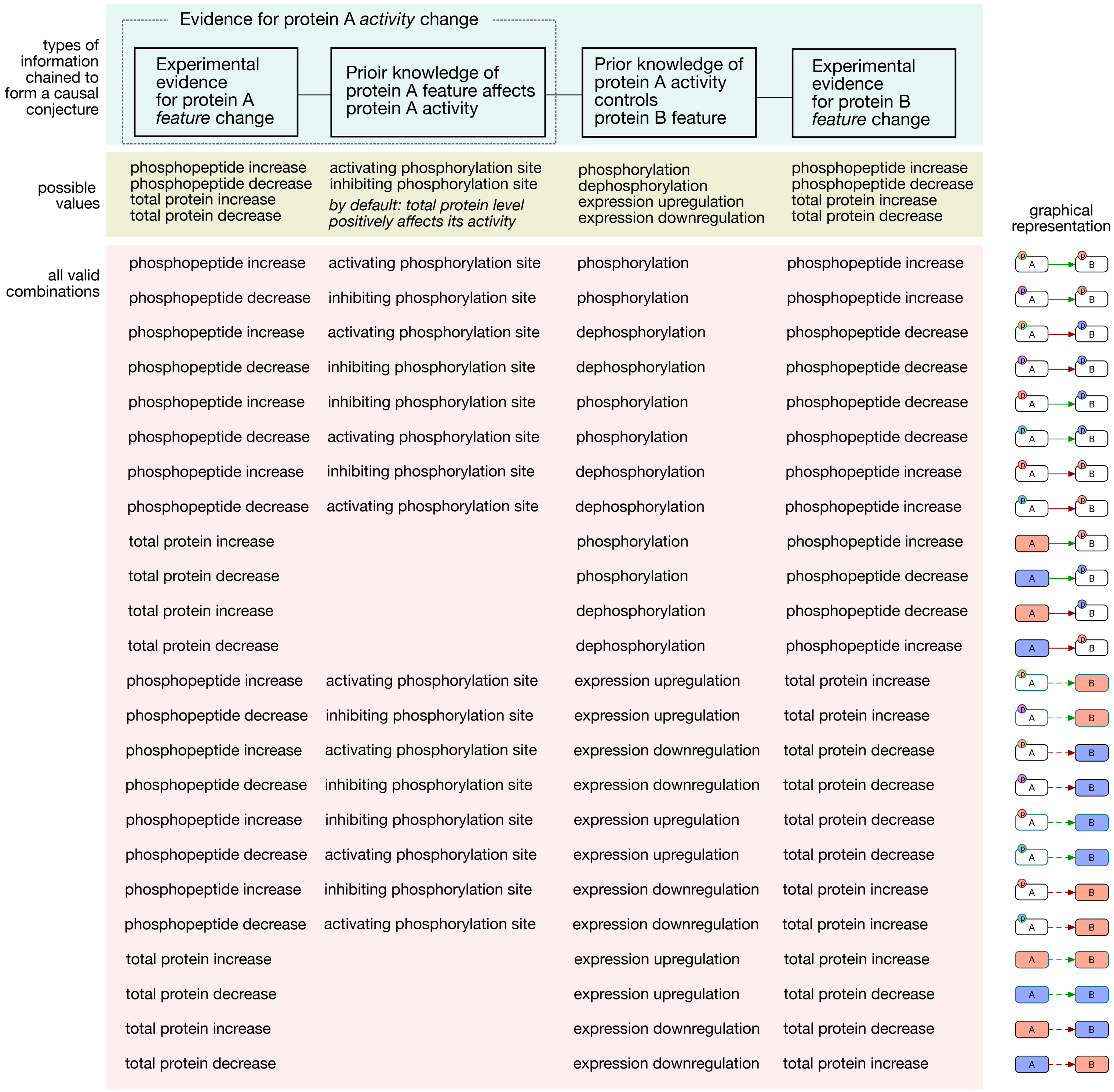

Figure S1: Causal conjecture generation for comparison-based analysis (Step 2 of CausalPath workflow). CausalPath integrates 4 types of information such that the prior information forms a causal bridge between a pair of observed proteomic changes. Each type of information has a fixed number of possible values. Valid combinations of these values are detected using Eq. 1, also iterated here.

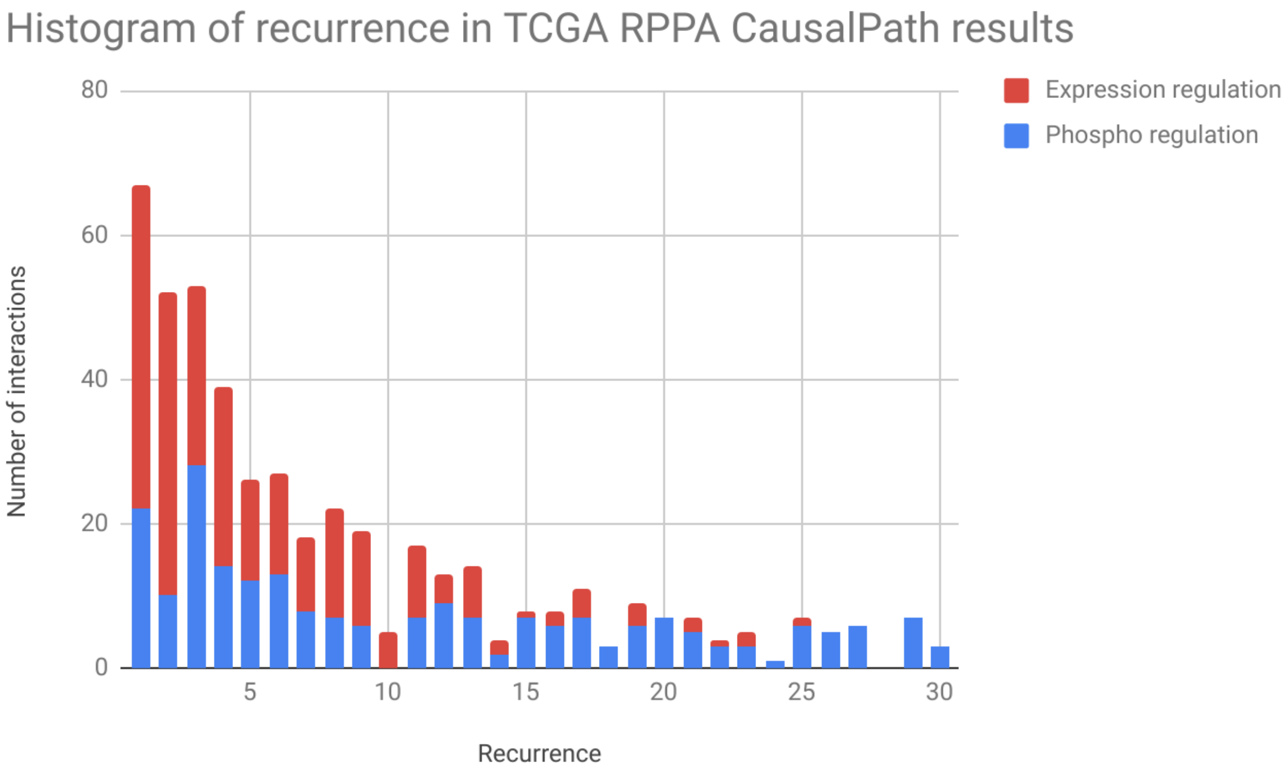

Figure S2: Recurrence histogram for the relations in the results from CausalPath analysis of TCGA RPPA datasets. The histogram has a significant long tail showing a subset of the results are highly recurrent. While the total number of expression regulations and phospho regulations are similar, the high-recurrence tail is dominated by phospho regulations.

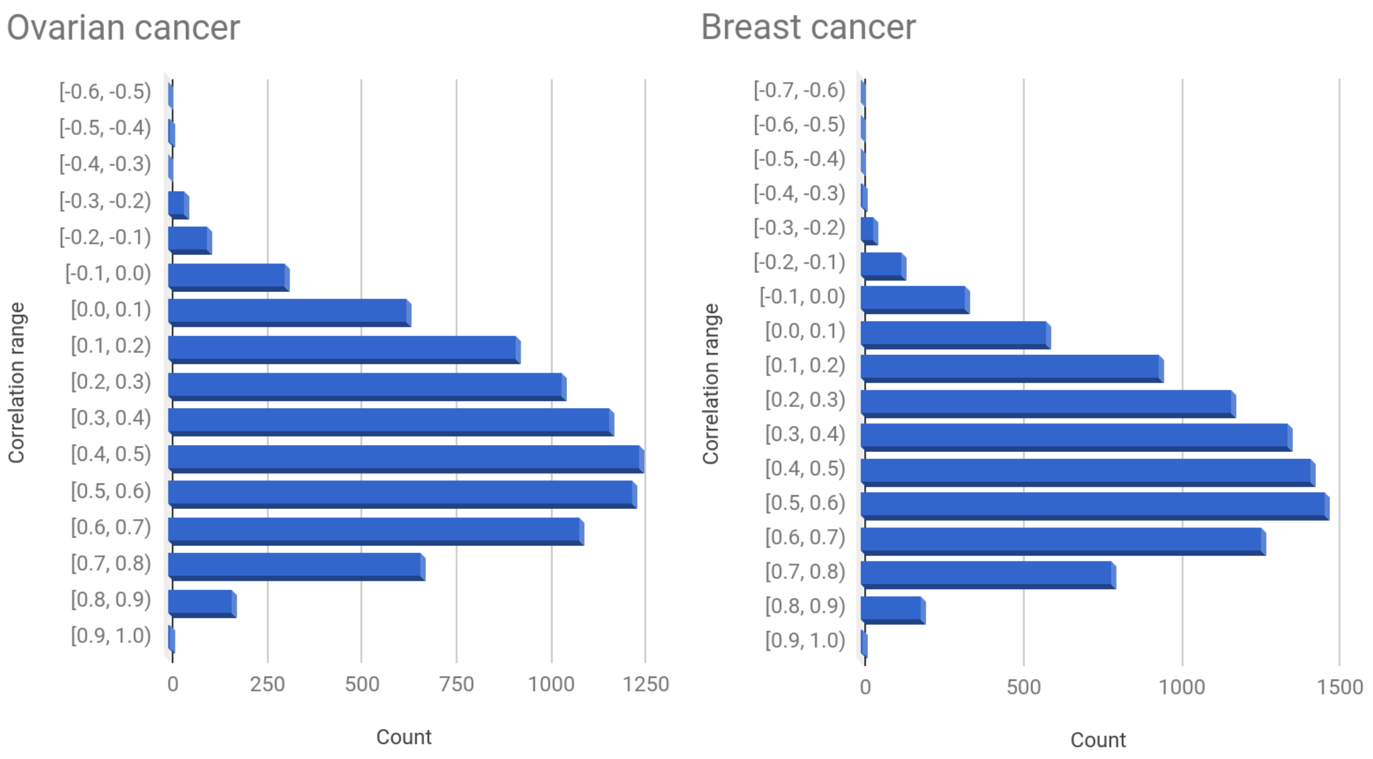

Figure S3: Histograms for the distribution of correlations between mRNA and protein expressions of genes in ovarian and breast cancer datasets. Both datasets have similar distributions, peaking around 0.5 Pearson correlation.

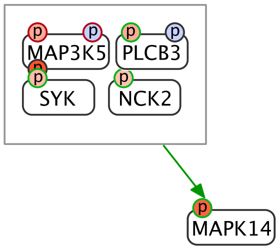

4 proteins are grouped together as possible cause for MAPK14 phosphorylation. This grouping does not imply that they form a complex, but they are grouped only based on their topology in this graph, so that the phosphorylation relation is not drawn 4 times. Each of the 4 proteins can have a different mechanism for phosphorylating MAPK14. MAP3K5 has 3 site-specific measurements on it, two of them are inactivating (red border), and one has unknown effect (black border). Among inactivating, one goes up and one goes down. Here the causal hypothesis is constructed with the site that goes down. The other site change is in conflict with this hypothesis but shown anyway for completeness. Even though the arrow seems to be pointing at "p", this is only a coincidence as CausalPath graphs do not show edge-to-feature mapping, but they may be logically inferred.

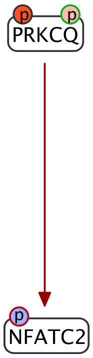

NFATC2 is activated by dephosporylation of its inactivating site. This dephosphorylation can be due to PRKCQ phosphorylation and activation. This relation is unintuitive because PRKCQ is a kinase and not a phosphatase. The causal link from PRKCQ to NFATC2 is a relatively complex sequence of events (right), involves FLNA/PRKCQ complex increasing the intracellular calcium levels, and the calcium inhibiting the inhibitory phosphorylations on NFATC2. This example demonstrates that it is important to not to assume simple phosphorylation/ dephosphorylation when reading CausalPath graphs as they can be more complex indirect mechanisms.

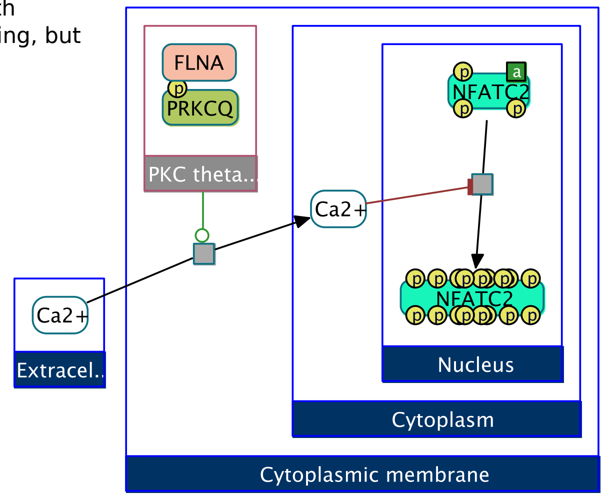

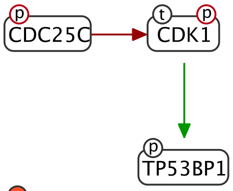

A correlation-based graph of 3 proteins. CDK1 has 2 measured features: total protein (t) and phosphorptein (p). The graph does not show which relation is related to which feature, but in this case, it is inferrable. The target of the dephosphorylation (red edge) has to be the "p" because we do not expect a total protein change with dephosphorylation. As a result, this graph indicates presence of a positive correlation between CDC25C-p and CDK1-p. The source of the phosphorylation (green) relation can either be "t" or "p" as both can be a proxy for the activity of CDK1.

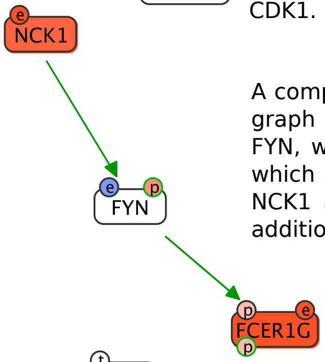

A comparison-based graph where RNA expression changes are also displayed on the graph (e). The graph shows that NCK1 total protein increase may have caused phosphorylation and activation of FYN, which in turn may have caused phosphorylation of FCER1G. The graph does not distinguish which site of FCER1G is phosphorylated by FYN, and can be both. We see that RNA expressions of NCK1 and FCER1G are increased, and RNA expression of FYN is decreased, but these are only additional information and were not used in causal reasoning.

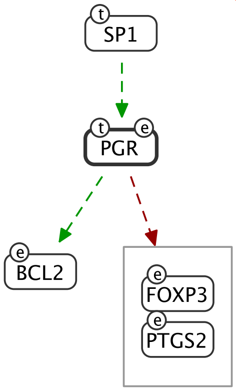

A subgraph from results of a correlation-based analysis where RNA expression data is used at the targets of expression regulations for causal reasoning. SP1 total protein is positively correlated with PGR RNA expression. PGR total protein is positively correlated with RNA expression of its positive target BCL2, and it is negatively correlated with RNA expressions of its negative targets FOXP3 and PTGS2. The sign of these correaltions are not shown on the graph, but we infer them from the nature of their relationships. PGR has a bold border indicating its downstream is significantly large on the network. This can be unintuitive since 3 is not a large number. There can be two reasons for a significance with a few downstream targets: (i) the number of targets in the base network (causal priors) may be a low number, which makes 3 significantly large, (ii) there may not be many RNA expressions correlated with PGR total protein overall, which again makes 3 significantly large.

Figure S4: A selection of examples from CausalPath results (left) and their textual explanation (right).

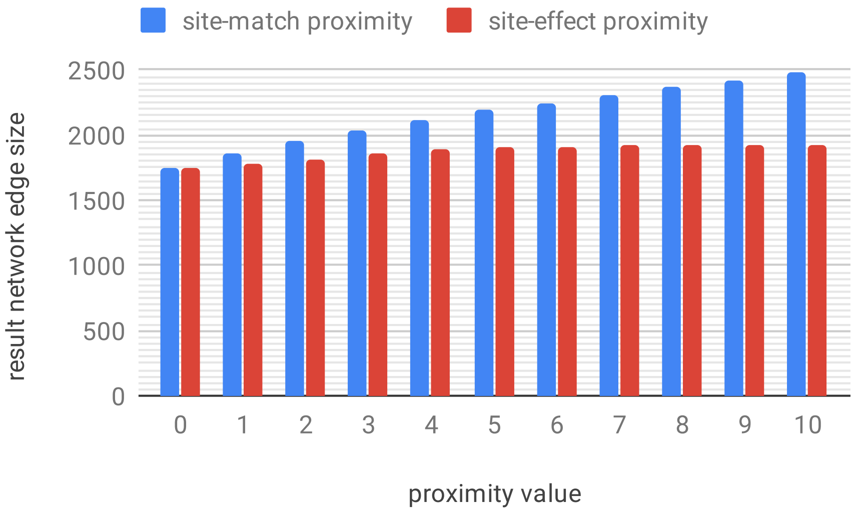

Figure S5: The effect of the parameters “site-match-proximity-threhsold” and “site-effect-proximity-threshold” on the result size of the correlation-based analysis of CPTAC breast cancer dataset using phospho-relations. Increasing the first parameter makes an higher impact on the result size than increasing the second parameter.

3 Data S1

3.1 Robustness

We tested the robustness of CausalPath against noise on the CPTAC breast cancer dataset, on a correlation-based analysis with phosphorylation relations using 0.1 FDR cutoff. We iteratively and cumulatively introduced noise into the dataset by adding random numbers to the proteomic data, drawn from a normal distribution, and generated the causal network. At each step, we recorded the amount of the overlap with the original relations (Fig. S6a). Ideally, as the noise level increases, we would like the method to retain the original results and not allow the noise to generate non-overlapping new relations. Our tests indicate that CausalPath is sensitive to noise, which means overlapping relations rapidly start decreasing in number, however, the method is safe against noise, which means we do not get many non-overlapping relations due to it. This means that the data quality highly affects the size of the results, but it is not risky to try CausalPath on high-noise datasets.

We next assessed the importance of the quality of causal priors on the quality of the results using the same dataset with data label randomizations. Randomization of data labels means random assignment of data rows to the prior network. The chance of a network relation to get accidentally selected into the results after randomization is roughly equal to a random false relation to accidentally pair with compatible data and to get selected into the results. After data label randomization, the correlation-based analysis of CPTAC breast cancer dataset on phosphorylation controls produce an average of 1,127 relations (with a standard deviation of 88) out of 27,196 prior relations. This means a random incorrect causal prior has about 4% chance to generate noise in the results. This is low enough for the method to tolerate occasional inaccurate priors, but the method definitely depends on high-quality of the causal priors to generate useful results. Based on this observation, we used only manually-curated high-quality prior data in our analyses.

3.2 Reproducibility

We tested the reproducibility of CausalPath on the CPTAC breast cancer dataset by using random subsets of the samples, on a correlation-based analysis with phosphorylation relations using 0.1 FDR cutoff. In a total of 100 trials, we used a random half of the samples at each step and checked how frequently each causal relation is reproduced in the results and checked their overlap with the original results where all of the samples were used (Fig. S6b,c). The results indicate that half of the overlapping relations are reproduced in at least 61% of the trials consistently (yellow mark in Fig. S6c), while 11% are reproduced in all of the trials (last bar in Fig. S6b). The significant amount of non-overlapping relations (red) with low reproduction counts in the tests are due to the accumulation of false positives from all 100 trials.

3.3 Perturbation studies with short or long readout times

For the analysis of a perturbation study with time resolution, CausalPath may not be equally successful to explain each time point. Early time points (minutes to hours) are shaped by short range, direct effects and late time points (hours to days) mostly reflect long range, indirect effects. It can be argued that CausalPath is more suitable for the analysis of short range effects due to the nature of pathway databases and due to the way CausalPath uses the pathway knowledge. To investigate this, we repeated the precision estimation study in the section "Precision of CausalPath results on cell lines stimulated with ligands", but this time using only earlier half of the time points or later half of the time points in the study. We were able to test a fewer number of relations in both cases because halving the dataset reduced the statistical power. Instead of 32, we were able to test 12 and 2 relations in the earlier and later time points, respectively (Fig. S7). "Early" test results were similar to the original results in shape, even though we have much fewer measurements to characterize it. In the "late" case, CausalPath generated fewer explanations for the proteomic changes upon ligand stimulation and the portion of them that are testable with the existing drug inhibitions only has 2 relations—both yield insignificant changes with drug inhibition. While this may be due to a worse precision, we do not have enough data to decide. It is possibly true that CausalPath can explain short time courses better than long time courses, however, in this case, it is better to use them together and have a higher statistical power for the paired t-test.

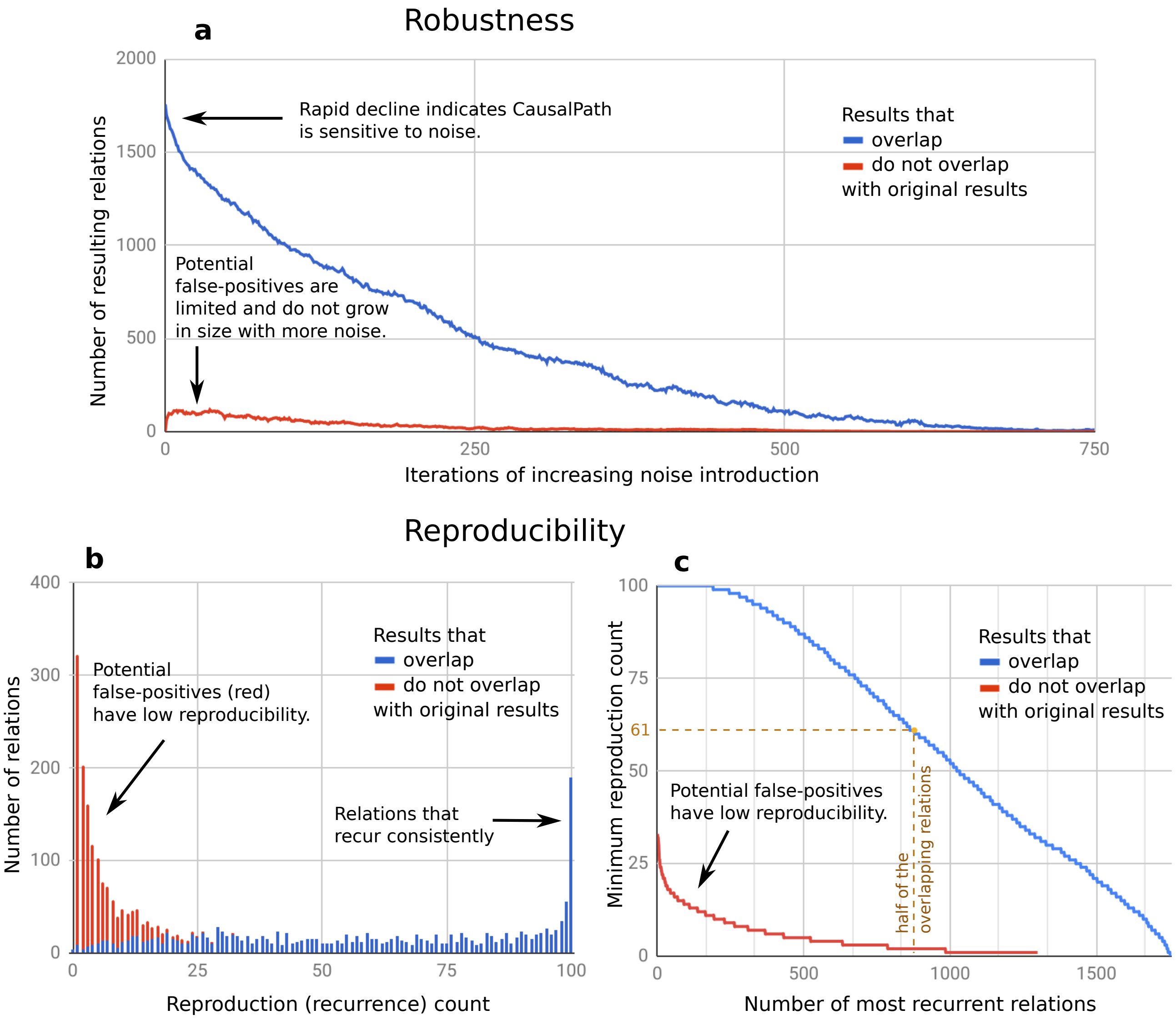

Figure S6: Robustness and reproducibility of CausalPath results on CPTAC breast cancer dataset with phospho relations. (a) Robustness is tested by iteratively introducing noise into proteomic data and then testing overlap of the new CausalPath results with the original results. (b, c) Reproducibility is tested by running CausalPath on random halves of samples. The new results are compared to the 1,756 relations in the original results where all of the samples were used. Non-overlapping results are likely dominated by false positives. The two reproducibility charts describe the same experiment from different viewpoints. Left chart (b) is a histogram of reproduction counts of result relations, while the right chart (c) is cumulative, showing the minimum reproduction ratios of the most reproducible result relations.

3.4 Evaluation of effect sizes in all analyses

When large datasets are analyzed for correlated or changed features, and a p-value threshold from a statistical test is used for identification of significant changes, it is possible that some features with very small effect sizes may pass the p-value threshold. When this happens, the conclusions derived from the results become questionable because of the small size of the effects. We tested if the analyzes in this manuscript are affected from small effect sizes and we have not detected any concerning cases. There are 391 individual CausalPath analyzes that contributed to this manuscript, corresponding to the same amount of analysis folders in the Suppl. Archive. Figure S8a provides an example distribution of effect sizes in a correlation-based study.

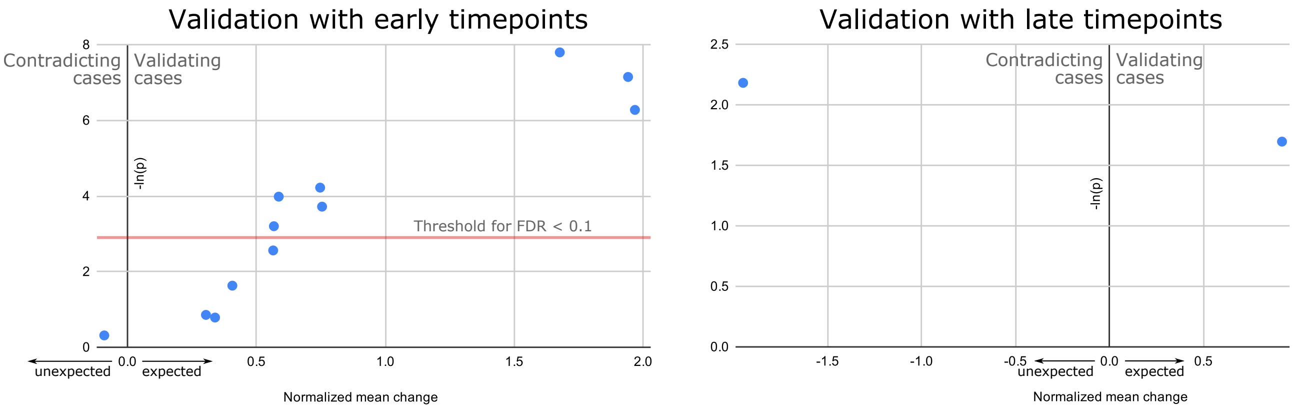

Figure S7: Results of limiting the precision estimation study to early or late time points.

Blue bars are the distribution of all pairwise Pearson correlations in the CPTAC breast cancer proteomic dataset (total protein and phoshpo combined), and the red bars indicate the count of selected features by CausalPath to use in causal hypotheses for explanation of phosphoproteomic changes, corresponding to the specific correlation range (blue and red bars are drawn in different scales, please notice two vertical axes). Here, we observe that there is no feature selection when the correlation is close to zero. In this example, the smallest absolute value of the selected correlations is 0.25. We checked how this smallest value changes in all the correlation-based analyses (Fig. S8b), and found that the average of the smallest values is 0.35, and the smallest of the smallest values is 0.12.

We did a similar check on correlation-based analyses. As an example, Figure S8c represents the comparison of luminal breast cancer to the basal subtype, again on CPTAC breast cancer proteomic dataset. Blue bars show the distribution of all changes, normalized with the standard deviation of that feature. Red bars are the distribution of the features that CausalPath selected for using in causal hypotheses (again, shown in a different scale). We observe that there are no red bars very close to zero change, indicating very small effect sizes are not used in the results. The smallest selected effect size in this analysis is 0.45 standard deviation. We checked how this smallest effect size changes in all comparison-based analyses, and found that the average of the smallest effect size is 1.15 and the smallest of the smallest effect sizes is 0.32 (Fig. S8d). (Note: Some of the comparison-based analyses use a paired t-test. In such cases, the normalization factor is not the standard deviation of the feature, but it is the standard deviation of all the paired difference values for that feature.)

3.5 Previously published methods for pathway analysis of proteomic datasets

There is no method comparable to CausalPath for its ability to identify causal relations from pathway databases that can explain given proteomic datasets. There are, however, methods developed for other forms of pathway analysis for proteomics. These methods generally use prior information in the form of networks stripped from mechanistic details, and aim to build a network structure that most fits to the profiling data at hand using an optimization method. During the development of CausalPath, instead of a network optimization, we intentionally focused on better usage of prior information by processing mechanistic details in pathway databases and using them in logical reasoning for causality. CausalPath, in that sense, is not competing with these alternative methods, but it is complementary to them. CausalPath can easily be paired with any other network optimization method for further complexity management and for using strong priors in network optimization. Below is a short survey of these other pathway analysis methods.

3.5.1 Temporal Pathway Synthesizer (TPS)

TPS uses PPI and kinase-substrate networks in the process of inferring signaling relations from temporal post-perturbation proteomic data [1]. The method first runs a prize-collecting Steiner tree (PCST) algorithm between the perturbed agent and the modified proteins to reduce the PPI network, then it uses this reduced

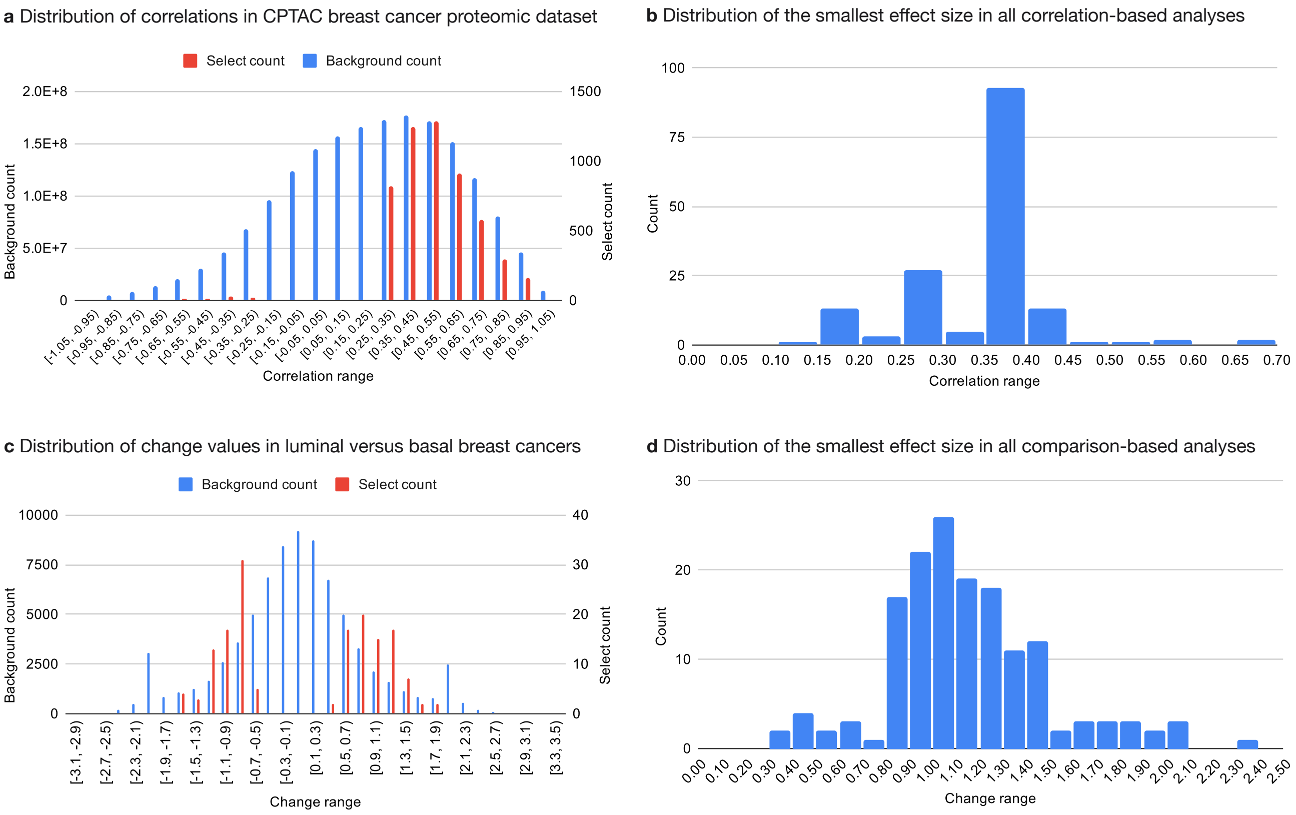

Figure S8: Illustration of the effects sizes used in causal hypothesis generation. Upper charts are related to correlation-based analyses and lower charts are related to comparison-based analyses. Charts on the left are example cases from CPTAC breast cancer proteomic dataset. Charts on the right are the overall distributions of the smallest effect size in each analysis. The average background correlation on (a) is 0.23 and the average background change value on (c) is -0.03.

network and the substrate-kinase network as constraints in its search for a model that do not violate temporal constraints (upstream changes are not happening after downstream changes) observed in the data. TPS does not consider if a measured phosphorylation site is activating or inhibiting of its downstream activity, and it does not check if the kinase phosphorylates from the matching location of the substrate observed in the data. Also, TPS is limited to perturbation studies with temporal proteomic profiling.

The EGF stimulation study that we analyze in the results section was originally produced by the authors of the TPS method to demonstrate its capabilities. A comparison of our results with theirs indicates large differences in resulting models. TPS finds 413 directed relations that fit in the constraints of the system. CausalPath, on the other hand, identifies 50 relations collectively for all time frames (all unique relations in the Suppl. Animation 2). Only 3 of these relations are overlapping, one of them being EGF $\rightarrow$ EGFR. There are many reasons for this huge difference in results. TPS is more focused on evaluating the temporal constraints and less focused on the strength of the priors. For example, when two proteins are known to be interacting and this interaction is selected by PCST, TPS algorithm puts a directed edge between them according to the timing of their phosphorylation changes. From CausalPath perspective, this is a very loose inference because a protein interaction is very weak evidence for a causal effect. CausalPath looks for prior knowledge of a phosphorylation relation and the knowledge of site-effect for the regulator. This results in fewer relations but stronger hypotheses due to their evidence of existence in at least one experimental setup. From TPS perspective, CausalPath misses the information embedded in the temporal order of the phosphorylations. During the development of CausalPath, we considered evaluating the order of phosphorylations when this data is available but decided against it due to several reasons. The most important reason lies in the nature of statistical detection methods that we use. When we detect a phosphorylation change, comparing cases against controls using a statistical method, we often choose a stringent significance threshold to keep the false positives under a certain amount. But a stringent threshold practically means a high level of false negatives—the cases that we miss to detect. Accordingly, we can trust the positives in the results more than the negatives. This is generally acceptable when we base our interpretations only on the positive results, just like we do in CausalPath, finding the explanatory causal relations between positive results. However, when we use negative results in the interpretation, such as *lack* of phosphorylation change, the high false negative rate would cause a high rate of misinterpretations. For instance, to infer a directed relation from A to B, TPS requires B is not phosphorylated before A, however, due to the high rate of false negatives, B may be actually phosphorylated before A but not showing up in the results. To properly check for this constraint, one should design an experimental setup and a companion statistical test that evaluate the timing of peptide changes, and detect one peptide increased/decreased *significantly sooner/later* than the other. The second reason for our not focusing on temporal order is that we consider that an effect can have multiple causes. Cause 1 may generate the Effect at an early time point, then Cause 2 can appear and start supporting the Effect in a later time point. We would not like to miss such multi-cause effects, especially when they are already documented in the literature, and considering Cause 1 may be invisible to the analysis.

We tried to estimate the precision of TPS using the same procedure that we applied to CausalPath in the main manuscript, section "Precision of CausalPath results on cell lines stimulated with ligands". This evaluation is not straightforward because TPS does not claim to generate falsifiable hypotheses as CausalPath does, but only generates a relevant network, where the relevancy is not biologically well-defined. However, the presence of directed edges in the output network, and the requirement of temporal evidence implies causality between the mapped proteomic measurements. We assessed the precision of this implication using the RPPA experiment results from [4] where cell lines are treated with protein ligands and inhibitor drugs. We generated a result network for each cell line - ligand stimulation experiment, then we used the drug-treated samples to validate directed relations in the results. More specifically, if the source protein of a directed edge in the results has a prediction of "activation" on a time point, and that protein is targeted by a drug in the experiments, we expected the measured feature of the downstream protein of the relation will change in the reverse direction when the drug is applied, on the same or later time points. Using the measurements on 4 cell lines with 6 ligands (skipping PBS case because TPS requires the target of perturbation as input) and 5 drugs allowed us to test a total of 54 directed edges in TPS results. Out of 54, we observed a change towards the expected direction in 34 cases, and towards the unexpected direction in 20 cases (Fig. S9). 6 of the changes pass a 0.1 FDR threshold, where 5 of them are in the expected direction. While we observe a higher number of changes in the expected direction, this imbalance is not significant (P

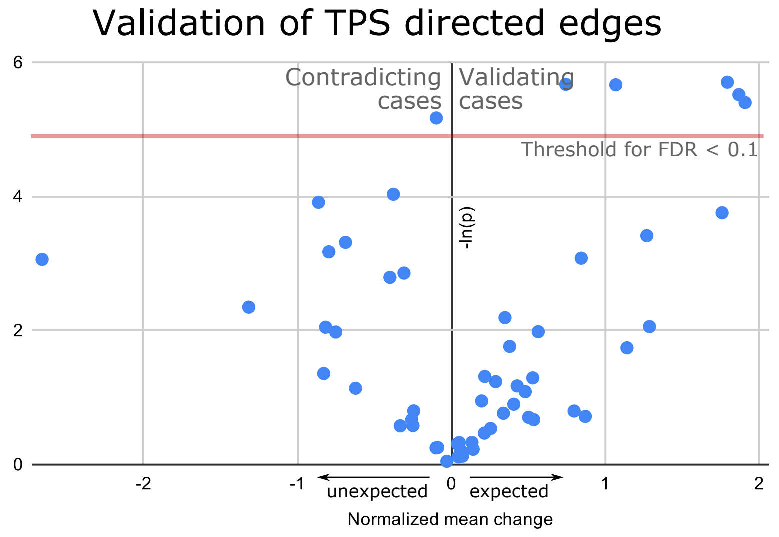

Figure S9: Evaluation of the precision of TPS directed edges on the RPPA experiment of ligand-stimulation and drug-treatment of breast cancer cell lines. Positive direction on the x-axis is the expected direction of change of downstream proteins upon drug treatment. Negative is the unexpected direction. Red line indicates a threshold of 0.1 FDR in the detected changes.

= 0.075, binomial test). If we use the same symmetrical-noise assumption that we used for estimating the precision of CausalPath, we assume all 20 changes on the unexpected direction are noise and expect to have the same amount on the expected direction. This predicts that 14 surplus changes on the expected direction are actual changes, which means 14 of 54 cases validate positive, amounting to a precision of 0.26. The less-specific use of prior knowledge by TPS is the most likely cause for this lower precision. A second factor is the reduced statistical power. The temporal angle of TPS makes it require more data for similar statistical power. Limiting a t-test to a specific time window reduces the number of samples used in the calculation. We predict if there were more replicates in the RPPA experiment, TPS precision would increase. The input and output files for this analysis can be found in the Suppl. Archive.

3.5.2 PARADIGM

PARADIGM is one of the earliest pathway analysis methods developed originally for RNA expression and copy number variations and later extended to other data types including proteomics [5]. It uses the pathway models from NCI Pathway Interaction Database (PID), converts them into a factor graph, and predicts each entity's activity level using an expectation-maximization algorithm. PARADIGM does not provide any site-specific data handling. The method assumes each mapped data type is a positive indicator of activity, and does not differentiate between activating and inhibiting phosphorylations. It also does not check if a site-specific phosphorylation has a downstream phosphorylation change at the matching site.

3.5.3 pCHIPS

This is a network propagation method for proteomic and other data, based on the TieDIE [6] algorithm, where the purpose is to link differentially active kinases (indicated by proteomic data) to the differentially active transcription factors (indicated by RNAseq measurements of targets) [7]. Proteomic changes on the kinases are propagated downstream, differential transcription factor activities are propagated upstream on the signaling network, and the overlap is identified as a possible linking path or combination of paths.

While linking kinases to transcription factors implies causality, pCHIPS does not check the conditions of causality such as if the proteomic change is indicative of activation or inhibition, or if the linking path has a positive or a negative effect on the transcription factor activity, or their compatibility for a causality hypothesis.

3.5.4 SigNetTrainer

SigNetTrainer [8] is a set of algorithms that score the fitness of molecular readouts to a given set of perturbations and a given directed and signed interaction graph, and solves several interesting problems using integer linear programming, such as finding an optimal subgraph that is most consistent with the measurements, or finding minimal set of new relations that will make the network and the measurements consistent. SigNetTrainer does not perform any phosphorylation site-specific operation and does not differentiate between activating and inhibiting phosphorylations.

3.5.5 PHONEMeS

PHONEMeS [(9)] builds models in the form of Boolean networks that best fit to a given set of phosphoproteomic perturbation data. It uses a background network derived from prior information, and searches for the best Boolean network that is predictive for the given set of perturbations. The Boolean framework assumes that every protein has two discrete states, hence PHONEMeS checks the data if evidence for such a dichotomy exists, and uses only those phosphopeptides. PHONEMeS does not check for any site matching constraint, and it does not distinguish between activating/inhibiting phosphorylations.

3.5.6 Method from Chasman *et al.*

Chasman *et al.* demonstrate their network inference method on identification of yeast adaptive pathways to NaCl stress [10]. They compile a background network with directed and undirected relations, identify a set of genes/proteins by differential expression, phosphoproteome changes and stress fitness contribution, and find optimal paths from signaling proteins to gene regulation proteins employing integer programming (IP). Their method does not perform any site-specific evaluation of phosphorylations. Also the method does not distinguish between increased and decreased phosphopeptides, but considers them as *changed*.

3.5.7 PhosphoPath and PTMapper

Both methods are implemented as a Cytoscape plugin to visualize kinase-substrate relations on the protein-protein interaction (PPI) network [11; 12]. Users can run a network enrichment analysis on the PPI network for the given proteomic and other datasets, then visualize the known kinase-substrate relations on the enriched region. Neither of the methods perform any site-specific operation or evaluation.

3.5.8 PCST

This method maps proteomic and transcriptomic data on the proteins on a weighted PPI and protein-DNA interaction network, then identifies a minimal subnetwork that connects the mapped molecules, prioritizing the most reliable connections [13]. Authors formulate this as a prize-collecting Steiner tree (PCST) problem and solve with a known algorithm.

3.5.9 PHOTON

This method maps proteomic data from a perturbation study onto the proteins on a weighted PPI network, then calculates a score for each protein based on the weighted average of the observed proteomic changes on its neighbors on the network [14]. The method generates a result network by connecting the perturbed protein and the proteins with a high score on the PPI network.

3.6 Library of graphical patterns used for causality detection

Here we provide all 12 graphical BioPAX patterns that CausalPath uses to understand phosphorylation, dephosphorylation, expression upregulation, and expression downregulation relations in Pathway Commons. Each figure shows the pattern structure, a sample BioPAX data as rendered in SBGN by ChiBE, and the identified relations from the sample BioPAX using the pattern. Understanding the pattern structure requires the basic knowledge of BioPAX data structure. These patterns are implemented using the BioPAX-pattern framework, which is a Java library.

The patterns are composed of labeled BioPAX objects and certain relations between them. A common component in all patterns is the handling of generic molecules and complexes, which can be arbitrarily nested. The relation "self or more generic" allows to link the molecule towards parent generics and encapsulating complexes, the relation "self or less generic" allows link toward specific proteins and complex members.

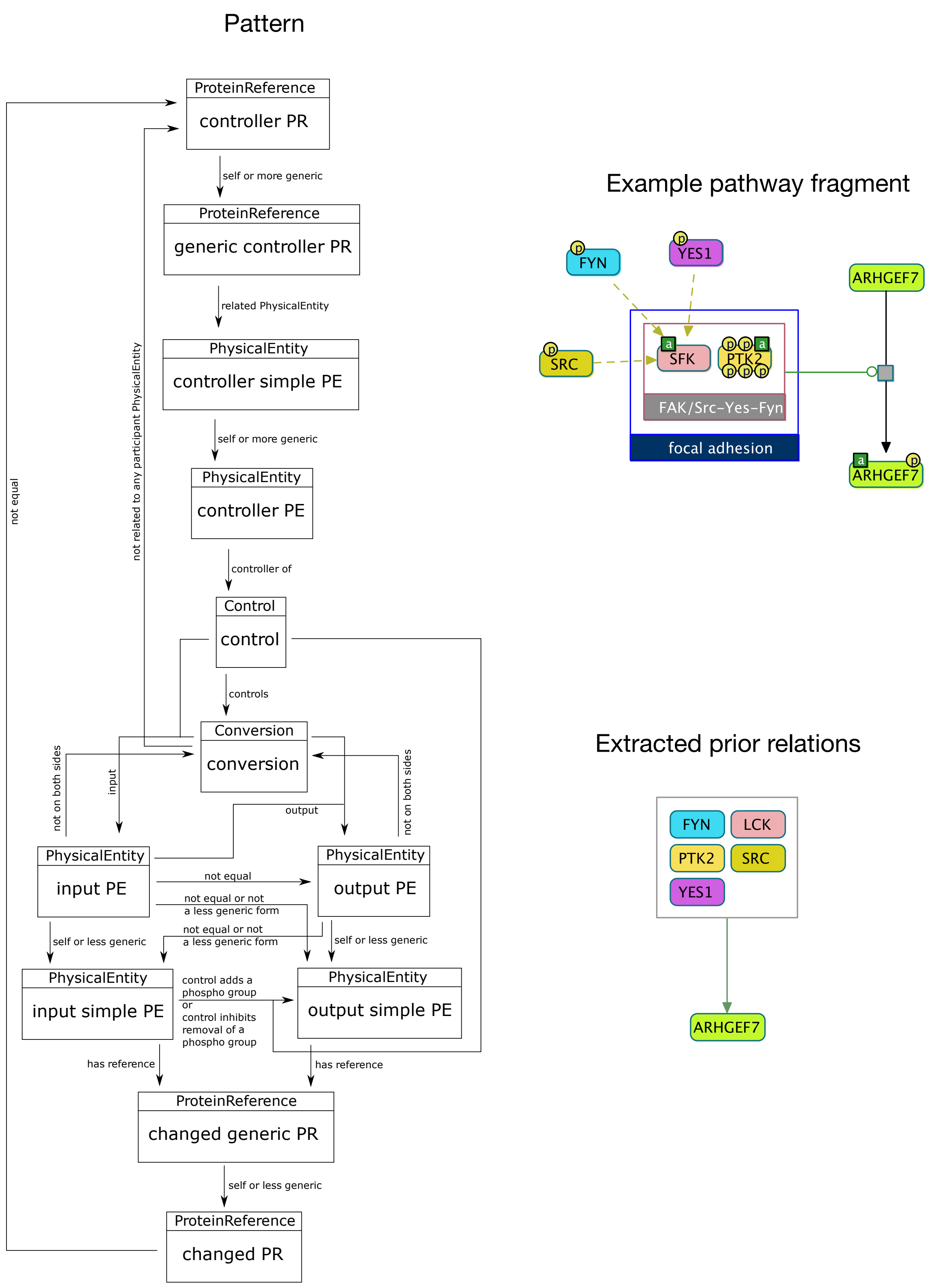

Figure S10: Pattern 1 for phosphorylation. This is the most frequently used pattern to describe phosphorylations where the regulator proteins activate a Conversion that adds phosphorylation to a protein, or inhibit a Conversion that removes phosphorylation from the downstream protein.

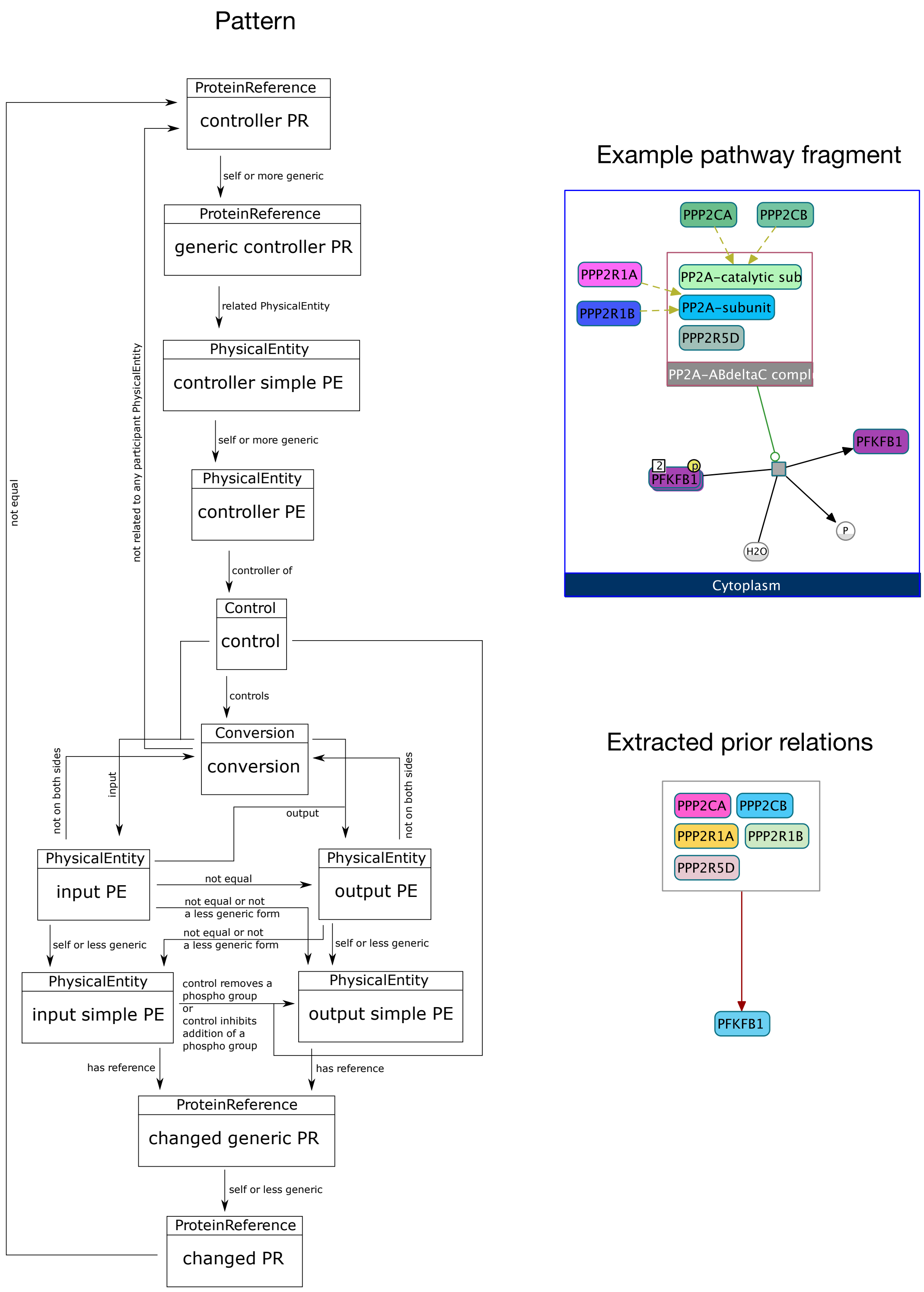

Figure S11: Pattern 1 for dephosphorylation. This is the most frequently used pattern to describe dephosphorylations where the regulator proteins activate a Conversion that removes phosphorylation from a protein, or inhibit a Conversion that adds phosphorylation to the downstream protein.

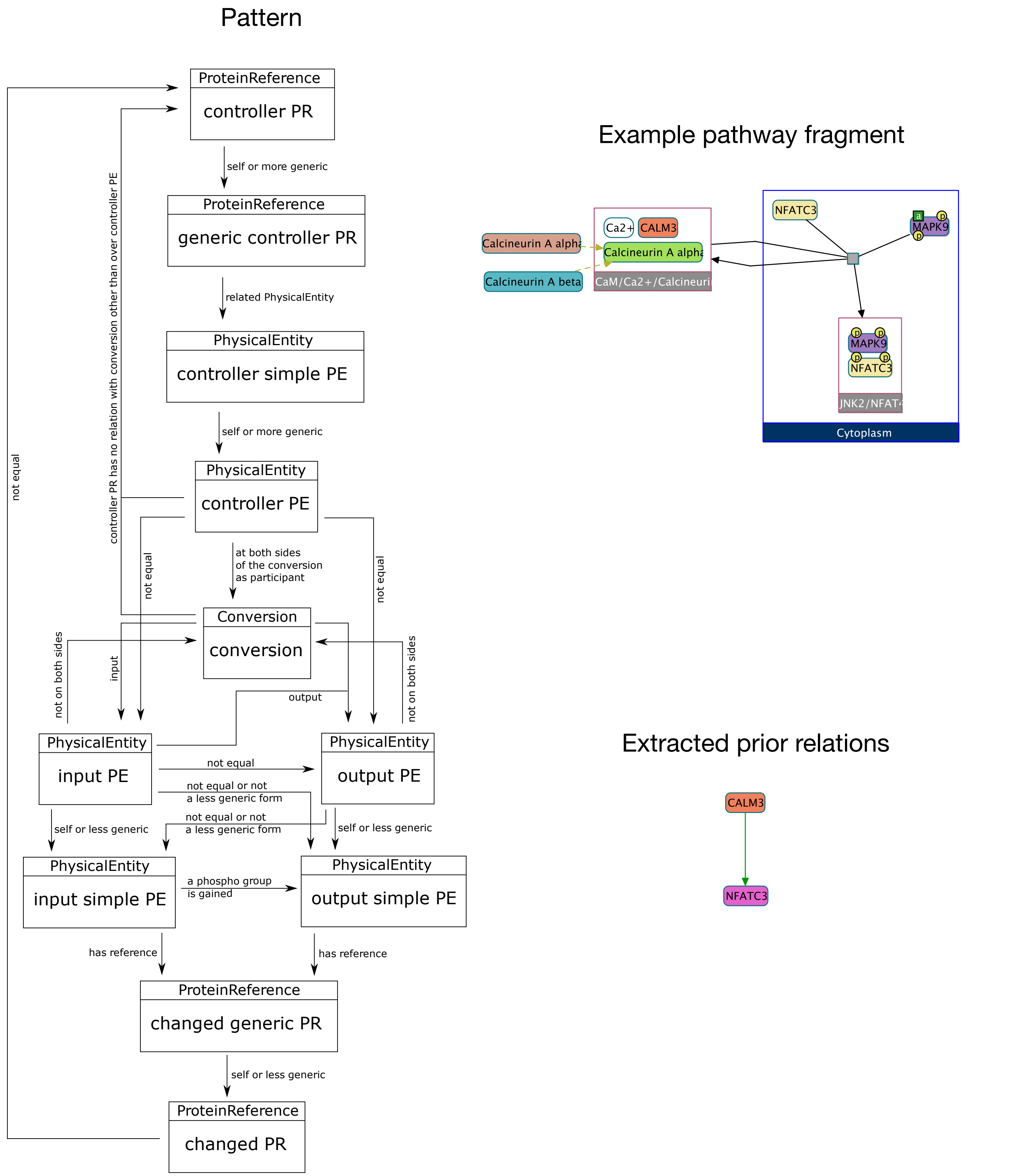

Figure S12: Pattern 2 for phosphorylation. This pattern captures the cases where the regulator proteins are not modeled as regulators of a Conversion, but modeled as inputs and outputs of the same Conversion.

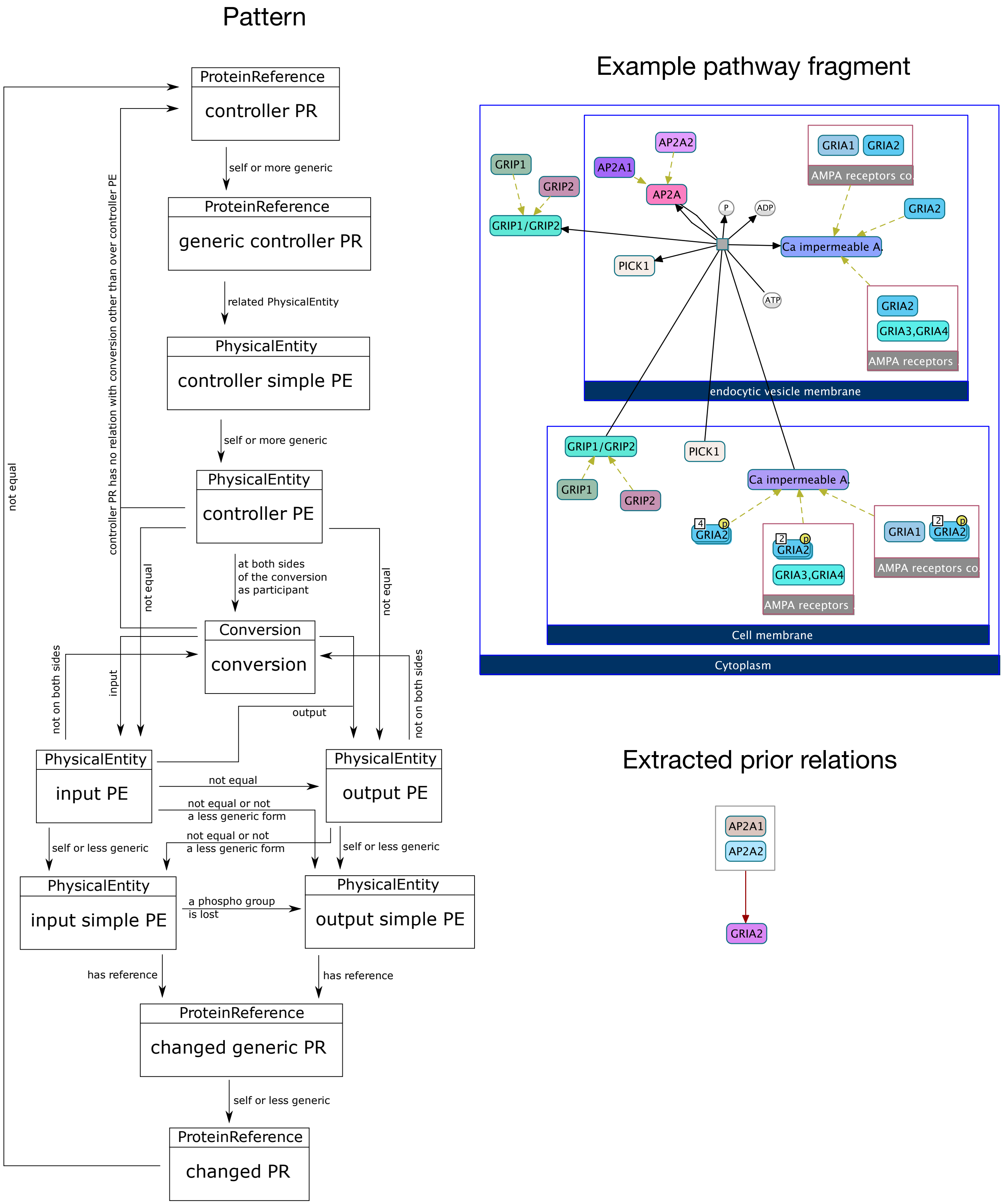

Figure S13: Pattern 2 for dephosphorylation. This pattern captures the cases where the regulator proteins are not modeled as regulators of a Conversion, but modeled as inputs and outputs of the same Conversion.

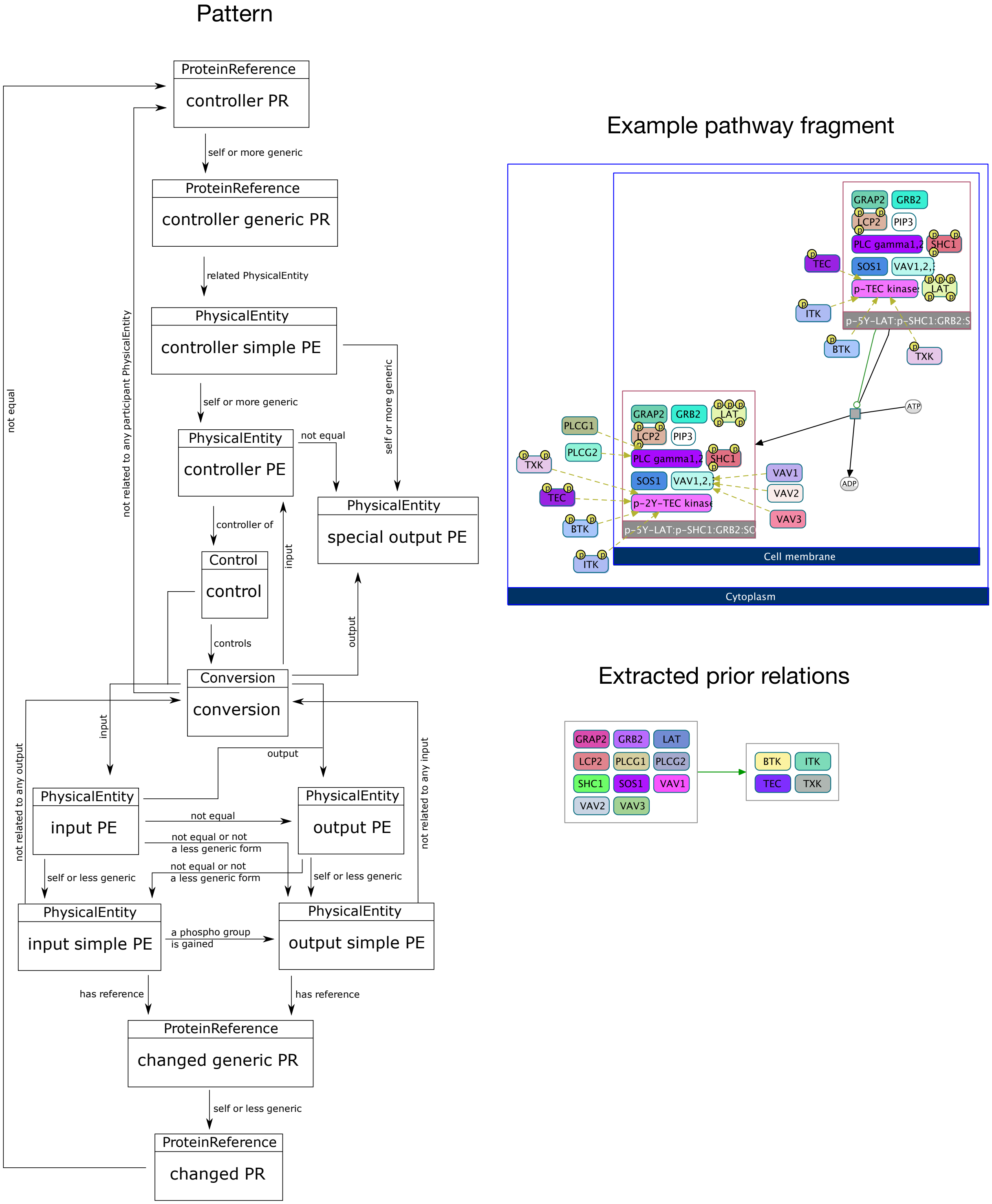

Figure S14: Pattern 3 for phosphorylation. This pattern captures the cases where some proteins in a complex are phosphorylated, and the input complex is also designated to be the controller of the Conversion.

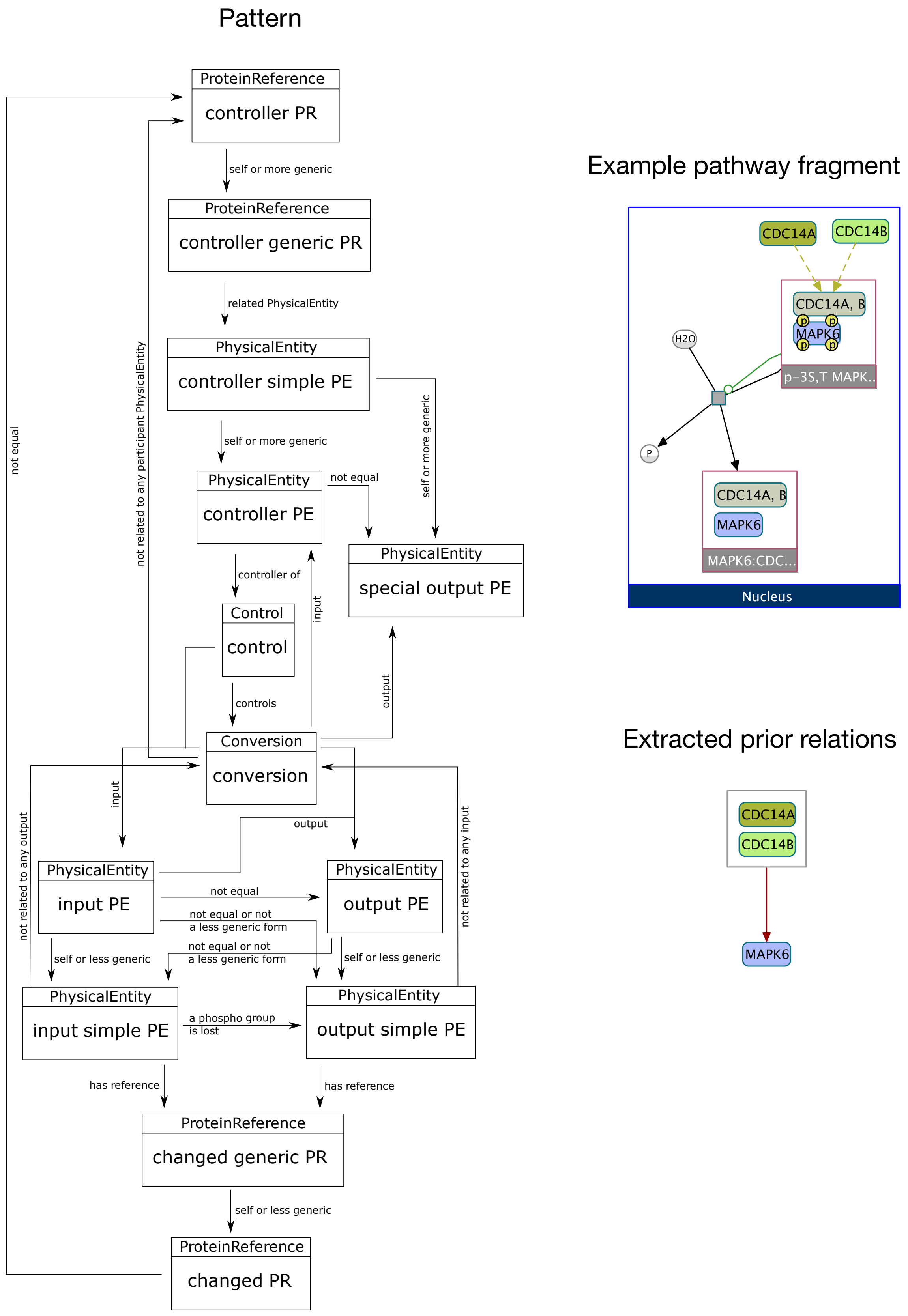

Figure S15: Pattern 3 for dephosphorylation. This pattern captures the cases where some proteins in a complex are phosphorylated, and the input complex is also designated to be the controller of the Conversion.

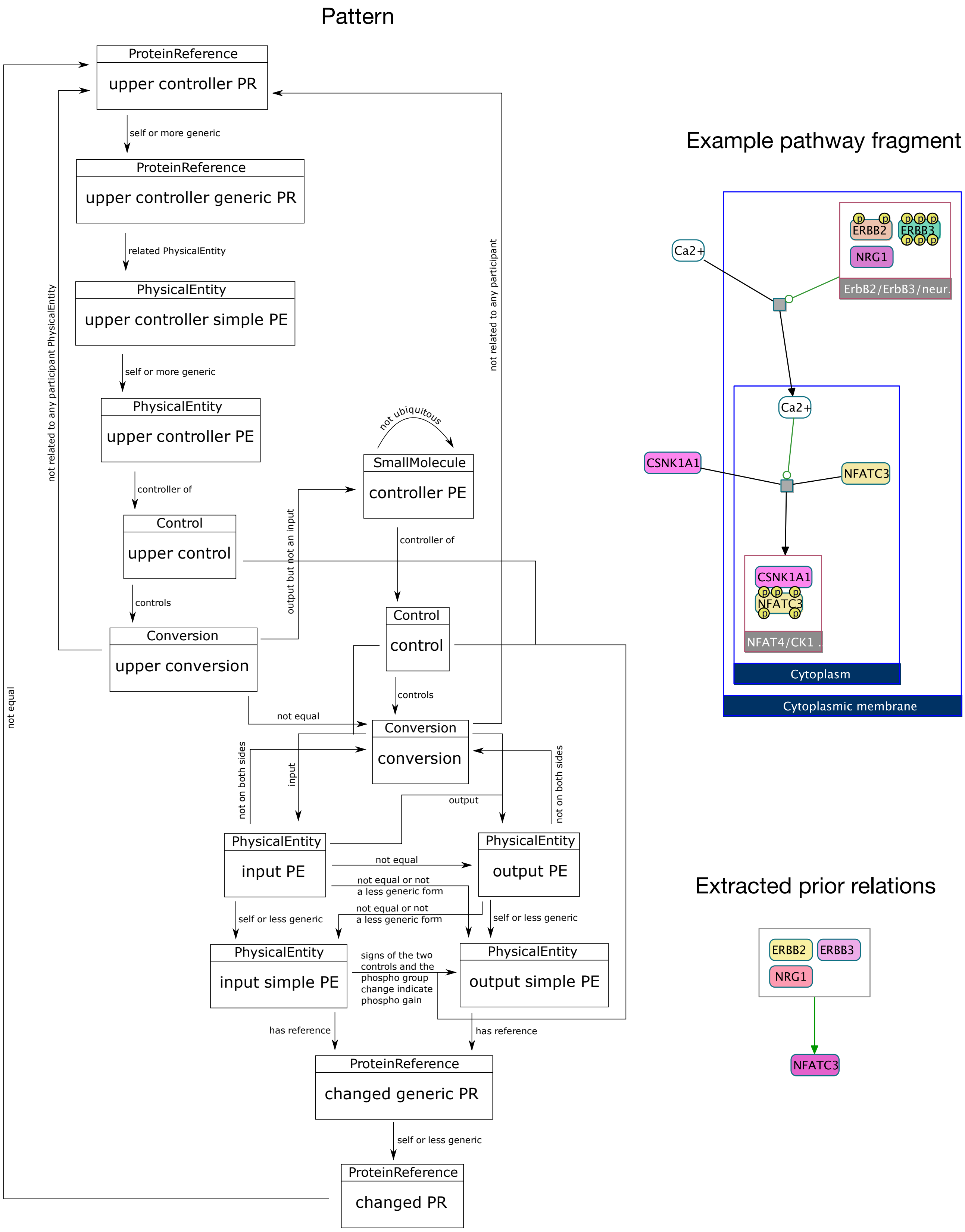

Figure S16: Pattern 4 for phosphorylation. This pattern captures the cases where the controller transmits its effect through a controlling small molecule.

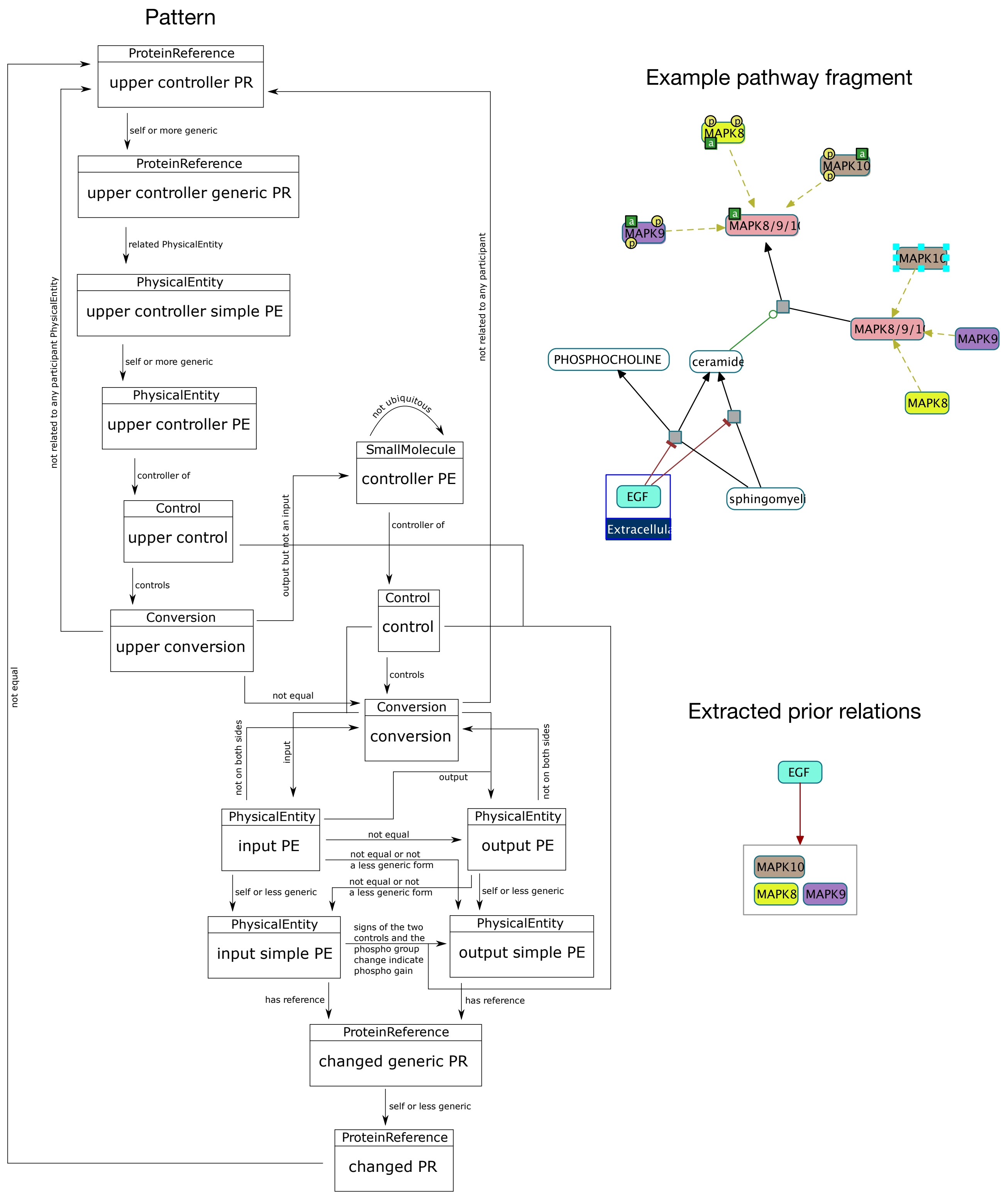

Figure S17: Pattern 4 for dephosphorylation. This pattern captures the cases where the controller transmits its effect through a controlling small molecule.

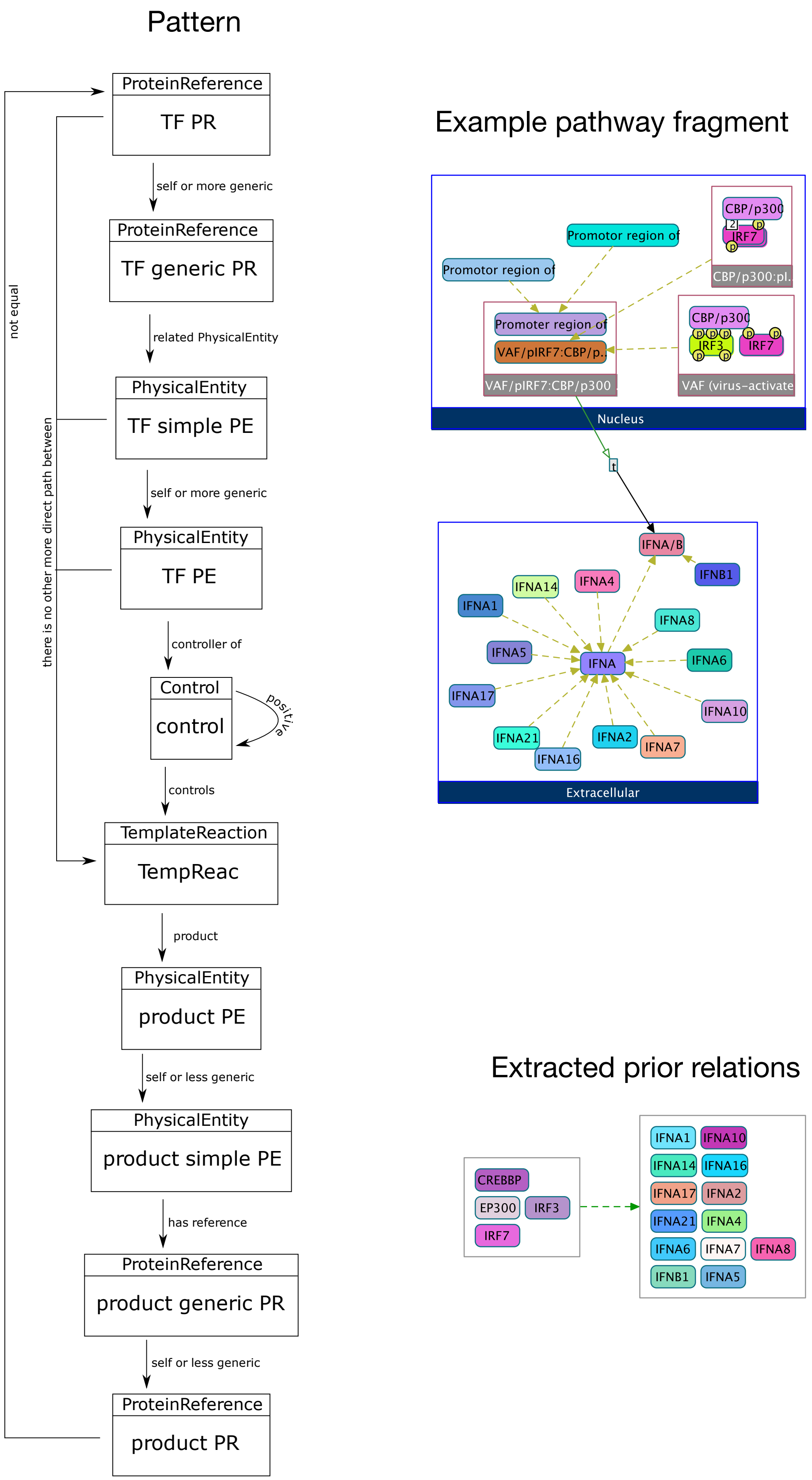

Figure S18: Pattern 1 for expression upregulation. This is the most frequent pattern for expression upregulation where the regulator activates a TemplateReaction.

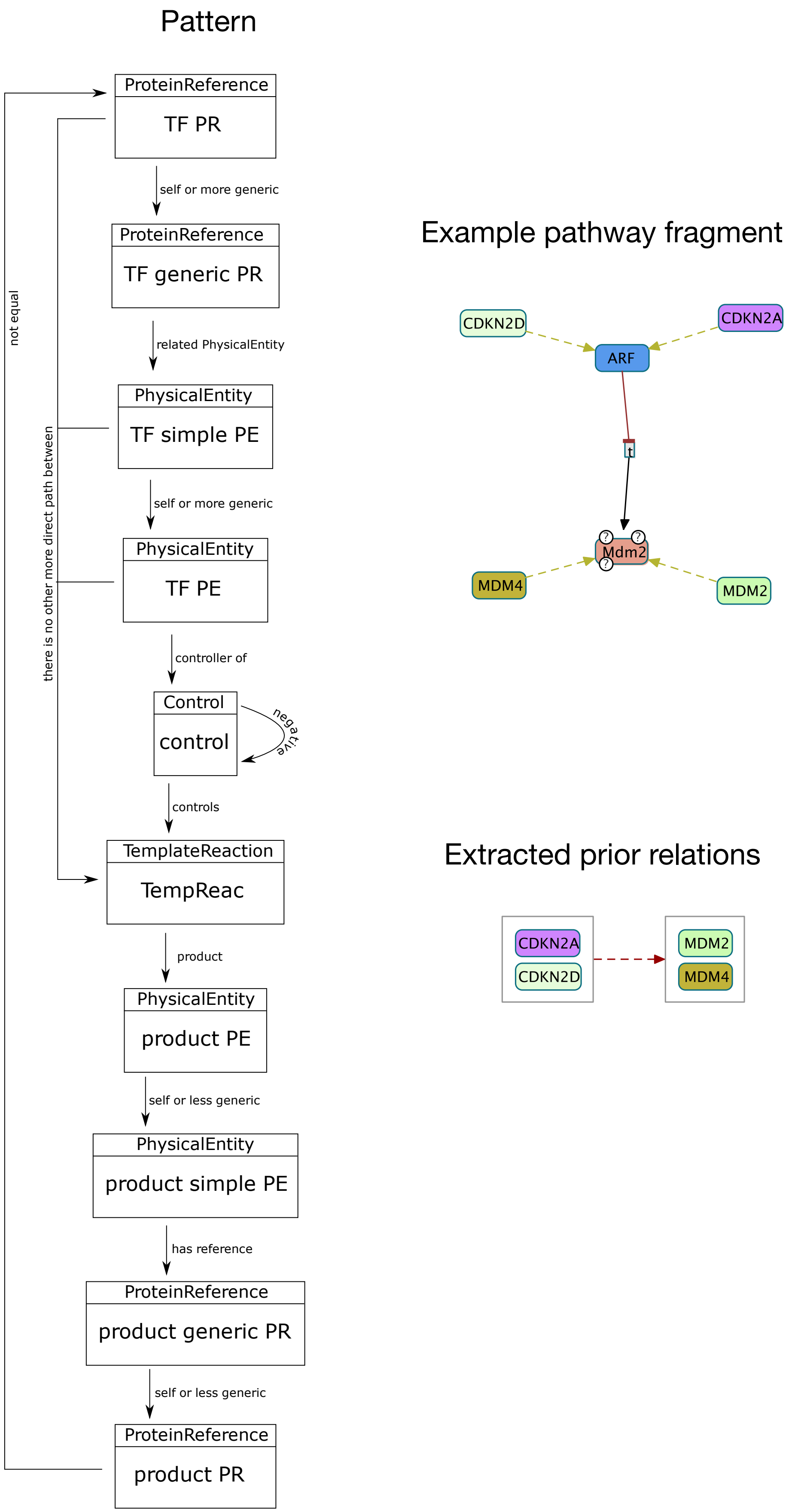

Figure S19: Pattern 1 for expression downregulation. This is the most frequent pattern for expression downregulation where the regulator inhibits a TemplateReaction.

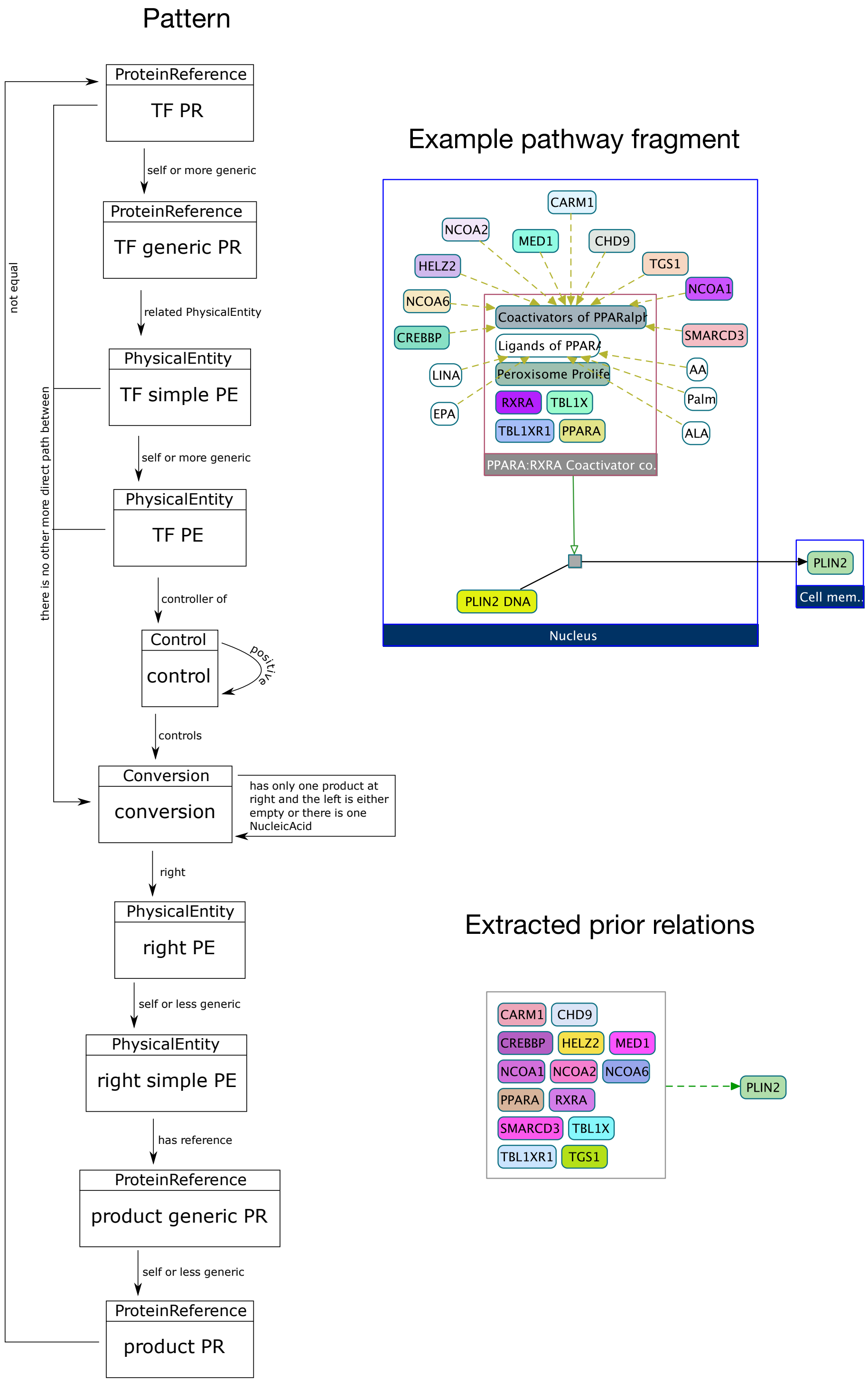

Figure S20: Pattern 2 for expression upregulation. This pattern captures the cases where a Conversion is used instead of a TemplateReaction.

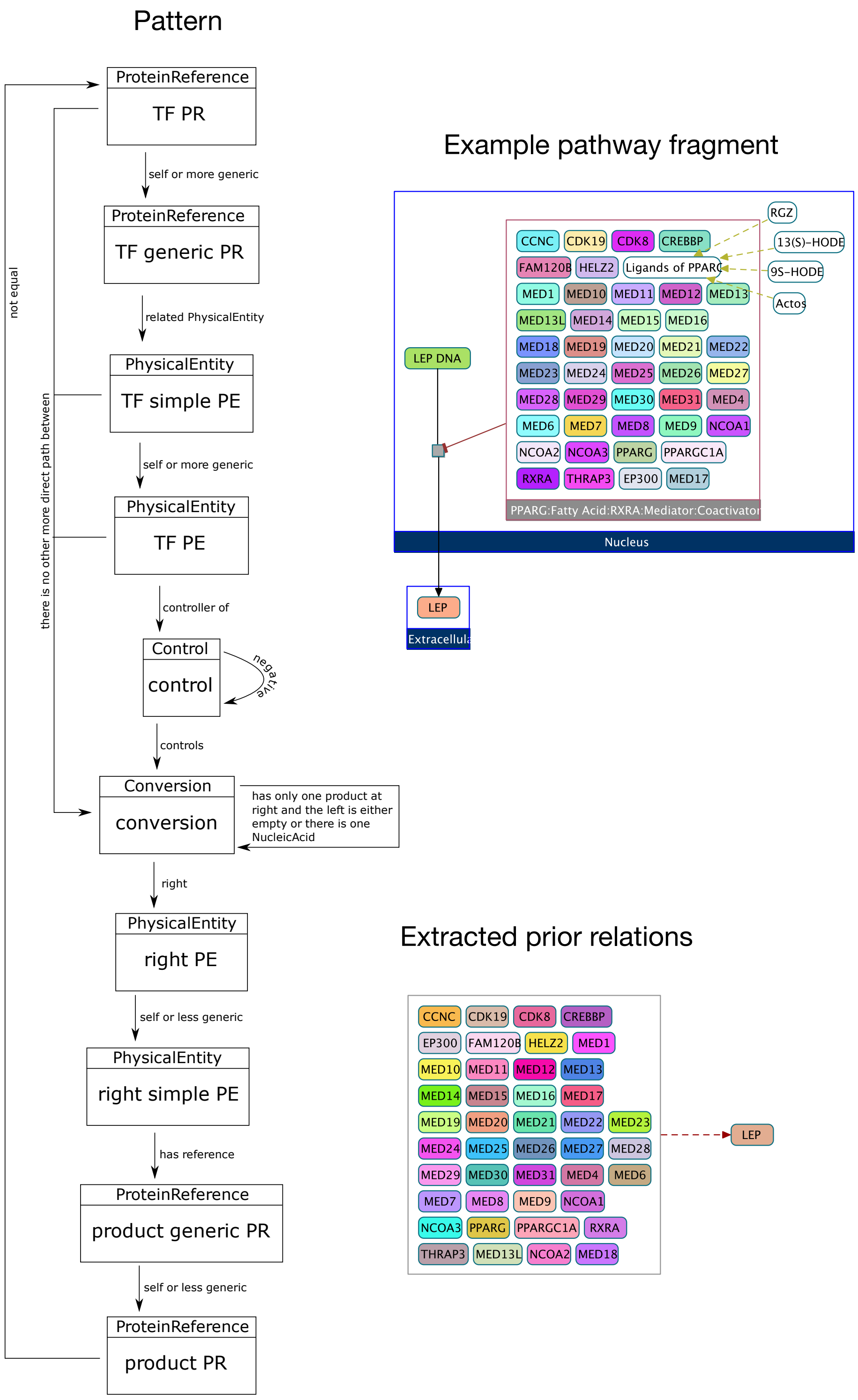

Figure S21: Pattern 2 for expression downregulation. This pattern captures the cases where a Conversion is used instead of a TemplateReaction.

3.7 Extending graphical patterns

The graphical patterns listed in this document are developed considering the available pathway relations in Pathway Commons v9, and implemented in the GitHub project at https://github.com/PathwayAndDataAnalysis/causal-priors-extractor. These patterns can be extended to either cover new data sources or to capture new relationship types. For new data sources, it is very likely that the listed patterns will be applicable as is, however not guaranteed. When needed, the graph structures in the new data source should be investigated and new graph structures should be captured by adding new patterns to the framework. Adding a new pattern can be done by adding a new class that extends from `org.biopax.paxtools.pattern.miner.MinerAdapter` (or one of its children) and override the method `constructPattern()`, just like the other patterns in the project.

The procedure is similar for capturing new types of relationships. Developers need to study the structure of the existing BioPAX data to understand in what forms the information is encoded. Then a pattern can be built to detect those structures. This process requires an understanding of BioPAX [15] and the BioPAX-pattern framework [16, https://github.com/BioPAX/Paxtools/tree/master/pattern].

References

[1] Köksal AS, Beck K, Cronin DR, McKenna A, Camp ND, Srivastava S, et al. Synthesizing signaling pathways from temporal phosphoproteomic data. Cell reports 2018;24(13):3607–18.

[2] Suppes P. A probabilistic theory of causality. North-Holland Publishing Company Amsterdam; 1970.

[3] Pearl J. Causality: models, reasoning and inference. Econometric Theory 2003;19(675-685):46.

[4] Hill SM, Nesser NK, Johnson-Camacho K, Jeffress M, Johnson A, Boniface C, et al. Context specificity in causal signaling networks revealed by phosphoprotein profiling. Cell systems 2017;4(1):73–83.

[5] Vaske CJ, Benz SC, Sanborn JZ, Earl D, Szeto C, Zhu J, et al. Inference of patient-specific pathway activities from multi-dimensional cancer genomics data using paradigm. Bioinformatics 2010;26(12):i237–45.

[6] Paull EO, Carlin DE, Niepel M, Sorger PK, Haussler D, Stuart JM. Discovering causal pathways linking genomic events to transcriptional states using tied diffusion through interacting events (tiedie). Bioinformatics 2013;29(21):2757–64.

[7] Drake JM, Paull EO, Graham NA, Lee JK, Smith BA, Titz B, et al. Phosphoproteome integration reveals patient-specific networks in prostate cancer. Cell 2016;166(4):1041–54.

[8] Melas IN, Samaga R, Alexopoulos LG, Klamt S. Detecting and removing inconsistencies between experimental data and signaling network topologies using integer linear programming on interaction graphs. PLoS computational biology 2013;9(9):e1003204.

[9] Terfve CD, Wilkes EH, Casado P, Cutillas PR, Saez-Rodriguez J. Large-scale models of signal propagation in human cells derived from discovery phosphoproteomic data. Nature communications 2015;6:8033.

[10] Chasman D, Ho YH, Berry DB, Nemec CM, MacGilvray ME, Hose J, et al. Pathway connectivity and signaling coordination in the yeast stress-activated signaling network. Molecular systems biology 2014;10(11):759.

[11] Raaijmakers LM, Giansanti P, Possik PA, Mueller J, Peeper DS, Heck AJ, et al. Phosphopath: Visualization of phosphosite-centric dynamics in temporal molecular networks. Journal of proteome research 2015;14(10):4332–41.

[12] Narushima Y, Kozuka-Hata H, Tsumoto K, Inoue JI, Oyama M. Quantitative phosphoproteomics-based molecular network description for high-resolution kinase-substrate interactome analysis. Bioinformatics 2016;:btw164.

[13] Huang J, Zhang T, Linstroth L, Tillman Z, Otegui MS, Owen HA, et al. Control of anther cell differentiation by the small protein ligand tpd1 and its receptor ems1 in arabidopsis. PLOS Genet 2016;12(8):e1006147.

[14] Rudolph JD, de Graauw M, van de Water B, Geiger T, Sharan R. Elucidation of signaling pathways from large-scale phosphoproteomic data using protein interaction networks. Cell Systems 2016;3(6):585–93.

[15] Demir E, Cary MP, Paley S, Fukuda K, Lemer C, Vastrik I, et al. The biopax community standard for pathway data sharing. Nature Biotechnology 2010;28(9):935–42.

[16] Babur Ö, Aksoy BA, Rodchenkov I, Sümer SO, Sander C, Demir E. Pattern search in BioPAX models. Bioinformatics 2014;30(1):139–40.
